# Supplementary material for: Impact of bariatric surgery on premenopausal women’s womanliness: A qualitative systematic review and meta-synthesis
Source: PLoS One. 2024 Aug 29;19(8):e0308059. doi: 10.1371/journal.pone.0308059 (PMC11361607; doi:10.1371/journal.pone.0308059)
Supplement: S2 Table — (DOCX) [file pone.0308059.s002.docx]

# Supporting information

# S2: Search strategies for qualitative articles

| **Search terms (utilizing boolean operators)** | **Databases** | **Relevant articles/Total articles** | **Comment on search (incl. synonyms)** | **Comment on result** |
| --- | --- | --- | --- | --- |
| **((((((((Women OR Woman) AND (fertility OR fecundity)) AND (bariatric surgery OR gastric bypass OR roux-en-y)) AND (sexual function OR sexuality)) AND (obesity)) AND (sex hormones OR estrogen*)) AND (experience OR experiences)) AND (hermeneutic OR phenomenological)) AND (Qualitative research OR interview OR focus groups) AND (vasomotor)** | **PubMed (Medline)**  **(Mesh-terms)** | **0/0** | **All search terms included** |  |
| **((((((((Women OR Woman) AND (fertility OR fecundity)) AND (bariatric surgery OR gastric bypass OR roux-en-y)) AND (sexual function OR sexuality)) AND (obesity)) AND (sex hormones OR estrogen*))** **AND (experience OR experiences)) AND (hermeneutic OR phenomenological)) AND (Qualitative research OR interview OR focus groups)** | **PubMed (Medline)**  **(Mesh-terms)** | **0/0** | **Removed vasomotor** |  |
| **((((((((Women OR Woman) AND (fertility OR fecundity)) AND (bariatric surgery OR gastric bypass OR roux-en-y)) AND (sexual function OR sexuality)) AND (obesity)) AND (sex hormones OR estrogen*))** **AND (experience OR experiences)) AND (Qualitative research OR interview OR focus groups)** | **PubMed (Medline)**  **(Mesh-terms)** | **0/0** | **Removed hermeneutic** |  |
| **((((((((Women OR Woman) AND (fertility OR fecundity)) AND (bariatric surgery OR gastric bypass OR roux-en-y)) AND (sexual function OR sexuality)) AND (obesity)) AND (sex hormones OR estrogen*))** **AND (Qualitative research OR interview OR focus groups)** | **PubMed (Medline)**  **(Mesh-terms)** | **1/1** | **Removed experience** | **1 relevant** |
| **((((((((Women OR Woman) AND (fertility OR fecundity)) AND (bariatric surgery OR gastric bypass OR roux-en-y)) AND (sexual function OR sexuality)) AND (obesity)) AND (sex hormones OR estrogen*))** **AND (experience OR experiences))** | **PubMed (Medline)**  **(Mesh-terms)** | **1/1** | **Removed qualitative research, add experience** | **1 relevant** |
| **((((((((Women OR Woman) AND (bariatric surgery OR gastric bypass OR roux-en-y)) AND (sexual function OR sexuality)) AND (obesity)) AND (sex hormones OR estrogen*))** **AND (experience OR experiences))** | **PubMed (Medline)**  **(Mesh-terms)** | **0/2** | **Removed fertility** | **1 quantitative, 1 duplicate** |
| **((((((((Women OR Woman) AND (bariatric surgery OR gastric bypass OR roux-en-y)) AND (sexual function OR sexuality)) AND (obesity)) AND (fertility OR fecundity))** **AND (experience OR experiences))** | **PubMed (Medline)**  **(Mesh-terms)** | **1/2** | **Removed sex hormones, add fertility** | **1 duplicate, 1 relevant** |
| **((((((((Women OR Woman) AND (bariatric surgery OR gastric bypass OR roux-en-y)) AND (obesity)) AND (fertility OR fecundity)) AND (experience OR experiences))** | **PubMed (Medline)**  **(Mesh-terms)** | **1/16** | **Remove sexual function** | **6 reviews, 5 quantitative, 1 case report, 2 duplicate, 2 relevant** |
| **((((((((Women OR Woman) AND (bariatric surgery OR gastric bypass OR roux-en-y)) AND (obesity)) AND (experience OR experiences))** | **PubMed (Medline)**  **(Mesh-terms)** | **27/345** | **Remove fertility** | **21 review, 253 quantitative, 26 case-reports, 4 duplicate, 5 concensus statement, 9 qualitative but irrelevant, 27 relevant** |
| **((((((((Women OR Woman) AND (bariatric surgery OR gastric bypass OR roux-en-y)) AND (obesity)) AND (Qualitative research OR interview OR focus groups))** | **PubMed (Medline)**  **(Mesh-terms)** | **9/130** | **Remove experiences, add qualitative research** | **3 review, 75 quantitative, 1 case-report, 1 concensus statement, 18 qualitative but irrelevant, 24 duplicate, 8 relevant** |
| **Women or Female or Woman or Females AND Fertility or Infertility or Reproductive or Fertile AND Bariatric surgery or Weight loss surgery or Gastric bypass surgery, or Y-en-Roux or Roux-en-Y AND Sexual function or Sexual dysfunction or Sexual life AND Obesity or Overweight or Fat or Obese or Unhealthy weight or High BMI AND Sex hormones or Estrogen AND Experiences or Perceptions or Attitudes or Views or Feelings or Qualitative or Perspective AND Hermeneutics AND Qualitative research or Qualitative study or Qualitative methods or Interview AND Vasomotor symptoms or Hot flushes or Hot flashes or Night sweat** | **Cinahl (Ebsco)**  **Cinahl headings** | **0/0** | **All search terms according to Cinahl headings** |  |
| **Women or Female or Woman or Females AND Fertility or Infertility or Reproductive or Fertile AND Bariatric surgery or Weight loss surgery or Gastric bypass surgery, or Y-en-Roux or Roux-en-Y AND Sexual function or Sexual dysfunction or Sexual life AND Obesity or Overweight or Fat or Obese or Unhealthy weight or High BMI AND Sex hormones or Estrogen AND Experiences or Perceptions or Attitudes or Views or Feelings or Qualitative or Perspective AND Hermeneutics AND Qualitative research or Qualitative study or Qualitative methods or Interview** | **Cinahl (Ebsco)**  **Cinahl headings** | **0/0** | **Removed vasomotor** |  |
| **Women or Female or Woman or Females AND Fertility or Infertility or Reproductive or Fertile AND Bariatric surgery or Weight loss surgery or Gastric bypass surgery, or Y-en-Roux or Roux-en-Y AND Sexual function or Sexual dysfunction or Sexual life AND Obesity or Overweight or Fat or Obese or Unhealthy weight or High BMI AND Sex hormones or Estrogen AND Experiences or Perceptions or Attitudes or Views or Feelings or Qualitative or Perspective AND Qualitative research or Qualitative study or Qualitative methods or Interview** | **Cinahl (Ebsco)**  **Cinahl headings** | **0/1** | **Removed hermeneutics** | **1 duplicate** |
| **Women or Female or Woman or Females AND Fertility or Infertility or Reproductive or Fertile AND Bariatric surgery or Weight loss surgery or Gastric bypass surgery, or Y-en-Roux or Roux-en-Y AND Sexual function or Sexual dysfunction or Sexual life AND Obesity or Overweight or Fat or Obese or Unhealthy weight or High BMI AND Sex hormones or Estrogen AND Experiences or Perceptions or Attitudes or Views or Feelings or Qualitative or Perspective** | **Cinahl (Ebsco)**  **Cinahl headings** | **0/2** | **Removed qualitative research** | **2 duplicates** |
| **Women or Female or Woman or Females AND Fertility or Infertility or Reproductive or Fertile AND Bariatric surgery or Weight loss surgery or Gastric bypass surgery, or Y-en-Roux or Roux-en-Y AND Sexual function or Sexual dysfunction or Sexual life AND Obesity or Overweight or Fat or Obese or Unhealthy weight or High BMI AND Sex hormones or Estrogen AND Qualitative research or Qualitative study or Qualitative methods or Interview** | **Cinahl (Ebsco)**  **Cinahl headings** | **0/1** | **Removed experience, add qualitative research** | **1 duplicate** |
| **Women or Female or Woman or Females AND Bariatric surgery or Weight loss surgery or Gastric bypass surgery, or Y-en-Roux or Roux-en-Y AND Sexual function or Sexual dysfunction or Sexual life AND Obesity or Overweight or Fat or Obese or Unhealthy weight or High BMI AND Sex hormones or Estrogen AND Qualitative research or Qualitative study or Qualitative methods or Interview** | **Cinahl (Ebsco)**  **Cinahl headings** | **0/2** | **Removed fertility** | **1 duplicate, 1 quantitative** |
| **Women or Female or Woman or Females AND Bariatric surgery or Weight loss surgery or Gastric bypass surgery, or Y-en-Roux or Roux-en-Y AND Obesity or Overweight or Fat or Obese or Unhealthy weight or High BMI AND Sex hormones or Estrogen AND Qualitative research or Qualitative study or Qualitative methods or Interview** | **Cinahl (Ebsco)**  **Cinahl headings** | **0/2** | **Remove sexual function** | **1 duplicate, 1 quantitative** |
| **Women or Female or Woman or Females AND Bariatric surgery or Weight loss surgery or Gastric bypass surgery, or Y-en-Roux or Roux-en-Y AND Sexual function or Sexual dysfunction or Sexual life AND Obesity or Overweight or Fat or Obese or Unhealthy weight or High BMI AND Qualitative research or Qualitative study or Qualitative methods or Interview** | **Cinahl (Ebsco)**  **Cinahl headings** | **1/21** | **Remove sex hormones, add sexual function** | **12 quantitative, 3 qualitative but irrelevant, 5 duplicates, 1 relevant** |
| **Women or Female or Woman or Females AND Bariatric surgery or Weight loss surgery or Gastric bypass surgery, or Y-en-Roux or Roux-en-Y AND Sexual function or Sexual dysfunction or Sexual life AND Obesity or Overweight or Fat or Obese or Unhealthy weight or High BMI AND Experiences or Perceptions or Attitudes or Views or Feelings or Qualitative or Perspective** | **Cinahl (Ebsco)**  **Cinahl headings** | **0/49** | **Removed qualitative research, add experience** | **39 quantitative, 7 duplicate, 2 qualitative but irrelevant, 1 review** |
| **Women or Female or Woman or Females AND Bariatric surgery or Weight loss surgery or Gastric bypass surgery, or Y-en-Roux or Roux-en-Y AND Obesity or Overweight or Fat or Obese or Unhealthy weight or High BMI AND Sex hormones or Estrogen AND Experiences or Perceptions or Attitudes or Views or Feelings or Qualitative or Perspective** | **Cinahl (Ebsco)**  **Cinahl headings** | **0/3** | **Removed sexual function, added sex hormones** | **1 quantitative, 2 duplicate** |
| **Women or Female or Woman or Females AND Bariatric surgery or Weight loss surgery or Gastric bypass surgery, or Y-en-Roux or Roux-en-Y AND Obesity or Overweight or Fat or Obese or Unhealthy weight or High BMI AND Experiences or Perceptions or Attitudes or Views or Feelings or Qualitative or Perspective AND Fertility or Infertility or Reproductive or Fertile** | **Cinahl (Ebsco)**  **Cinahl headings** | **1/18** | **Removed sex hormones, added fertility** | **6 duplicates, 9 quantitative, 2 review, 1 relevant** |
| **Women or Female or Woman or Females AND Bariatric surgery or Weight loss surgery or Gastric bypass surgery, or Y-en-Roux or Roux-en-Y AND Obesity or Overweight or Fat or Obese or Unhealthy weight or High BMI AND Fertility or Infertility or Reproductive or Fertile AND Qualitative research or Qualitative study or Qualitative methods or Interview** | **Cinahl (Ebsco)**  **Cinahl headings** | **0/7** | **Removed experience, added qualitative research** | **6 duplicates, 1 quantitative** |
| **Women or Female or Woman or Females AND Bariatric surgery or Weight loss surgery or Gastric bypass surgery, or Y-en-Roux or Roux-en-Y AND Obesity or Overweight or Fat or Obese or Unhealthy weight or High BMI AND Qualitative research or Qualitative study or Qualitative methods or Interview** | **Cinahl (Ebsco)**  **Cinahl headings** | **21/189** | **Removed fertility** | **101 quantitative, 1 review, 1 concensus statement, 39 qualitative but irrelevant, 2 case reports, 24 duplicates,**  **21 relevant** |
| **Women or Female or Woman or Females AND Bariatric surgery or Weight loss surgery or Gastric bypass surgery, or Y-en-Roux or Roux-en-Y AND Obesity or Overweight or Fat or Obese or Unhealthy weight or High BMI AND Experiences or Perceptions or Attitudes or Views or Feelings or Qualitative or Perspective** | **Cinahl (Ebsco)**  **Cinahl headings** | **546** | **Remove qualitative research, added experience** | **High yield, add limits for automated screening** |
| **Women or Female or Woman or Females AND Bariatric surgery or Weight loss surgery or Gastric bypass surgery, or Y-en-Roux or Roux-en-Y AND Obesity or Overweight or Fat or Obese or Unhealthy weight or High BMI AND Experiences or Perceptions or Attitudes or Views or Feelings or Qualitative or Perspective** | **Cinahl (Ebsco)**  **Cinahl headings** | **287** | **Added limits: female, 2000-2022, human, english, age 19-44** | **High yield, add more limits for automated screening** |
| **Women or Female or Woman or Females AND Bariatric surgery or Weight loss surgery or Gastric bypass surgery, or Y-en-Roux or Roux-en-Y AND Obesity or Overweight or Fat or Obese or Unhealthy weight or High BMI AND Experiences or Perceptions or Attitudes or Views or Feelings or Qualitative or Perspective** | **Cinahl (Ebsco)**  **Cinahl headings** | **0/217** | **Added limits: Qualitative research** | **174 quantitative, 15 qualitative but irrelevant, 1 case report, 27 duplicates 1 quant, 1 duplicate** |
| **Female AND Fertility AND ‘Bariatric surgery’ AND ‘Sexual function’ AND Obesity AND ‘Sex hormones’ AND Experiences AND Hermeneutics AND ‘Qualitative research’ AND Hot flush** | **Embase (Ovid)**  **Emtree** | **0/0** |  |  |
| **Female AND Fertility AND ‘Bariatric surgery’ AND ‘Sexual function’ AND Obesity AND ‘Sex hormones’ AND Experiences AND Hermeneutics AND ‘Qualitative research’** | **Embase (Ovid)**  **Emtree** | **0/0** | **Removed hot flush** |  |
| **Female AND Fertility AND ‘Bariatric surgery’ AND ‘Sexual function’ AND Obesity AND ‘Sex hormones’ AND Experiences AND ‘Qualitative research’** | **Embase (Ovid)**  **Emtree** | **0/0** | **Removed hermeneutics** |  |
| **Female AND Fertility AND ‘Bariatric surgery’ AND ‘Sexual function’ AND Obesity AND ‘Sex hormones’ AND ‘Qualitative research’** | **Embase (Ovid)**  **Emtree** | **0/0** | **Removed experience** |  |
| **Female AND ‘Bariatric surgery’ AND ‘Sexual function’ AND Obesity AND ‘Sex hormones’ AND ‘Qualitative research’** | **Embase (Ovid)**  **Emtree** | **0/0** | **Removed fertility** |  |
| **Female AND ‘Bariatric surgery’ AND Obesity AND ‘Sex hormones’ AND ‘Qualitative research’** | **Embase (Ovid)**  **Emtree** | **0/2** | **Removed sexual function** | **1 quantitative, 1 duplicate** |
| **Female AND ‘Bariatric surgery’ AND Obesity AND ‘Sex hormones’ AND Experiences** | **Embase (Ovid)**  **Emtree** | **0/15** | **Removed qualitative research, added experience** | **10 quantitative, 1 duplicate, 1 qualitative but irrelevant, 3 review** |
| **Female AND ‘Bariatric surgery’ AND Obesity AND ‘Sexual function’ AND Experiences** | **Embase (Ovid)**  **Emtree** | **0/11** | **Removed sex hormones, added sexual function** | **10 quantitative, 1 duplicate** |
| **Female AND ‘Bariatric surgery’ AND Obesity AND Experiences** | **Embase (Ovid)**  **Emtree** | **3052** | **Removed sexual function** | **Need to add limits for automated screening** |
| **Female AND ‘Bariatric surgery’ AND Obesity AND Experiences** | **Embase (Ovid)**  **Emtree** | **3/208** | **Add limits: Embase only, female, adult, qual, interview** | **188 quantitative, 2 consensus reports, 4 reviews, 2 duplicates, 9 qualitative but irrelevant, 3 relevant** |
| **Women or Female or Woman or Females AND Fertility or Infertility or Reproductive or Fertile AND Bariatric surgery or Weight loss surgery or Gastric bypass surgery, or Y-en-Roux or Roux-en-Y AND Sexual function or Sexual dysfunction or Sexual life AND Obesity or Overweight or Fat or Obese or Unhealthy weight or High BMI AND Sex hormones or Estrogen AND Experiences or Perceptions or Attitudes or Views or Feelings or Qualitative or Perspective AND Hermeneutics AND Qualitative research or Qualitative study or Qualitative methods or Interview AND Vasomotor symptoms or Hot flushes or Hot flashes or Night sweat** | **PsycInfo (EBSCO)** | **0/0** |  |  |
| **Women or Female or Woman or Females AND Fertility or Infertility or Reproductive or Fertile AND Bariatric surgery or Weight loss surgery or Gastric bypass surgery, or Y-en-Roux or Roux-en-Y AND Sexual function or Sexual dysfunction or Sexual life AND Obesity or Overweight or Fat or Obese or Unhealthy weight or High BMI AND Sex hormones or Estrogen AND Experiences or Perceptions or Attitudes or Views or Feelings or Qualitative or Perspective AND Hermeneutics AND Qualitative research or Qualitative study or Qualitative methods or Interview** | **PsycInfo (EBSCO)** | **0/0** | **Removed vasomotor** |  |
| **Women or Female or Woman or Females AND Fertility or Infertility or Reproductive or Fertile AND Bariatric surgery or Weight loss surgery or Gastric bypass surgery, or Y-en-Roux or Roux-en-Y AND Sexual function or Sexual dysfunction or Sexual life AND Obesity or Overweight or Fat or Obese or Unhealthy weight or High BMI AND Sex hormones or Estrogen AND Experiences or Perceptions or Attitudes or Views or Feelings or Qualitative or Perspective AND Qualitative research or Qualitative study or Qualitative methods or Interview** | **PsycInfo (EBSCO)** | **0/0** | **Removed hermeneutics** |  |
| **Women or Female or Woman or Females AND Fertility or Infertility or Reproductive or Fertile AND Bariatric surgery or Weight loss surgery or Gastric bypass surgery, or Y-en-Roux or Roux-en-Y AND Sexual function or Sexual dysfunction or Sexual life AND Obesity or Overweight or Fat or Obese or Unhealthy weight or High BMI AND Sex hormones or Estrogen AND Qualitative research or Qualitative study or Qualitative methods or Interview** | **PsycInfo (EBSCO)** | **0/0** | **Removed experience** |  |
| **Women or Female or Woman or Females AND Bariatric surgery or Weight loss surgery or Gastric bypass surgery, or Y-en-Roux or Roux-en-Y AND Sexual function or Sexual dysfunction or Sexual life AND Obesity or Overweight or Fat or Obese or Unhealthy weight or High BMI AND Sex hormones or Estrogen AND Qualitative research or Qualitative study or Qualitative methods or Interview** | **PsycInfo (EBSCO)** | **0/0** | **Removed fertility** |  |
| **Women or Female or Woman or Females AND Bariatric surgery or Weight loss surgery or Gastric bypass surgery, or Y-en-Roux or Roux-en-Y AND Obesity or Overweight or Fat or Obese or Unhealthy weight or High BMI AND Sex hormones or Estrogen AND Qualitative research or Qualitative study or Qualitative methods or Interview** | **PsycInfo (EBSCO)** | **0/0** | **Removed sexual function** |  |
| **Women or Female or Woman or Females AND Bariatric surgery or Weight loss surgery or Gastric bypass surgery, or Y-en-Roux or Roux-en-Y AND Obesity or Overweight or Fat or Obese or Unhealthy weight or High BMI AND Sex hormones or Estrogen AND Experiences or Perceptions or Attitudes or Views or Feelings or Qualitative or Perspective** | **PsycInfo (EBSCO)** | **0/1** | **Removed qualitative research, added experience** | **1 quantitative** |
| **Women or Female or Woman or Females AND Bariatric surgery or Weight loss surgery or Gastric bypass surgery, or Y-en-Roux or Roux-en-Y AND Obesity or Overweight or Fat or Obese or Unhealthy weight or High BMI AND Experiences or Perceptions or Attitudes or Views or Feelings or Qualitative or Perspective AND Sexual function or Sexual dysfunction or Sexual life** | **PsycInfo (EBSCO)** | **1/40** | **Removed sex hormones, add sexual function** | **29 quantitative, 1 qualitative but irrelevant, 5 duplicate, 4 review, 1 relevant** |
| **Women or Female or Woman or Females AND Bariatric surgery or Weight loss surgery or Gastric bypass surgery, or Y-en-Roux or Roux-en-Y AND Obesity or Overweight or Fat or Obese or Unhealthy weight or High BMI AND Experiences or Perceptions or Attitudes or Views or Feelings or Qualitative or Perspective AND Fertility or Infertility or Reproductive or Fertile** | **PsycInfo (EBSCO)** | **0/2** | **Removed sexual function, added fertility** | **2 quantitative** |
| **Women or Female or Woman or Females AND Bariatric surgery or Weight loss surgery or Gastric bypass surgery, or Y-en-Roux or Roux-en-Y AND Obesity or Overweight or Fat or Obese or Unhealthy weight or High BMI AND Fertility or Infertility or Reproductive or Fertile AND Qualitative research or Qualitative study or Qualitative methods or Interview** | **PsycInfo (EBSCO)** | **0/0** | **Removed experience, add qualitative research** |  |
| **Women or Female or Woman or Females AND Bariatric surgery or Weight loss surgery or Gastric bypass surgery, or Y-en-Roux or Roux-en-Y AND Obesity or Overweight or Fat or Obese or Unhealthy weight or High BMI AND Qualitative research or Qualitative study or Qualitative methods or Interview AND Sexual function or Sexual dysfunction or Sexual life** | **PsycInfo (EBSCO)** | **0/26** | **Removed fertility, added sexual function** | **5 duplicates, 20 quantitative, 1 review** |
| **Women or Female or Woman or Females AND Bariatric surgery or Weight loss surgery or Gastric bypass surgery, or Y-en-Roux or Roux-en-Y AND Obesity or Overweight or Fat or Obese or Unhealthy weight or High BMI AND Qualitative research or Qualitative study or Qualitative methods or Interview** | **PsycInfo (EBSCO)** | **5/184** | **Removed sexual function** | **25 duplicates, 120 quantitative, 5 reviews, 29 qualitative but irrelevant, 1 consensus statement, 1 case report, 4 relevant** |
| **Women or Female or Woman or Females AND Fertility or Infertility or Reproductive or Fertile AND Bariatric surgery or Weight loss surgery or Gastric bypass surgery, or Y-en-Roux or Roux-en-Y AND Sexual function or Sexual dysfunction or Sexual life AND Obesity or Overweight or Fat or Obese or Unhealthy weight or High BMI AND Sex hormones or Estrogen AND Experiences or Perceptions or Attitudes or Views or Feelings or Qualitative or Perspective AND Hermeneutics AND Qualitative research or Qualitative study or Qualitative methods or Interview AND Vasomotor symptoms or Hot flushes or Hot flashes or Night sweat** | **PsycArticles (EBSCO)** | **0/0** |  |  |
| **Women or Female or Woman or Females AND Fertility or Infertility or Reproductive or Fertile AND Bariatric surgery or Weight loss surgery or Gastric bypass surgery, or Y-en-Roux or Roux-en-Y AND Sexual function or Sexual dysfunction or Sexual life AND Obesity or Overweight or Fat or Obese or Unhealthy weight or High BMI AND Sex hormones or Estrogen AND Experiences or Perceptions or Attitudes or Views or Feelings or Qualitative or Perspective AND Hermeneutics AND Qualitative research or Qualitative study or Qualitative methods or Interview** | **PsycArticles (EBSCO)** | **0/0** | **Removed vasomotor** |  |
| **Women or Female or Woman or Females AND Fertility or Infertility or Reproductive or Fertile AND Bariatric surgery or Weight loss surgery or Gastric bypass surgery, or Y-en-Roux or Roux-en-Y AND Sexual function or Sexual dysfunction or Sexual life AND Obesity or Overweight or Fat or Obese or Unhealthy weight or High BMI AND Sex hormones or Estrogen AND Experiences or Perceptions or Attitudes or Views or Feelings or Qualitative or Perspective AND Qualitative research or Qualitative study or Qualitative methods or Interview** | **PsycArticles**  **(EBSCO)** | **0/0** | **Removed hermeneutic** |  |
| **Women or Female or Woman or Females AND Fertility or Infertility or Reproductive or Fertile AND Bariatric surgery or Weight loss surgery or Gastric bypass surgery, or Y-en-Roux or Roux-en-Y AND Sexual function or Sexual dysfunction or Sexual life AND Obesity or Overweight or Fat or Obese or Unhealthy weight or High BMI AND Sex hormones or Estrogen AND Qualitative research or Qualitative study or Qualitative methods or Interview** | **PsycArticles (EBSCO)** | **0/0** | **Removed experience** |  |
| **Women or Female or Woman or Females AND Bariatric surgery or Weight loss surgery or Gastric bypass surgery, or Y-en-Roux or Roux-en-Y AND Sexual function or Sexual dysfunction or Sexual life AND Obesity or Overweight or Fat or Obese or Unhealthy weight or High BMI AND Sex hormones or Estrogen AND Qualitative research or Qualitative study or Qualitative methods or Interview** | **PsycArticles (EBSCO)** | **0/0** | **Removed fertility** |  |
| **Women or Female or Woman or Females AND Bariatric surgery or Weight loss surgery or Gastric bypass surgery, or Y-en-Roux or Roux-en-Y AND Obesity or Overweight or Fat or Obese or Unhealthy weight or High BMI AND Sex hormones or Estrogen AND Qualitative research or Qualitative study or Qualitative methods or Interview** | **PsycArticles (EBSCO)** | **0/0** | **Removed sexual function** |  |
| **Women or Female or Woman or Females AND Bariatric surgery or Weight loss surgery or Gastric bypass surgery, or Y-en-Roux or Roux-en-Y AND Obesity or Overweight or Fat or Obese or Unhealthy weight or High BMI AND Sex hormones or Estrogen AND Experiences or Perceptions or Attitudes or Views or Feelings or Qualitative or Perspective** | **PsycArticles (EBSCO)** | **0/0** | **Removed qualitative research, added experience** |  |
| **Women or Female or Woman or Females AND Bariatric surgery or Weight loss surgery or Gastric bypass surgery, or Y-en-Roux or Roux-en-Y AND Obesity or Overweight or Fat or Obese or Unhealthy weight or High BMI AND Experiences or Perceptions or Attitudes or Views or Feelings or Qualitative or Perspective AND Sexual function or Sexual dysfunction or Sexual life** | **PsycArticles (EBSCO)** | **0/1** | **Removed sex hormones, added sexual function** | **1 quantitative** |
| **Women or Female or Woman or Females AND Bariatric surgery or Weight loss surgery or Gastric bypass surgery, or Y-en-Roux or Roux-en-Y AND Obesity or Overweight or Fat or Obese or Unhealthy weight or High BMI AND Experiences or Perceptions or Attitudes or Views or Feelings or Qualitative or Perspective AND Fertility or Infertility or Reproductive or Fertile** | **PsycArticles (EBSCO)** | **0/0** | **Removed sexual function, added fertility** |  |
| **Women or Female or Woman or Females AND Bariatric surgery or Weight loss surgery or Gastric bypass surgery, or Y-en-Roux or Roux-en-Y AND Obesity or Overweight or Fat or Obese or Unhealthy weight or High BMI AND Fertility or Infertility or Reproductive or Fertile AND Qualitative research or Qualitative study or Qualitative methods or Interview** | **PsycArticles (EBSCO)** | **0/0** | **Removed experience, added qualitative research** |  |
| **Women or Female or Woman or Females AND Bariatric surgery or Weight loss surgery or Gastric bypass surgery, or Y-en-Roux or Roux-en-Y AND Obesity or Overweight or Fat or Obese or Unhealthy weight or High BMI AND Qualitative research or Qualitative study or Qualitative methods or Interview AND Sexual function or Sexual dysfunction or Sexual life** | **PsycArticles (EBSCO)** | **0/0** | **Removed fertility, added sexual function** |  |
| **Women or Female or Woman or Females AND Bariatric surgery or Weight loss surgery or Gastric bypass surgery, or Y-en-Roux or Roux-en-Y AND Obesity or Overweight or Fat or Obese or Unhealthy weight or High BMI AND Qualitative research or Qualitative study or Qualitative methods or Interview AND Vasomotor symptoms or Hot flushes or Hot flashes or Night sweat** | **PsycArticles (EBSCO)** | **0/0** | **Removed sexual function, added vasomotor** |  |
| **Women or Female or Woman or Females AND Bariatric surgery or Weight loss surgery or Gastric bypass surgery, or Y-en-Roux or Roux-en-Y AND Obesity or Overweight or Fat or Obese or Unhealthy weight or High BMI AND Qualitative research or Qualitative study or Qualitative methods or Interview** | **PsycArticles (EBSCO)** | **0/2** | **Removed vasomotor** | **2 quantitative** |
| **TITLE-ABS-KEY ( women OR woman OR female AND obesity AND bariatric AND surgery OR gastric AND bypass OR roux-en-y AND qualitative AND research OR interview OR perspective OR lived AND experience AND experience AND fertility AND sex AND hormones OR estrogen AND sexual AND function AND hermeneutic AND vasomotor)** | **Scopus** | **0/0** |  |  |
| **TITLE-ABS-KEY ( women OR woman OR female AND obesity AND bariatric AND surgery OR gastric AND bypass OR roux-en-y AND qualitative AND research OR interview OR perspective OR lived AND experience AND experience AND fertility AND sex AND hormones OR estrogen AND sexual AND function AND hermeneutic)** | **Scopus** | **0/0** | **Remove vasomotor** |  |
| **TITLE-ABS-KEY ( women OR woman OR female AND obesity AND bariatric AND surgery OR gastric AND bypass OR roux-en-y AND qualitative AND research OR interview OR perspective OR lived AND experience AND experience AND fertility AND sex AND hormones OR estrogen AND sexual AND function)** | **Scopus** | **0/0** | **Removed hermeneutics** |  |
| **TITLE-ABS-KEY ( women OR woman OR female AND obesity AND bariatric AND surgery OR gastric AND bypass OR roux-en-y AND qualitative AND research OR interview OR perspective OR lived AND fertility AND sex AND hormones OR estrogen AND sexual AND function)** | **Scopus** | **0/0** | **Removed experience** |  |
| **TITLE-ABS-KEY ( women OR woman OR female AND obesity AND bariatric AND surgery OR gastric AND bypass OR roux-en-y AND qualitative AND research OR interview OR perspective OR lived AND sex AND hormones OR estrogen AND sexual AND function)** | **Scopus** | **0/0** | **Removed fertility** |  |
| **TITLE-ABS-KEY ( women OR woman OR female AND obesity AND bariatric AND surgery OR gastric AND bypass OR roux-en-y AND qualitative AND research OR interview OR perspective OR lived AND sex AND hormones OR estrogen)** | **Scopus** | **0/0** | **Removed sexual function** |  |
| **TITLE-ABS-KEY ( women OR woman OR female AND obesity AND bariatric AND surgery OR gastric AND bypass OR roux-en-y AND experience AND experience AND sex AND hormones OR estrogen)** | **Scopus** | **0/1** | **Removed qualitative research, added experience** |  |
| **TITLE-ABS-KEY ( women OR woman OR female AND obesity AND bariatric AND surgery OR gastric AND bypass OR roux-en-y AND experience AND experience AND sexual AND function)** | **Scopus** | **0/0** | **Removed sex hormones, added sexual function** |  |
| **TITLE-ABS-KEY ( women OR woman OR female AND obesity AND bariatric AND surgery OR gastric AND bypass OR roux-en-y AND experience AND experience AND fertility)** | **Scopus** | **0/6** | **Removed sexual function, added fertility** |  |
| **TITLE-ABS-KEY ( women OR woman OR female AND obesity AND bariatric AND surgery OR gastric AND bypass OR roux-en-y AND experience AND experience)** | **Scopus** | **0/0** | **Removed fertility** |  |
| **TITLE-ABS-KEY ( women OR woman OR female AND obesity AND bariatric AND surgery OR gastric AND bypass OR roux-en-y AND fertility AND qualitative AND research)** | **Scopus** | **0/0** | **Removed experience, added fertility and qualitative research** |  |
| **TITLE-ABS-KEY ( women OR woman OR female AND obesity AND bariatric AND surgery OR gastric AND bypass OR roux-en-y AND qualitative AND research AND sexual AND function)** | **Scopus** | **0/0** | **Removed fertility, added sexual function** |  |
| **TITLE-ABS-KEY ( women OR woman OR female AND obesity AND bariatric AND surgery OR gastric AND bypass OR roux-en-y AND qualitative AND research AND vasomotor)** | **Scopus** | **0/0** | **Removed sexual function, added vasomotor** |  |
| **TITLE-ABS-KEY ( women OR woman OR female AND obesity AND bariatric AND surgery OR gastric AND bypass OR roux-en-y AND qualitative AND research)** | **Scopus** | **5/18** | **Removed vasomotor** | **7 qualitative but irrelevant, 1 quantitative, 5 duplicate, 5 relevant** |
| **Women, Fertility, Bariatric surgery, Sexual function, Obesity, Sex hormones, Experience, Hermeneutics, Qualitative research, Vasomotor (Mesh and S-search tool)** | **Cochrane** | **0/0** |  |  |
| **Women, Fertility, Bariatric surgery, Sexual function, Obesity, Sex hormones, Experience, Hermeneutics, Qualitative research (Mesh and S-search tool)** | **Cochrane** | **0/0** | **Remove vasomotor** |  |
| **Women, Fertility, Bariatric surgery, Sexual function, Obesity, Sex hormones, Experience, Qualitative research (Mesh and S-search tool)** | **Cochrane** | **0/0** | **Remove hermeneutics** |  |
| **Women, Fertility, Bariatric surgery, Sexual function, Obesity, Sex hormones, Qualitative research (Mesh and S-search tool)** | **Cochrane** | **0/0** | **Removed experience** |  |
| **Women, Bariatric surgery, Sexual function, Obesity, Sex hormones, Qualitative research (Mesh and S-search tool)** | **Cochrane** | **0/0** | **Removed fertility** |  |
| **Women, Bariatric surgery, Obesity, Sex hormones, Qualitative research (Mesh and S-search tool)** | **Cochrane** | **0/0** | **Removed sexual function** |  |
| **Women, Bariatric surgery, Obesity, Sex hormones, Experiences (Mesh and S-search tool)** | **Cochrane** | **0/0** | **Removed qualitative research, added experience** |  |
| **Women, Bariatric surgery, Obesity, Sexual function, Experiences (Mesh and S-search tool)** | **Cochrane** | **0/0** | **Removed sex hormones, added sexual function** |  |
| **Women, Bariatric surgery, Obesity, Fertility, Experiences (Mesh and S-search tool)** | **Cochrane** | **0/0** | **Removed sexual function, added fertility** |  |
| **Women, Bariatric surgery, Obesity, Vasomotor, Experiences (Mesh and S-search tool)** | **Cochrane** | **0/0** | **Removed fertility, added vasomotor** |  |
| **Women, Bariatric surgery, Obesity, Fertility, Qualitative research (Mesh and S-search tool)** | **Cochrane** | **0/0** | **Removed experience and vasomotor, add qualitative research and fertility** |  |
| **Women, Bariatric surgery, Obesity, Sexual function, Qualitative research (Mesh and S-search tool)** | **Cochrane** | **0/0** | **Remove fertility, added sexual function** |  |
| **Women, Bariatric surgery, Obesity, Vasomotor, Qualitative research (Mesh and S-search tool)** | **Cochrane** | **0/0** | **Removed sexual function, added vasomotor** |  |
| **Women, Bariatric surgery, Obesity, Qualitative research (Mesh and S-search tool)** | **Cochrane** | **0/0** | **Removed vasomotor** |  |
| **Women, Bariatric surgery, Obesity, Qualitative research (Mesh and S-search tool)** | **Cochrane** | **0/1** | **Scaled back using search manager** | **1 quant** |
| **Women, Bariatric surgery, Obesity, Qualitative research (Mesh and S-search tool)** | **Cochrane** | **0/0** | **Terms using search manager** |  |
| **Women or Female or Woman or Females AND Fertility or Infertility or Reproductive or Fertile AND Bariatric surgery or Weight loss surgery or Gastric bypass surgery, or Y-en-Roux or Roux-en-Y AND Sexual function or Sexual dysfunction or Sexual life AND Obesity or Overweight or Fat or Obese or Unhealthy weight or High BMI AND Sex hormones or Estrogen AND Experiences or Perceptions or Attitudes or Views or Feelings or Qualitative or Perspective AND Hermeneutics AND Qualitative research or Qualitative study or Qualitative methods or Interview AND Vasomotor symptoms or Hot flushes or Hot flashes or Night sweat** | **WebofScience**  **(Clarivate)** | **0/0** |  |  |
| **Women or Female or Woman or Females AND Fertility or Infertility or Reproductive or Fertile AND Bariatric surgery or Weight loss surgery or Gastric bypass surgery, or Y-en-Roux or Roux-en-Y AND Sexual function or Sexual dysfunction or Sexual life AND Obesity or Overweight or Fat or Obese or Unhealthy weight or High BMI AND Sex hormones or Estrogen AND Experiences or Perceptions or Attitudes or Views or Feelings or Qualitative or Perspective AND Hermeneutics AND Qualitative research or Qualitative study or Qualitative methods or Interview** | **WebofScience**  **(Clarivate)** | **0/0** | **Remove vasomotor** |  |
| **Women or Female or Woman or Females AND Fertility or Infertility or Reproductive or Fertile AND Bariatric surgery or Weight loss surgery or Gastric bypass surgery, or Y-en-Roux or Roux-en-Y AND Sexual function or Sexual dysfunction or Sexual life AND Obesity or Overweight or Fat or Obese or Unhealthy weight or High BMI AND Sex hormones or Estrogen AND Experiences or Perceptions or Attitudes or Views or Feelings or Qualitative or Perspective AND Qualitative research or Qualitative study or Qualitative methods or Interview** | **WebofScience**  **(Clarivate)** | **0/0** | **Removed hermeneutics** |  |
| **Women or Female or Woman or Females AND Fertility or Infertility or Reproductive or Fertile AND Bariatric surgery or Weight loss surgery or Gastric bypass surgery, or Y-en-Roux or Roux-en-Y AND Sexual function or Sexual dysfunction or Sexual life AND Obesity or Overweight or Fat or Obese or Unhealthy weight or High BMI AND Sex hormones or Estrogen AND Qualitative research or Qualitative study or Qualitative methods or Interview** | **WebofScience**  **(Clarivate)** | **0/1** | **Removed experience** | **1 study protocol** |
| **Women or Female or Woman or Females AND Bariatric surgery or Weight loss surgery or Gastric bypass surgery, or Y-en-Roux or Roux-en-Y AND Sexual function or Sexual dysfunction or Sexual life AND Obesity or Overweight or Fat or Obese or Unhealthy weight or High BMI AND Sex hormones or Estrogen AND Qualitative research or Qualitative study or Qualitative methods or Interview** | **WebofScience**  **(Clarivate)** | **0/1** | **Removed fertility** | **1 study protocol** |
| **Women or Female or Woman or Females AND Bariatric surgery or Weight loss surgery or Gastric bypass surgery, or Y-en-Roux or Roux-en-Y AND Obesity or Overweight or Fat or Obese or Unhealthy weight or High BMI AND Sex hormones or Estrogen AND Qualitative research or Qualitative study or Qualitative methods or Interview** | **WebofScience**  **(Clarivate)** | **0/3** | **Removed sexual function** | **1 duplicate, 1 study protocol, 1 quantitative** |
| **Women or Female or Woman or Females AND Bariatric surgery or Weight loss surgery or Gastric bypass surgery, or Y-en-Roux or Roux-en-Y AND Obesity or Overweight or Fat or Obese or Unhealthy weight or High BMI AND Sex hormones or Estrogen AND Experiences or Perceptions or Attitudes or Views or Feelings or Qualitative or Perspective** | **WebofScience**  **(Clarivate)** | **0/17** | **Removed qualitative research, added experience** | **13 quantitative, 3 reviews, 1 duplicate** |
| **Women or Female or Woman or Females AND Bariatric surgery or Weight loss surgery or Gastric bypass surgery, or Y-en-Roux or Roux-en-Y AND Obesity or Overweight or Fat or Obese or Unhealthy weight or High BMI AND Experiences or Perceptions or Attitudes or Views or Feelings or Qualitative or Perspective AND Sexual function or Sexual dysfunction or Sexual life** | **WebofScience**  **(Clarivate)** | **0/24** | **Removed sex hormones, added sexual function** | **17 quantitative, 1 review, 5 duplicates, 1 case report** |
| **Women or Female or Woman or Females AND Bariatric surgery or Weight loss surgery or Gastric bypass surgery, or Y-en-Roux or Roux-en-Y AND Obesity or Overweight or Fat or Obese or Unhealthy weight or High BMI AND Experiences or Perceptions or Attitudes or Views or Feelings or Qualitative or Perspective AND Fertility or Infertility or Reproductive or Fertile** | **WebofScience**  **(Clarivate)** | **0/27** | **Removed sexual function, add fertility** | **10 quantitative, 11 reviews, 2 duplicates, 3 case reports, 1 qualitative but irrelevant** |
| **Women or Female or Woman or Females AND Bariatric surgery or Weight loss surgery or Gastric bypass surgery, or Y-en-Roux or Roux-en-Y AND Obesity or Overweight or Fat or Obese or Unhealthy weight or High BMI AND Experiences or Perceptions or Attitudes or Views or Feelings or Qualitative or Perspective AND Vasomotor symptoms or Hot flushes or Hot flashes or Night sweat** | **WebofScience**  **(Clarivate)** | **0/1** | **Removed fertility, added vasomotor** | **1 case report** |
| **Women or Female or Woman or Females AND Bariatric surgery or Weight loss surgery or Gastric bypass surgery, or Y-en-Roux or Roux-en-Y AND Obesity or Overweight or Fat or Obese or Unhealthy weight or High BMI AND Fertility or Infertility or Reproductive or Fertile AND Qualitative research or Qualitative study or Qualitative methods or Interview** | **WebofScience**  **(Clarivate)** | **0/15** | **Removed experience and vasomotor, added qualitative research and fertility** | **5 quantitative, 3 qualitative but irrelevant, 1 protocol, 3 reviews, 3 duplicates** |
| **Women or Female or Woman or Females AND Bariatric surgery or Weight loss surgery or Gastric bypass surgery, or Y-en-Roux or Roux-en-Y AND Obesity or Overweight or Fat or Obese or Unhealthy weight or High BMI AND Qualitative research or Qualitative study or Qualitative methods or Interview AND Sexual function or Sexual dysfunction or Sexual life** | **WebofScience**  **(Clarivate)** | **0/14** | **Removed fertility, added sexual function** | **4 quantitative, 2 case studies, 6 duplicates, 1 qualitative but irrelevant, 1 study protocol** |
| **Women or Female or Woman or Females AND Bariatric surgery or Weight loss surgery or Gastric bypass surgery, or Y-en-Roux or Roux-en-Y AND Obesity or Overweight or Fat or Obese or Unhealthy weight or High BMI AND Qualitative research or Qualitative study or Qualitative methods or Interview AND Vasomotor symptoms or Hot flushes or Hot flashes or Night sweat** | **WebofScience**  **(Clarivate)** | **0/0** | **Removed sexual function, added vasomotor** |  |
| **Women or Female or Woman or Females AND Bariatric surgery or Weight loss surgery or Gastric bypass surgery, or Y-en-Roux or Roux-en-Y AND Obesity or Overweight or Fat or Obese or Unhealthy weight or High BMI AND Qualitative research or Qualitative study or Qualitative methods or Interview** | **WebofScience**  **(Clarivate)** | **0/364** | **Removed vasomotor** | **High yield, add limits** |
| **Women or Female or Woman or Females AND Bariatric surgery or Weight loss surgery or Gastric bypass surgery, or Y-en-Roux or Roux-en-Y AND Obesity or Overweight or Fat or Obese or Unhealthy weight or High BMI AND Qualitative research or Qualitative study or Qualitative methods or Interview** | **WebofScience**  **(Clarivate)** | **3/114** | **Added limits: female, year 00-22, articles** | **68 quantitative, 1 review, 10 duplicates, 30 qualitative but irrelevant, 1 case study, 1 protocol, 3 relevant** |
| **Women or Female or Woman or Females AND Fertility or Infertility or Reproductive or Fertile AND Bariatric surgery or Weight loss surgery or Gastric bypass surgery, or Y-en-Roux or Roux-en-Y AND Sexual function or Sexual dysfunction or Sexual life AND Obesity or Overweight or Fat or Obese or Unhealthy weight or High BMI AND Sex hormones or Estrogen AND Experiences or Perceptions or Attitudes or Views or Feelings or Qualitative or Perspective AND Hermeneutics AND Qualitative research or Qualitative study or Qualitative methods or Interview AND Vasomotor symptoms or Hot flushes or Hot flashes or Night sweat** | **OpenGrey** | **0/0** |  |  |
| **Women or Female or Woman or Females AND Fertility or Infertility or Reproductive or Fertile AND Bariatric surgery or Weight loss surgery or Gastric bypass surgery, or Y-en-Roux or Roux-en-Y AND Sexual function or Sexual dysfunction or Sexual life AND Obesity or Overweight or Fat or Obese or Unhealthy weight or High BMI AND Sex hormones or Estrogen AND Experiences or Perceptions or Attitudes or Views or Feelings or Qualitative or Perspective AND Hermeneutics AND Qualitative research or Qualitative study or Qualitative methods or Interview** | **OpenGrey** | **0/0** | **Removed vasomotor** |  |
| **Women or Female or Woman or Females AND Fertility or Infertility or Reproductive or Fertile AND Bariatric surgery or Weight loss surgery or Gastric bypass surgery, or Y-en-Roux or Roux-en-Y AND Sexual function or Sexual dysfunction or Sexual life AND Obesity or Overweight or Fat or Obese or Unhealthy weight or High BMI AND Sex hormones or Estrogen AND Experiences or Perceptions or Attitudes or Views or Feelings or Qualitative or Perspective AND Qualitative research or Qualitative study or Qualitative methods or Interview** | **OpenGrey** | **0/0** | **Removed hermeneutics** |  |
| **Women or Female or Woman or Females AND Fertility or Infertility or Reproductive or Fertile AND Bariatric surgery or Weight loss surgery or Gastric bypass surgery, or Y-en-Roux or Roux-en-Y AND Sexual function or Sexual dysfunction or Sexual life AND Obesity or Overweight or Fat or Obese or Unhealthy weight or High BMI AND Sex hormones or Estrogen AND Qualitative research or Qualitative study or Qualitative methods or Interview** | **OpenGrey** | **0/0** | **Removed experience** |  |
| **Women or Female or Woman or Females AND Bariatric surgery or Weight loss surgery or Gastric bypass surgery, or Y-en-Roux or Roux-en-Y AND Sexual function or Sexual dysfunction or Sexual life AND Obesity or Overweight or Fat or Obese or Unhealthy weight or High BMI AND Sex hormones or Estrogen AND Qualitative research or Qualitative study or Qualitative methods or Interview** | **OpenGrey** | **0/0** | **Removed fertility** |  |
| **Women or Female or Woman or Females AND Bariatric surgery or Weight loss surgery or Gastric bypass surgery, or Y-en-Roux or Roux-en-Y AND Obesity or Overweight or Fat or Obese or Unhealthy weight or High BMI AND Sex hormones or Estrogen AND Qualitative research or Qualitative study or Qualitative methods or Interview** | **OpenGrey** | **0/0** | **Removed sexual function** |  |
| **Women or Female or Woman or Females AND Bariatric surgery or Weight loss surgery or Gastric bypass surgery, or Y-en-Roux or Roux-en-Y AND Obesity or Overweight or Fat or Obese or Unhealthy weight or High BMI AND Sex hormones or Estrogen AND Experiences or Perceptions or Attitudes or Views or Feelings or Qualitative or Perspective** | **OpenGrey** | **0/0** | **Removed qualitative research, added experience** |  |
| **Women or Female or Woman or Females AND Bariatric surgery or Weight loss surgery or Gastric bypass surgery, or Y-en-Roux or Roux-en-Y AND Obesity or Overweight or Fat or Obese or Unhealthy weight or High BMI AND Experiences or Perceptions or Attitudes or Views or Feelings or Qualitative or Perspective AND Sexual function or Sexual dysfunction or Sexual life** | **OpenGrey** | **0/0** | **Removed sex hormones, added sexual function** |  |
| **Women or Female or Woman or Females AND Bariatric surgery or Weight loss surgery or Gastric bypass surgery, or Y-en-Roux or Roux-en-Y AND Obesity or Overweight or Fat or Obese or Unhealthy weight or High BMI AND Experiences or Perceptions or Attitudes or Views or Feelings or Qualitative or Perspective AND Fertility or Infertility or Reproductive or Fertile** | **OpenGrey** | **0/0** | **Removed sexual function, added fertility** |  |
| **Women or Female or Woman or Females AND Bariatric surgery or Weight loss surgery or Gastric bypass surgery, or Y-en-Roux or Roux-en-Y AND Obesity or Overweight or Fat or Obese or Unhealthy weight or High BMI AND Experiences or Perceptions or Attitudes or Views or Feelings or Qualitative or Perspective AND Vasomotor symptoms or Hot flushes or Hot flashes or Night sweat** | **OpenGrey** | **0/0** | **Removed fertility, added vasomotor** |  |
| **Women or Female or Woman or Females AND Bariatric surgery or Weight loss surgery or Gastric bypass surgery, or Y-en-Roux or Roux-en-Y AND Obesity or Overweight or Fat or Obese or Unhealthy weight or High BMI AND Fertility or Infertility or Reproductive or Fertile AND Qualitative research or Qualitative study or Qualitative methods or Interview** | **OpenGrey** | **0/0** | **Removed experience and vasomotor, added qualitative research and fertility** |  |
| **Women or Female or Woman or Females AND Bariatric surgery or Weight loss surgery or Gastric bypass surgery, or Y-en-Roux or Roux-en-Y AND Obesity or Overweight or Fat or Obese or Unhealthy weight or High BMI AND Qualitative research or Qualitative study or Qualitative methods or Interview AND Sexual function or Sexual dysfunction or Sexual life** | **OpenGrey** | **0/0** | **Removed fertility, added sexual function** |  |
| **Women or Female or Woman or Females AND Bariatric surgery or Weight loss surgery or Gastric bypass surgery, or Y-en-Roux or Roux-en-Y AND Obesity or Overweight or Fat or Obese or Unhealthy weight or High BMI AND Qualitative research or Qualitative study or Qualitative methods or Interview AND Vasomotor symptoms or Hot flushes or Hot flashes or Night sweat** | **OpenGrey** | **0/0** | **Removed sexual function, added vasomotor** |  |
| **Women or Female or Woman or Females AND Bariatric surgery or Weight loss surgery or Gastric bypass surgery, or Y-en-Roux or Roux-en-Y AND Obesity or Overweight or Fat or Obese or Unhealthy weight or High BMI AND Qualitative research or Qualitative study or Qualitative methods or Interview** | **OpenGrey** | **0/0** | **Removed vasomotor** |  |
