# Supplementary material for: Impact of bariatric surgery on premenopausal women’s womanliness: A qualitative systematic review and meta-synthesis
Source: PLoS One. 2024 Aug 29;19(8):e0308059. doi: 10.1371/journal.pone.0308059 (PMC11361607; doi:10.1371/journal.pone.0308059)
Supplement: S3 Table — (DOCX) [file pone.0308059.s003.docx]

Supporting information

S3: Excluded studies

**Table of Contents**

[Qualitative studies excluded by relevance](#_bookmark0) 72

[Qualitative studies excluded due to methodological limitations](#_bookmark1) 9

Qualitative studies excluded by relevance

| Reference | Reason for exclusion |
| --- | --- |
| Alegría CA, Larsen B. "That's who I am: a fat person in a thin body": weight loss, negative self-evaluation, and mitigating strategies following weight loss surgery. J Am Assoc Nurse Pract. 2015 Mar;27(3):137-44. doi: 10.1002/2327-6924.12158. Epub 2014 Jul 25. PMID: 25066580. | Population not relevant |
| Alqout O, Reynolds F. Experiences of obesity among Saudi Arabian women contemplating bariatric surgery: an interpretative phenomenological analysis. J Health Psychol. 2014 May;19(5):664-77. doi: 10.1177/1359105313476977. Epub 2013 Mar 11. PMID: 23479306. | Setting not relevant |
| Amiri F, Ramezani Tehrani F, Simbar M, Mohammadpour Thamtan RA, Shiva N. Female Gender Scheme is Disturbed by Polycystic Ovary Syndrome: A Qualitative Study From Iran. Iran Red Crescent Med J. 2014 Feb;16(2):e12423. doi: 10.5812/ircmj.12423. Epub 2014 Feb 7. PMID: 24719724; PMCID: PMC3965857. | Outcomes not relevant |
| Billing-Bullen G, Nielsen D, Wham C, Kruger R. Enablers and barriers to prevent weight-regain post bariatric surgery - A qualitative enquiry. Eat Behav. 2022 Dec;47:101677. doi: 10.1016/j.eatbeh.2022.101677. Epub 2022 Oct 6. PMID: 36252389. | Perspective not relevant |
| Couch, L. Y. (2018). A phenomenological examination of the lived experience for women after bariatric surgery for morbid obesity: Implications for counseling [ProQuest Information & Learning]. In Dissertation Abstracts International: Section B: The Sciences and Engineering (Vol. 79, Issue 10–B(E)). | Methodological limitations |
| Coulman KD, MacKichan F, Blazeby JM, Donovan JL, Owen-Smith A. Patients' experiences of life after bariatric surgery and follow-up care: a qualitative study. BMJ Open. 2020 Feb 6;10(2):e035013. doi: 10.1136/bmjopen-2019-035013. PMID: 32034030; PMCID: PMC7045271. | Population and setting not relevant |
| Dahlberg K, Bylund A, Stenberg E, Jaensson M. An endeavour for change and self-efficacy in transition: patient perspectives on postoperative recovery after bariatric surgery-a qualitative study. Int J Qual Stud Health Well-being. 2022 Dec;17(1):2050458. doi: 10.1080/17482631.2022.2050458. PMID: 35291912; PMCID: PMC8933016. | Population not relevant |
| da Silva SS, da Costa Maia A. Obesity and treatment meanings in bariatric surgery candidates: a qualitative study. Obes Surg. 2012 Nov;22(11):1714-22. doi: 10.1007/s11695-012-0716-y. PMID: 22820955. | Population not relevant |
| de Oliveira, D. M., Barbosa Merighi, M. A., Kortchmar, E., Augusta Braga, V., Henrique da Silva, M., & Pinto de Jesus, M. C. (2016). Experience of women in the postoperative period of bariatric surgery: a phenomenological study. Online Brazilian Journal of Nursing, 15(1), 1–10. | Population not relevant |
| de Oliveira, D. M., Barbosa Merighi, M. A., & Pinto de Jesus, M. C. (2014). The decision of an obese woman to have bariatric surgery: the social phenomenology. Revista Da Escola de Enfermagem Da USP, 48(6), 970–976. https://doi-org.e.bibl.liu.se/10.1590/S0080-623420140000700002 | Population and perspective not relevant |
| Engström, M., & Forsberg, A. (2011). Wishing for deburdening through a sustainable control after bariatric surgery. International Journal of Qualitative Studies on Health & Well-Being, 6(1), 1–13. https://doi-org.e.bibl.liu.se/10.3402/qhw.v6i1.5901 | Population not relevant |
| Faria-Schützer DB, Surita FG, Alves VL, Vieira CM, Turato ER. Emotional Experiences of Obese Women with Adequate Gestational Weight Variation: A Qualitative Study. PLoS One. 2015 Nov 3;10(11):e0141879. doi: 10.1371/journal.pone.0141879. PMID: 26529600; PMCID: PMC4631528. | Setting and outcome not relevant |
| Followell, J. (2009). Experiences of women before and after bariatric surgery [ProQuest Information & Learning]. In Dissertation Abstracts International Section A: Humanities and Social Sciences (Vol. 69, Issue 8–A, p. 3047). | Methodological limitations |
| Forsberg A, Engström A, Söderberg S. From reaching the end of the road to a new lighter life - people's experiences of undergoing gastric bypass surgery. Intensive Crit Care Nurs. 2014 Apr;30(2):93-100. doi: 10.1016/j.iccn.2013.08.006. Epub 2013 Sep 24. PMID: 24074545. | Population not relevant |
| Geraci, A. A., Brunt, A. R., & Marihart, C. L. (2014). Social support systems: A qualitative analysis of female bariatric patients after the first two years postoperative. Bariatric Surgical Practice and Patient Care, 9(2), 66-71. doi:10.1089/bari.2014.0004 | Population not relevant |
| Gilmartin J. Body image concerns amongst massive weight loss patients. J Clin Nurs. 2013 May;22(9-10):1299-309. doi: 10.1111/jocn.12031. PMID: 23574293. | Population not relevant |
| Griauzde DH, Ibrahim AM, Fisher N, Stricklen A, Ross R, Ghaferi AA. Understanding the psychosocial impact of weight loss following bariatric surgery: a qualitative study. BMC Obes. 2018 Dec 3;5:38. doi: 10.1186/s40608-018-0215-3. PMID: 30524743; PMCID: PMC6276134. | Population not relevant |
| Granero-Molina J, Torrente-Sánchez MJ, Ferrer-Márquez M, Hernández-Padilla JM, Sánchez-Navarro M, Ruiz-Muelle A, Ruiz-Fernández MD, Fernández-Sola C. Sexuality amongst heterosexual women with morbid obesity in a bariatric surgery programme: A qualitative study. J Adv Nurs. 2021 Nov;77(11):4537-4548. doi: 10.1111/jan.14972. Epub 2021 Jul 12. PMID: 34252209. | Population not relevant |
| Groller KD, Teel C, Stegenga KH, El Chaar M. Patient perspectives about bariatric surgery unveil experiences, education, satisfaction, and recommendations for improvement. Surg Obes Relat Dis. 2018 Jun;14(6):785-796. doi: 10.1016/j.soard.2018.02.016. Epub 2018 Feb 17. PMID: 29703505. | Population not relevant |
| Groven KS, Råheim M, Engelsrud G. Changing bodies, changing habits: women's experiences of interval training following gastric bypass surgery. Health Care Women Int. 2015;36(3):276-302. doi: 10.1080/07399332.2013.794465. Epub 2013 Jul 18. PMID: 23865845. | Population and perspective not relevant |
| Groven KS. "They think surgery is just a quick fix". Int J Qual Stud Health Well-being. 2014 Jul 11;9:24378. doi: 10.3402/qhw.v9.24378. Erratum in: Int J Qual Stud Health Well-being. 2014 Jan;9(1):25297. PMID: 25022267; PMCID: PMC4095757. | Population not relevant |
| Groven KS, Engelsrud G. Negotiating options in weight-loss surgery : "Actually I didn't have any other option". Med Health Care Philos. 2016 Sep;19(3):361-70. doi: 10.1007/s11019-015-9677-y. PMID: 26715285. | Population not relevant |
| Groven KS. "Then I Can Become Very Ill": Women's Experiences of Living With Irreversible Changes in Their Viscera. Health Care Women Int. 2016 Jun;37(6):599-619. doi: 10.1080/07399332.2014.942906. Epub 2014 Oct 2. PMID: 25101876. | Population and perspective not relevant |
| Groven KS, Glenn NM. The experience of regaining weight following weight loss surgery: A narrative-phenomenological exploration. Health Care Women Int. 2016 Nov;37(11):1185-1202. doi: 10.1080/07399332.2016.1195386. Epub 2016 Jun 1. PMID: 27249448. | Methodological limitations |
| Groven KS, Råheim M, Engelsrud G. Dis-appearance and dys-appearance anew: living with excess skin and intestinal changes following weight loss surgery. Med Health Care Philos. 2013 Aug;16(3):507-23. doi: 10.1007/s11019-012-9397-5. PMID: 22395970. | Population not relevant |
| Groven KS, Råheim M, Braithwaite J, Engelsrud G. Weight loss surgery as a tool for changing lifestyle? Med Health Care Philos. 2013 Nov;16(4):699-708. doi: 10.1007/s11019-013-9471-7. PMID: 23471482. | Population not relevant |
| Groven KS, Råheim M, Engelsrud G. "My quality of life is worse compared to my earlier life": Living with chronic problems after weight loss surgery. Int J Qual Stud Health Well-being. 2010 Nov 18;5(4). doi: 10.3402/qhw.v5i4.5553. PMID: 21103070; PMCID: PMC2989899. | Population and perspective not relevant |
| Hayden MJ, Dixon ME, Dixon JB, Playfair J, O'Brien PE. Perceived discrimination and stigmatisation against severely obese women: age and weight loss make a difference. Obes Facts. 2010 Feb;3(1):7-14. doi: 10.1159/000273206. Epub 2010 Feb 11. PMID: 20215790; PMCID: PMC6452106. | Population not relevant |
| Heidmann, J., & Grønkjær, M. (2015). Health-related quality of life six years after gastric bypass: A mixed methods study. Bariatric Surgical Practice and Patient Care, 10(2), 56–61. https://doi-org.e.bibl.liu.se/10.1089/bari.2014.0052 | Population not relevant |
| Homer CV, Tod AM, Thompson AR, Allmark P, Goyder E. Expectations and patients' experiences of obesity prior to bariatric surgery: a qualitative study. BMJ Open. 2016 Feb 8;6(2):e009389. doi: 10.1136/bmjopen-2015-009389. PMID: 26857104; PMCID: PMC4746450. | Population and perspective not relevant |
| Irwin JD Liu RH. Understanding the post-surgical bariatric experiences of patients two or more years after surgery. Qual Life Res. 2017 Nov;26(11):3157-3168. doi: 10.1007/s11136-017-1652-z. Epub 2017 Jul 13. PMID: 28707046. | Population not relevant |
| Jones L, Cleator J, Yorke J. Maintaining weight loss after bariatric surgery: when the spectator role is no longer enough. Clin Obes. 2016 Aug;6(4):249-58. doi: 10.1111/cob.12152. Epub 2016 Jun 8. PMID: 27273813. | Population not relevant |
| Jose K., Venn A., Sharman M., Wilkinson S., Williams D., Ezzy D. Understanding gender differences in bariatric surgery: Moving beyond traditional representation and appearance concerns. Obesity Reviews 2016 17 SUPPL. 2 (58). | Setting and population not relevant |
| Järvholm K, Olbers T, Engström M. Patients' views of long-term results of bariatric surgery for super-obesity: sustained effects, but continuing struggles. Surg Obes Relat Dis. 2021 Jun;17(6):1152-1164. doi: 10.1016/j.soard.2021.02.024. Epub 2021 Mar 2. PMID: 33785271. | Population not relevant |
| Kabu Hergül F, Özbayır T. I Am As Normal As Everyone Now. . . : Examination of Experiences of Patients Undergoing Bariatric Surgery According to Roy's Adaptation Model: A Qualitative Study. Clin Nurs Res. 2021 May;30(4):511-521. doi: 10.1177/1054773819880291. Epub 2019 Oct 14. PMID: 31609134. | Population not relevant |
| Keish, J. F. (2005). A phenomenological analysis of the lived experiences of adult females undergoing rapid weight loss due to Roux-en-Y, weight loss surgery [ProQuest Information & Learning]. In Dissertation Abstracts International: Section B: The Sciences and Engineering (Vol. 66, Issue 3–B, p. 1722). | Methodological limitations |
| Knutsen, I. R., Terragni, L., & Foss, C. (2013). Empowerment and bariatric surgery: Negotiations of credibility and control. Qualitative Health Research, 23(1), 66–77. https://doi-org.e.bibl.liu.se/10.1177/1049732312465966 | Population not relevant |
| LePage, C. T. (2010). The lived experience of individuals following Roux-en-Y gastric bypass surgery: A phenomenological study. Bariatric Nursing and Surgical Patient Care, 5(1), 57–64. https://doi-org.e.bibl.liu.se/10.1089/bar.2009.9938 | Population not relevant |
| Lier, H. Ø., Aastrom, S., & Rørtveit, K. (2016). Patients’ daily life experiences five years after gastric bypass surgery—A qualitative study. Journal of Clinical Nursing, 25(3–4), 322–331. https://doi-org.e.bibl.liu.se/10.1111/jocn.13049 | Population not relevant |
| Lindberg, S., Wennström, B., & Larsson, A. (2021). Facing an unexpected reality – oscillating between health and suffering 4–6 years after bariatric surgery. Scandinavian Journal of Caring Sciences. https://doi-org.e.bibl.liu.se/10.1111/scs.12999 | Population not relevant |
| Maxwell, M.J. An interpretative phenomenological analysis investigating UK female experiences of psychosocial adjustment following bariatric surgery. (2019). Qualitative Report, 24 (7), art. no. 13, pp. 1714-1730. | Population not relevant |
| Natvik E, Råheim M, Sviland R. The hamster wheel: a case study on embodied narrative identity and overcoming severe obesity. Med Health Care Philos. 2021 Jun;24(2):255-267. doi: 10.1007/s11019-021-10002-x. Epub 2021 Jan 13. PMID: 33439383; PMCID: PMC8128800. | Population not relevant |
| Natvik E, Råheim M, Andersen JR, Moltu C. Living a successful weight loss after severe obesity. Int J Qual Stud Health Well-being. 2018 Dec;13(1):1487762. doi: 10.1080/17482631.2018.1487762. PMID: 29947301; PMCID: PMC6022235. | Population not relevant |
| Natvik E, Gjengedal E, Råheim M. Totally changed, yet still the same: patients' lived experiences 5 years beyond bariatric surgery. Qual Health Res. 2013 Sep;23(9):1202-14. doi: 10.1177/1049732313501888. Epub 2013 Aug 6. PMID: 23921810. | Population not relevant |
| Newhook JT, Gregory D, Twells L. 'Fat girls' and 'big guys': gendered meanings of weight loss surgery. Sociol Health Illn. 2015 Jun;37(5):653-67. doi: 10.1111/1467-9566.12219. Epub 2015 Feb 11. PMID: 25677753. | Population not relevant |
| O Perdue, T. (2017). Psychological Adjustment to Surgically Induced Weight Loss: Distinguishing Factors in Bariatric Patients. Psychological Adjustment to Surgically Induced Weight Loss: Distinguishing Factors in Bariatric Patients, 1. | Population not relevant |
| Ogden J, Clementi C, Aylwin S. The impact of obesity surgery and the paradox of control: A qualitative study. Psychol Health. 2006;21(2):273-93. doi: 10.1080/14768320500129064. PMID: 21985121. | Population not relevant |
| Ogden J, Clementi C, Aylwin S, Patel A. Exploring the impact of obesity surgery on patients' health status: a quantitative and qualitative study. Obes Surg. 2005 Feb;15(2):266-72. doi: 10.1381/0960892053268291. PMID: 15802072. | Population and setting not relevant |
| Ogle JP, Park J, Damhorst ML, Bradley LA. Social Support for Women Who Have Undergone Bariatric Surgery. Qual Health Res. 2016 Jan;26(2):176-93. doi: 10.1177/1049732315570132. Epub 2015 Feb 6. PMID: 25662946. | Population not relevant |
| Park J. Self-determination and motivation for bariatric surgery: a qualitative study. Psychol Health Med. 2016 Oct;21(7):800-5. doi: 10.1080/13548506.2015.1131996. Epub 2015 Dec 28. PMID: 26708344. | Population not relevant |
| Park, J. (2015). The meanings of physical appearance in patients seeking bariatric surgery. Health Sociology Review, 24(3), 242–255. https://doi-org.e.bibl.liu.se/10.1080/14461242.2015.1051080 | Population not relevant |
| Perdue, T. O., Schreier, A., Swanson, M., Neil, J., & Carels, R. (2018). Evolving self view and body image concerns in female postoperative bariatric surgery patients. Journal of Clinical Nursing (John Wiley & Sons, Inc.), 27(21–22), 4018–4027. https://doi-org.e.bibl.liu.se/10.1111/jocn.14527 | Population not relevant |
| Pories ML, Hodgson J, Rose MA, Pender J, Sira N, Swanson M. Following Bariatric Surgery: an Exploration of the Couples' Experience. Obes Surg. 2016 Jan;26(1):54-60. doi: 10.1007/s11695-015-1720-9. PMID: 26024736. | Population and perspective not relevant |
| Possmark S, Berglind D, Sellberg F, Ghaderi A, Persson M. To be or not to be active - a matter of attitudes and social support? Women's perceptions of physical activity five years after Roux-en-Y Gastric Bypass surgery. Int J Qual Stud Health Well-being. 2019 Dec;14(1):1612704. doi: 10.1080/17482631.2019.1612704. PMID: 31072238; PMCID: PMC6522969. | Population and perspective not relevant |
| Rahiri JL, Tuhoe J, MacCormick AD, Hill AG, Harwood M. Exploring motivation for bariatric surgery among Indigenous Māori women. Obes Res Clin Pract. 2019 Sep-Oct;13(5):486-491. doi: 10.1016/j.orcp.2019.09.004. Epub 2019 Oct 5. PMID: 31591083. | Perspective not relevant |
| Recore, N. (2012). A qualitative exploration of women’s perceived dating eligibility and dating experiences before and after weight loss surgery [ProQuest Information & Learning]. In Dissertation Abstracts International: Section B: The Sciences and Engineering (Vol. 73, Issue 4–B, p. 2515). | Methodological limitations |
| Riggs, D. A. (2006). Women’s lived experience and meaning of bariatric surgery [ProQuest Information & Learning]. In Dissertation Abstracts International: Section B: The Sciences and Engineering (Vol. 66, Issue 11–B, p. 5894). | Population and perspective not relevant |
| Sharman, M., Hensher, M., Wilkinson, S., Williams, D., Palmer, A., Venn, A., & Ezzy, D. (2017). What are the support experiences and needs of patients who have received bariatric surgery? Health Expectations: An International Journal of Public Participation in Health Care & Health Policy, 20(1), 35–46. https://doi-org.e.bibl.liu.se/10.1111/hex.12423 | Population not relevant |
| Shawe, J., Cooke, D., Hart, K., McGowan, B. M., Pring, C., Subramanian, D., & Whyte, M. (2014). Pregnancy after diabetes obesity surgery (PADOS): Qualitative study of pre-pregnancy care. Pregnancy Hypertension, 4(3), 238. https://doi-org.e.bibl.liu.se/10.1016/j.preghy.2014.03.027 | Perspective not relevant |
| Sutton DH, Murphy N, & Raines DA. (2009). Transformation: the “life-changing” experience of women who undergo a surgical weight loss intervention. Bariatric Nursing & Surgical Patient Care, 4(4), 299–306. https://doi-org.e.bibl.liu.se/10.1089/bar.2009.9948 | Population not relevant |
| Sweet, L., Vasilevski, V., Angel, M. G., Mathison, M. A., & Teale, G. (2022). O69 - Experiences and information needs of women who become pregnant after bariatric surgery...Australian College of Midwives National Conference - Together at the Top, September 13-15, 2022, Cairns, Queensland, Australia. Women & Birth, 35, N.PAG. https://doi-org.e.bibl.liu.se/10.1016/j.wombi.2022.07.075 | Methodological limitations |
| Thies-Lagergren L, Mårtensson A, Safi A. Women's experiences of pregnancy after gastric bypass surgery. Eur J Midwifery. 2022 Aug 4;6:52. doi: 10.18332/ejm/151550. PMID: 35974718; PMCID: PMC9348583. | Methodological limitations |
| Throsby K. "How could you let yourself get like that?" Stories of the origins of obesity in accounts of weight loss surgery. Soc Sci Med. 2007 Oct;65(8):1561-71. doi: 10.1016/j.socscimed.2007.06.005. Epub 2007 Jul 24. PMID: 17651875. | Population and perspective not relevant |
| Tolvanen L, Christenson A, Surkan PJ, Lagerros YT. Patients' Experiences of Weight Regain After Bariatric Surgery. Obes Surg. 2022 May;32(5):1498-1507. doi: 10.1007/s11695-022-05908-1. Epub 2022 Jan 21. PMID: 35061154; PMCID: PMC8986695. | Population not relevant |
| Trainer S, Benjamin T. Elective surgery to save my life: rethinking the "choice" in bariatric surgery. J Adv Nurs. 2017 Apr;73(4):894-904. doi: 10.1111/jan.13193. Epub 2016 Nov 23. PMID: 27779770. | Population not relevant |
| Van Vuuren JM, Strodl E, White KM, Lockie PD. Psychosocial presentation of female bariatric surgery patients after multiple revisional surgeries: A qualitative study. J Health Psychol. 2018 Sep;23(10):1261-1272. doi: 10.1177/1359105316648673. Epub 2016 Jun 7. PMID: 27270890. | Population and perspective not relevant |
| Warholm C, Marie Øien A, Råheim M. The ambivalence of losing weight after bariatric surgery. Int J Qual Stud Health Well-being. 2014 Jan 29;9:22876. doi: 10.3402/qhw.v9.22876. PMID: 24480033; PMCID: PMC3907679. | Methodological limitations |
| Watson C, Riazi A, Ratcliffe D. Exploring the Experiences of Women Who Develop Restrictive Eating Behaviours After Bariatric Surgery. Obes Surg. 2020 Jun;30(6):2131-2139. doi: 10.1007/s11695-020-04424-4. PMID: 32060849; PMCID: PMC7475057. | Population and perspective not relevant |
| Wright, C., Kelly, J. T., Healy, R., Musial, J., Campbell, K. L., & Hamilton, K. (2022). Lived experiences and unique psychosocial impacts following bariatric surgery in a publicly funded Australian tertiary hospital: A qualitative study. Australian Journal of Psychology, 74(1). https://doi-org.e.bibl.liu.se/10.1080/00049530.2022.2046445 | Population not relevant |
| Yin MXC, Leng LL, Liang Z, Chen XY, Chan CHY, Chan CLW. Objectification and ambiguity of body image in women with Polycystic Ovary Syndrome: A mixed-method study. J Affect Disord. 2022 Aug 1;310:296-303. doi: 10.1016/j.jad.2022.05.028. Epub 2022 May 11. PMID: 35561883. | Perspective not relevant |
| Zeller MH, Robson SM, Reiter-Purtill J, Kidwell KM, Kharofa RY, McCullough MB, Crosby LE, Howarth T, Comstock SE, Ley SL, Courcoulas AP, West-Smith L. Halo or horn? A qualitative study of mothers' experiences with feeding children during the first year following bariatric surgery. Appetite. 2019 Nov 1;142:104366. doi: 10.1016/j.appet.2019.104366. Epub 2019 Jul 10. PMID: 31301320. | Methodological limitations |
|  |  |
|  |  |
