# Supplementary material for: Impact of bariatric surgery on premenopausal women’s womanliness: A qualitative systematic review and meta-synthesis
Source: PLoS One. 2024 Aug 29;19(8):e0308059. doi: 10.1371/journal.pone.0308059 (PMC11361607; doi:10.1371/journal.pone.0308059)
Supplement: S4 Table — (DOCX) [file pone.0308059.s004.docx]

**Supporting information**

**S4: Primary key features of included qualitative articles.**

| **Author**  **Year**  **Country**  **Ref #** | **Alleva J M, et al.**  2023  The Netherlands  **32** |
| --- | --- |
| **Aim of study** | To improve body image among women who have undergone bariatric surgery |
| **Underpinning theory** | Body functionality |
| **Setting** | Dutch Obesity Clinic (NOK). |
| **Participants** | 103 women who had undergone bariatric surgery answered questionnaires and out of these 42 women wrote responses. They were aged between 24-67 years. Mean age: 46 |
| **Sampling method**  **Inclusion criteria** | Purposeful  Being female, 18–65 years old, having undergone bariatric surgery 5–7 months prior to the study. |
| **Data collection method**  **Interviewer** | Randomised study- intervention: questionnaires and written responses.  Not described |
| **Analysis methods** | Writings were analysed using Thematic Analysis (Braun and Clark). The research team was involved in the analysis process and discussed and validated the emergent themes. |
| **Analysts** | Psychologist/psychotherapist, Surgeon |
| **Measures to support trustworthiness** | Audit trail and interrater agreement |
| **Comments**  **Primary Research Appraisal Tool-Q (PRAT-Q)** | Methodological aspects are mostly fulfilled.  The qualitative part follows the method stated, and findings are clearly presented. |
| **Author**  **Year**  **Country**  **Ref #** | **Nilsson-Condori E et al.**  2019  Sweden  **33** |
| **Aim of study** | To explore the motives of women in fertile age for seeking bariatric surgery and their expectations of future fertility. |
| **Underpinning theory** | -- |
| **Setting** | A bariatric? centre in Malmö, Sweden. |
| **Participants** | 12 women, aged 20 to 35 years |
| **Sampling method**  **Inclusion criteria** | Purposeful  Women without children, aged 20 to 35 years, with the Swedish language and accepted for bariatric surgery. An obesity duration of >5 years and BMI >40 or BMI >35 with one or more comorbidity. |
| **Data collection method**  **Interviewer** | Semi-structured interviews with childless women. Length 38-95 minutes  Not described |
| **Analysis methods** | Recorded interviews were analysed using Thematic Analysis (Braun and Clarke).  The research team was involved in the analysis process and discussed and validated the emergent themes. |
| **Analysts** | A clinician at a fertility centre, a psychologist at another fertility centre and a nurse at the bariatric centre |
| **Measures to support trustworthiness** | Audit trail and statements were discussed |
| **Comments**  **Primary Research Appraisal Tool-Q (PRAT-Q)** | Methodological aspects mostly fulfilled.  The qualitative part follows the method stated, and findings are clearly presented. |
| **Author**  **Year**  **Country**  **Ref #** | **Nilsson-Condori E et al.**  2020  Sweden  **34** |
| **Aim of study** | To explore how women perceive the effects of bariatric surgery on quality of life, focusing on sexual health and fertility. |
| **Underpinning theory** | -- |
| **Setting** | A university-affiliated Swedish bariatric centre |
| **Participants** | 11 women, aged 20 to 34 years |
| **Sampling method**  **Inclusion criteria** | Purposeful  Women without children, aged 20 to 34 years, Swedish-speaking and accepted for bariatric surgery: an obesity duration of >5 years and BMI >40 or BMI >35 with one or more comorbidity. Follow up |
| **Data collection method**  **Interviewer** | Semi-structured interviews. Length 25-55 minutes.  A clinician at a fertility centre |
| **Analysis methods** | Recorded interviews were analysed using thematic Analysis (Braun and Clarke)  The research team was involved in the analysis process and discussed and validated the emergent themes |
| **Analysts** | A clinician at a fertility centre, a psychologist at another fertility centre and a nurse at the bariatric centre |
| **Measures to support trustworthiness** | Audit trail and statements were discussed |
| **Comments**  **Primary Research Appraisal Tool-Q (PRAT-Q)** | Methodological aspects are mostly fulfilled.  A qualitative study almost following the method stated, findings are clearly presented. |
| **Author**  **Year**  **Country**  **Ref #** | **Faccio E, et al.**  2016  Italy  **35** |
| **Aim of study** | To investigate obese patients’ beliefs and expectations before and one year after bariatric surgery. Changes and resistance to change in the identity system, the perception of the body, relationships and the quality of life was of particular interest. |
| **Underpinning theory** | The dialogical self-theory of Hermans |
| **Setting** | The Surgical Department of Obesity of St. Mary Angels Hospital of Pordenone,  Northern Italy |
| **Participants** | 15 (preop)+15 women(postop). Age >18, mean age 35. |
| **Sampling method**  **Inclusion criteria** | Purposive sampling  Diagnosis of obesity BMI > 40, more than 18 years old and started the process of having surgery. |
| **Data collection method**  **Interviewer** | A semi-structured interview  Not described |
| **Analysis methods** | Recorded interviews were analysed using discourse analysis. |
| **Analysts** | Psychologists, sociologists |
| **Measures to support trustworthiness** | Not described |
| **Comments**  **Primary Research Appraisal Tool-Q (PRAT-Q)** | Hard to follow the audit trail.  A qualitative study partly following the method stated, findings presented with many quotations. |
| **Author**  **Year**  **Country**  **Ref #** | **Guven B, et al.**  2021  Turkey  **36** |
| **Aim of study** | To explore the experiences of Turkish women with obesity in their sexual life before and after bariatric surgery |
| **Underpinning theory** | -- |
| **Setting** | A general surgery outpatient clinic of a training and research hospital where women were followed up after bariatric surgery. |
| **Participants** | 16 women, aged between 26-47 years |
| **Sampling method**  **Inclusion criteria** | Purposive sampling method was used to maximize the diversity.  Married women in heterosexual relationships who had undergone bariatric surgery. |
| **Data collection method**  **Interviewer** | Semi‐structured face‐to‐face interviews lasting between 45-60 minutes.  A female nurse with a PhD, trained in qualitative interviewing |
| **Analysis methods** | Data were analysed based on Colaizzi’s phenomenological data analysis method.  Independent analyses by individual researchers, followed by analyses and discussions within the research team. |
| **Analysts** | A nurse, a medical doctor and other |
| **Measures to support trustworthiness** | Trustworthiness, with its four components, was used. |
| **Comments**  **Primary Research Appraisal Tool-Q (PRAT-Q)** | Methodological aspects mostly fulfilled.  A qualitative study following the method stated, findings clearly presented. |
| **Author**  **Year**  **Country**  **Ref #** | **Jensen J F, et al.**  2013  Denmark  **37** |
| **Aim of study** | To understand the lived experience of body image in young women after obesity surgery. |
| **Underpinning theory** | -- |
| **Setting** | Denmark’s largest publicly funded obesity surgery department. |
| **Participants** | Five women aged 20 to 25 years |
| **Sampling method**  **Inclusion criteria** | Purposeful sampling  Being a young woman (18–25 years and had the surgical procedure performed within the last 12 months). |
| **Data collection method**  **Interviewer** | In-depth, semi-structured interviews lasted between 1 hour and 5 minutes to 1 hour and 42 minutes.  Not described |
| **Analysis methods** | Descriptive Phenomenology (Giorgi) inspiring Malterud |
| **Analysts** | Nursing Physiology, Occupational therapy, Psychology |
| **Measures to support trustworthiness** | Reflexivity, rigour, triangulation |
| **Comments**  **Primary Research Appraisal Tool-Q (PRAT-Q)** | Methodological aspects are mostly fulfilled.  A qualitative study almost following the method stated, findings are clearly presented. |
| **Author**  **Year**  **Country**  **Ref #** | **Magdaleno R, et al.**  2010  Brazil  **38** |
| **Aim of study** | To understand the meanings for women when undergoing bariatric surgery and, from a comprehensive–interpretative approach, examine what sustains therapeutic successes and failures. |
| **Underpinning theory** | A humanistic model |
| **Setting**  **Participants** | A tertiary public university hospital located in the city of Campinas, state of São Paulo  7 women aged between 28-49 years. |
| **Sampling method**  **Inclusion criteria** | Purposeful sampling  Women have had the bariatric surgery 1.5 to 3 years before the interview. |
| **Data collection method**  **Interviewer** | Semi-directed interviews with open-ended questions  Not described |
| **Analysis methods** | A form of content analysis |
| **Analysts** | Psychologist? Not clearly presented. |
| **Measures to support trustworthiness** | Hard to follow the audit trail.  No description of the action taken to reach trustworthiness |
| **Comments**  **Primary Research Appraisal Tool-Q (PRAT-Q)** | Methodological aspects are mostly fulfilled.  A qualitative study following the method stated, findings clearly presented. |
| **Author**  **Year**  **Country**  **Ref #** | **Magdaleno R,** **et al.** 2011 Brazil  **39** |
| **Aim of study** | To understand the gamut of meanings for women to undergo bariatric surgery, the impact that this represents in their lives, and the psychosocial complications. |
| **Underpinning theory** | Not described |
| **Setting** | A tertiary public university hospital located in the city of Campinas, state of São Paulo |
| **Participants** | 7 women, aged between 28-49 years. |
| **Sampling method**  **Inclusion criteria** | Purposeful sampling  Women have had the bariatric surgery |
| **Data collection method**  **Interviewer** | Semi-directed interviews with open-ended questions  Not described |
| **Analysis methods** | A form of content analysis. |
| **Analysts** | Psychologist? Not clearly presented. |
| **Measures to support trustworthiness** | Hard to follow the audit trail.  No description of the action taken to reach trustworthiness. |
| **Comments**  **Primary Research Appraisal Tool-Q (PRAT-Q)** | Methodological aspects not fulfilled.  A qualitative study following the method stated, findings clearly presented. |
| **Author**  **Year**  **Country**  **Ref #** | **Paul R,** **et al.** 2022 Sweden  **40** |
| **Aim of study** | To explore the expectations and experiences of women with a desire to have children as a motivation for undergoing bariatric surgery. |
| **Underpinning theory** | -- |
| **Setting** | A single surgical centre in Sweden. |
| **Participants** | 14 women, age range 22-37 years. |
| **Sampling method**  **Inclusion criteria** | Purposeful sampling Women aged between 18–45 years who had undergone laparoscopic gastric bypass surgery 2-5 years before the interview and stated a desire to become pregnant as a reason for surgery. |
| **Data collection method**  **Interviewer** | Semi-structured interviews in-depth interviews, (by telephone due to pandemic restrictions) lasted between 11-40 minutes.  Surgeon |
| **Analysis methods** | Recorded interviews were analysed using thematic analysis (Braun and Clarke)  The research team was involved in the analysis process and discussed and validated the emergent themes. |
| **Analysts** | Nurses, surgeons |
| **Measures to support trustworthiness** | Trustworthiness, with its four components, was used. |
| **Comments**  **Primary Research Appraisal Tool-Q (PRAT-Q)** | Methodological aspects fulfilled.  A qualitative study following the method stated, findings clearly presented. |
| **Author**  **Year**  **Country**  **Ref #** | **Young J & Burrows L**  2013 New Zealand  **41** |
| **Aim of study** | To examine what weight loss surgery offers as a mode of being in the world. |
| **Underpinning theory** | Foucauldian conceptualisations of subjectivity and discourse together with Merleau-Ponty’s work on embodiment |
| **Setting** | Video blogs |
| **Participants** | 2 women followed over 2 years. Age 35 and “20-something”. |
| **Sampling method**  **Inclusion criteria** | Purposive  Have had bariatric surgery |
| **Data collection method**  **Interviewer** | Video blogs on the internet  ---- |
| **Analysis methods** | Video blogs analysed with discourse analysis directed by theory |
| **Analysts** | Health Sciences, Sociology |
| **Measures to support trustworthiness** | Reflection about subjectivity |
| **Comments**  **Primary Research Appraisal Tool-Q (PRAT-Q)** | Methodological aspects fulfilled.  A qualitative study following the method stated, findings clearly presented. |
