# Supplementary material for: Impact of bariatric surgery on premenopausal women’s womanliness: A qualitative systematic review and meta-synthesis
Source: PLoS One. 2024 Aug 29;19(8):e0308059. doi: 10.1371/journal.pone.0308059 (PMC11361607; doi:10.1371/journal.pone.0308059)
Supplement: S1 Dataset — (DOCX) [file pone.0308059.s005.docx]

| **The horizon of the text** | **The horizon of the interpreter** | **The fusion** | **“Comment”** |
| --- | --- | --- | --- |
| **1 Alleva et al 2022** |  |  |  |
| Most women compared their current body to their presurgery body, even though the writing instructions did not mention making such comparisons.  Most comparisons were phrased positively (e.g., increased stamina) but some were phrased negatively (e.g., worsened taste perception). | Comparing body-pre and post-surgery  Most positive statements | Positive body image | **Comparing pre and post-surgery bodies**  **The positive experiences over weighted** |
| Overall, most women were positive about the effects of surgery, or described that the positive outcomes weighed more heavily. Almost all women described  enjoyment of bodily functions, such as experiencing pleasure and pride from physical activities. | The positive effects outweigh the negative  Improved body as a tool to enjoy life | Positive body image | **Positive body experiences- not only the look but also the function** |
| Further, most women described a positive body–self connection,  including the various ways their body communicates to them (e.g., developing headaches when stressed), how they respond to these signals (e.g.,  resting), and how their body enables them to express themselves and be independent. | Increased awareness of the body and its functions when the body no longer hinder or gives shame | Positive body image | **Positive body experiences- not only the look but also the function**  **No ashamed feelings** |
| Most women described the body as valuable in relation to important others, such as seeing one’s children grow up. It is noteworthy that this theme was  apparent in each of the three writing exercises, not just the writing exercise that focused on communication with others. | Maintaining a healthy body in order to live a long life | A healthy and functioning body | **Be healthy- live long and having a family** |
| Almost half of the women expressed appreciation for the resilience and reliability of their body. | Increased appreciation for the body when it is improved | Positive body image |  |
| Participant 50: “Because I can taste and smell, I  can enjoy food but also the smell of freshly  baked bread.” | A changed body creates a more positive outlook on life | Positive body image | **Positive body experiences- not only the look but also the function**  **No ashamed feelings** |
| Participant 56: “Nowadays I can easily swim 30  lengths without being tired or out of breath. This  gives me an incredible kick.” | The positive benefits of a healthy body give motivation | A healthy and functioning body |  |
| Participant 148: “I’ll get a migraine then, for  example [when being too busy]. That’s no fun,  but it is a function that works for me. I find it  difficult to respect my limits myself and this  migraine forces me to listen to my body.” | The body tells me when I need to calm down | A healthy and functioning body |  |
| Participant 101: “These functions are important  to me, because I would lead a very isolated life  without these functions, would be dependent on  others, and my life would become boring and  bleak.” | Life has no value if there is no independence | A healthy and functioning body |  |
| Participant 45: “Before the operation I could not  experience much fun. I was always tired and not  in the mood to go somewhere. Now I have lost  27 kilos. I enjoy going out with my husband and  daughter, especially because I am less tired.” | Weight loss has given energy to take part in family and social activities | A healthy and functioning body | **Positive body experiences- not only the look but also the function** |
| Participant 193: “I have terrible hair loss; my hair  has become so thin. My nails grow badly and are  fragile. This in spite of careful intake of calcium  and multivitamins.” | The surgery had  consequences on how the body takes up vitamins  (effect on hair and nails) | A healthy and functioning body | **The negative side—side effect of the surgery** |
| Participant 49: “To see my daughter to grow up  and to enjoy being with her, to be able to hug her  and to hear her say how much she loves me  before she goes to sleep at night.” | Daughter gives meaning and feelings of love | A healthy and functioning body | **Be healthy- live long and having a family** |
| Participant 148: “And if I get too little sleep, I can  function relatively well the next day.” | Works pretty well the next day despite little sleep | A healthy and functioning body |  |
| Participant 122: “I am on my way to becoming  friends with my body again.” | be happy about the body again | Positive body image | **Acceptance of the new body** |
| Participant 49: “A few years ago an orthopedist  gave me little hope that I could walk after my hip  surgery. But I do walk, farther and farther.” | The body is no longer a hinder and even functions better | A healthy and functioning body |  |
| Participant 94: “We cooperate better and better,  my body and I. . . . The surgery and the recovery  made me aware that I should spend more time  on and for myself. Listening to my needs and  taking quality time for them. Now I just have to  put this into practice. . . . ” | The surgery and bodily changes gave awareness of listening to the body and its needs | Awareness of own feelings and needs | **Positive body experiences- not only the look but also the function**  **No ashamed feelings** |
| Participant 136: “I am now more conscious of  what I smell, taste, and feel, and therefore enjoy  food more. I try to be aware of what I eat and I  am surprised that the body works like this, that  this gives me satisfaction.” | The bodily changes brought forth more awareness of the senses | Awareness of own feelings and needs |  |
| Participant 116: “I can still enjoy something tasty,  but I do this in moderation and I fully enjoy it.” | Being in some control is pleasant | Awareness of own feelings and need |  |
| Participant 108: “I still feel big and fat and I feel  shocked when I look in the mirror or a shop  window and see a slim body. This still has to sink  in.” | Hard to comprehend the weight loss | balancing body and mind | **Accepting the new body and that identity** |
| Participant 122: “Taking care of my body has  been a struggle for years. For a long time, I didn’t  think I was worth it to look after myself. Why  would I do that? It was and would be terrible.” | Feelings of worthlessness, hard to motivate taking care of health and body | Awareness of own feelings and need |  |
| Participant 159: “I do notice that my appearance  has become more important to me. I go regularly  to the beautician, nail studio, hairdresser, and  pedicurist. . . . Now that my feminine shape is  reappearing, clothes have become more  important.” | Improved appearance motivates taking care of the body and caring about how it looks to others | Womanliness | **Appearance is important** |
| Participant 43: “There is nothing wrong with my  libido, but it is sometimes difficult to surrender to  it because I don’t find my body beautiful. . . . My  husband has remarked that he was bothered by  my body.” | Body shame and appearance was hinder to enjoying sex | Womanliness  A healthy and functioning body | **New body- no shame**  **Better sex life** |
| Participant 136: “Even though I love my wife I  have to push myself to be physically intimate  with her. . . . I sometimes say jokingly that they  have cut away my libido too.” | Having sex has become a struggle -no libido | Womanliness | **The negative part- better body appearance but no libido due to surgery** |
| Participant 49: “Feeling emotions is important  but sometimes very difficult. Emotions and  feelings are things that I like to put away.” | Hiding feelings as protection | Awareness of own feelings and need |  |
| Participant 148: “I have been through quite a lot  (rape, loss of my child, lost a parent to cancer,  taking care of my mother who had a stroke and is  now partially paralyzed, living with and being  beaten up by an alcoholic, and now an autistic  ‘ADHDer’ and an ‘ADHDer’ as a child. | Life has been hard |  |  |
| Participant 45: “The one thing I still struggle with  is whether people are still looking at me because  I am still fat or something. . . . I still can’t eat in  public, because it feels like they are all staring at  me and are thinking what she is eating, that’s so  wrong.” | People’s attention gives unpleasant feelings, reminders of being fat. | balancing body and mind | **People looking remind about a fat body** |
| Participant 108: “People approach me much  more these days. But it gives me mixed feelings:  Was I less worthy when I had a fat body?” | Why is value in bodily appearancee | balancing body and mind | **How people treat differently depending on bodily appearance** |
| Participant 152: “Before surgery I very much kept  to myself. I think I tried to build a cocoon around  myself so that others could not touch me. The  more weight I lose, the more I lose my insecurity,  and I talk and laugh more. My cocoon has really  disappeared.” | Losing all weight gave security/ self-confidence | Positive body image | **Effects on security and self-esteem** |
| Some related this to their recovery postsurgery; others described their body becoming stronger despite not having been well taken care of for many years. A similar number of women described  their body as a “work in progress.” | Weight loss improves the body’s function  Life after surgery is a process toward an improved life | A healthy and functioning body | **Be healthy- live long and having a family** |
| They appreciated the perceived improvements they had experienced  in their body functionality, while  acknowledging that there were still changes to be made. | The body is improved, but still there are many changes that need to be made | A healthy and functioning body | **Be healthy- live long and having a family** |
| Over a third of participants expressed appearance concerns, such as worries about excess skin and hair loss. A few women described that they had  not adjusted to their slimmer body and expected to see a heavier person in the mirror. Others explained that they found it difficult to care for their appearance after years of self-loathing. | Appearance concerns about visible bodily changes bring worries  It is difficult to adjust to a slimmer body and to change opinion of self-value | Positive body image  balancing body and mind | **Side effects affecting emotionally**  **Self-value is in one own mind** |
| Relatedly, almost a third of women described social concerns and weight stigma, such as avoiding eating in public due to fears of ridicule, and having  mixed feelings about improvements to social interactions (i.e., They were the same person on the inside, so why did others not accept them before  they had lost weight?). | Avoiding unwanted attention from others  Valuing oneself based on society’s opinions on what is worthy in a person | balancing body and mind | **How people treat differently depending on bodily appearance**  **People looking remind about a fat body** |
| In contrast, almost a third of women described increased appearance evaluation and investment  postsurgery, such as enjoying clothes shopping and feeling more satisfied with their appearance. Almost a third described social freedom and confidence,  and felt more accepted in social gatherings. | The changed body and lost weight motivated an interest in one’s appearance  Feeling more self-confident now when they fit in | Womanliness  A healthy and functioning body | **Appearance is important** |
| Nearly a third of women described difficulties coping with emotions and trauma. Some described past traumas; others described that after surgery  their emotional life had become tumultuous, and they had difficulties coping with this. Many women described barriers to sexual pleasure—for  example, because they worried their partner would dislike their body. | Past traumas affected emotions, and the surgery brought past feelings to the surface  Fear of other’s disgust hindered their sex lives | Awareness of own feelings and need  Womanliness  A healthy and functioning body | **A non-feminity body could make partners dislike** |
| **2 Condori et 2019** |  |  |  |
| Independently of having previous experience of not being obese, the participants expressed how they wanted to  return to normality, describing obesity as an obstacle to move forward with their lives and to have a family: | Obesity was preventing them from enjoying life and having a family | Womanliness  A healthy and functioning body | **Be attractive and Be healthy- live long and having a family** |
| I want to get back on my feet first and I want to be comfortable with my body before I . . ., I look at it this way, that if I’m not content then I can’t be a role model to my child. . . . Because they  see more than you think. I’d rather be done there. I am dreaming of having children, but it’s just not now. (Participant 8) | Need to be a normal-weight person, pleased with the body, to be a good parent and take care of a child  Wanting children but waiting | Womanliness  A healthy and functioning body | **Be attractive and Be healthy- live long and having a family** |
| The participants described a feeling of how life was set on pause since they had become obese. They talked about being inhibited both psychologically and physically, and  that this would turn back to normal when they lost weight, described as “the real me” is in there, somewhere: | Weight kept them from taking part in life and hindered them from being themselves | A healthy and functioning body |  |
| Like, I’ve always had this ideal body, that I don’t want to be super skinny, not at all, but a bit chubby, like, still having the curves. I  don’t like to be the way I am now, for example, that I’m like, overweight. . . . I’ve got an ideal body. It’s just hiding, somewhere in here, right now. (Participant 3) | The obese body hinders the person to be the person it wants to be, having a body with curves, not skinny | Womanliness  A healthy and functioning body | **Obese is a hinder to be a woman in all senses** |
| Several of the participants described a feeling of being alienated from themselves. Looking in a mirror or at a photo, they did not recognize the “other” person that they had become with the overweight: | That overweight person in the mirror is a stranger | balancing body and mind |  |
| Yeah, kind of. I’m feeling like a stranger, almost, like, in my own body. Because I still see myself as the size I used to be, like, when I went to high-school, in the beginning of high-school before things started to slip. I’ve even kept clothes, as if, from back then, because that’s like, the size I’m supposed to be, and that really wasn’t slim  but more the way I was comfortable. (Participant 4) | The overweight person has taken over the real person, and the body | balancing body and mind | **In mind the person they were as normal weighted** |
| I mean, I can go out and see people, but I kind of get stomach aches and I feel real sick, but it is possible, I mean, I do survive. ‘Cause  before I met her (the CBT therapist) I couldn’t, but then I had both lost some weight and regained some of my self-esteem. I guess I  think it’s difficult to tell if it depends on the weight or the selfesteem, but I think both go hand in hand in my case. (Participant 12) | Self-value are put into the weigh and how others looks at a big body. | balancing body and mind | **How people treat differently depending on bodily appearance** |
| The majority described the stigma of being  obese, and that they already “knew” what others were thinking about them. This was holding them back from going out  and building new relationships, which led to isolation. Losing weight was considered as a means to be more outgoing, being  able to be more open to new relationships: | Losing weight was a means of becoming part of society and feeling valued as a person | Positive body image | **Effects on security and self-esteem**  **How people treat differently depending on bodily appearance** |
| Well yes, if you consider the “love-part” I think it could affect in a positive way, hopefully then, erh . . . since I don’t have a lot of people around me, but the ones I have are very close, erh, and it  might also affect friendships positively. Because it feels like I have lost many since I myself have withdrawn. (Participant 9) | Losing weight could mean that is easier to love oneself and that makes it easier to love others | Positive body image  Awareness of own feelings and needs | **Self-esteem** |
| Then, when I said that I had applied for this kind of operation, he let this one slip: “but then you’re gonna . . ., then you might dump me when you become . . .” and I just; “when I become what?,” I said. “Don’t finish that sentence now ’cause then you’ll get a hell, so to speak. Do you think I’m with you because it’s like, some sort of a  consolation prize.” (Participant 1) | Being with a partner for the person he is, and he should feel that way about me—not a consolidations prize | Womanliness  A healthy and functioning body | **The person- not just the body** |
| Although nobody reported that they had received any explicit negative comments on their bodies in a sexual content,  sexual desire was inhibited by their own thoughts of how their bodies would look in a sexual situation: | Own thoughts and shame of body hindered sexual activities, | Womanliness  A healthy and functioning body | **Be attractive and Be healthy- live long and having a family** |
| And about sex life and so on . . ., it’s ugly, you must have a nice body. (Participant 11) | Sex does not fit with a big body | Womanliness  A healthy and functioning body | **Obese is a hinder to be a woman in all senses** |
| Losing weight was described as a means to get more confident in a sexual situation, and they were hoping that they would be more relaxed in their sex life: | A normal size figure would enhance sex life | Womanliness  A healthy and functioning body | **Obese is a hinder to be a woman in all senses** |
| Better. I mean I think it’s gonna . . ., I mean I think . . ., ‘cause I think . . ., I think that then you might dare to take more initiatives,  too. If you’re comfortable with your body, then I guess it’s more that you take what you want. So, I suppose it’s gonna . . . Because it’s like that, that if you’re feeling self-confident then everybody  else is noticing as well. (Participant 12) | Normal body size and appearance will give confidence and self-esteem | Womanliness  A healthy and functioning body | **Effects on security and self-esteem**  **How people treat differently depending on bodily appearance** |
| I really enjoy being active so that’s not the problem. To me, it’s the food. Erh, and then I just got to that point when I felt like, that you  don’t get any younger. Look, I do not care about getting older, like I’m 28 now, but it’s not getting easier and I’ve looked things up and  I don’t think having a gastric bypass is taking the easy way, I rather see it as the last resort. (Participant 1) | The food intake is a problem, hard to control so the surgery is the last resort |  |  |
| There was great awareness of the negative  effects of obesity on female fertility. Gynecological problems such as polycystic ovary syndrome, menstrual irregularities,  and endometriosis were spontaneously mentioned as contributing to the urgency of losing weight. The participants  considered obesity to be the most probable underlying mechanism to these problems. For most of the participants, the main purpose of the operation was not to achieve pregnancy, but all of them saw the picture of improved possibilities to get pregnant as another positive and important part of  having bariatric surgery | There was a knowledge that obesity was damaging fertility and the ability to have children  There was hope that the surgery would improve this  Achieving pregnancy was a positive side-effect of the surgery | Womanliness  A healthy and functioning body | **Restored fertility and possibilities to become a real woman—being pregnant** |
| Yeah, like I told you, that when you lose weight you get your period and then when you get your cycle going and, like, regular then you’ll have a baby. You can have children. That’s no problem.  (Participant 11) | Weight loss affects fertility, and hopefully, the surgery enhances natural cycles | Womanliness  A healthy and functioning body | **Restored fertility and possibilities to become a real woman—being pregnant** |
| Several participants described irregular  menstrual periods and hormonal problems as the underlying mechanism to their weight-gain. They also had great  expectations on that weight-loss would regularize their cycles and improve their hormonal balance: | Hormones caused the weight-gain and weight loss will improve fertility | Womanliness  A healthy and functioning body |  |
| I have PCOS and so on, which led to my overweight. I gained 35 kg and noticed that something had to be wrong. So, I went to the  Youth Service and she started to suspect that it was PCOS, but I was referred to the gynecologist to see and then they almost instantly confirmed that. We got to see the picture and there were  a lot of cysts and then I gained, like, another 20-25 kg after that. So, it all happened very fast. In a few years I gained a lot of weight, since it started. (Participant 4) | A cyst on the ovaries caused weight gain |  |  |
| Most of the participants pointed out  the irregular menstruations caused by obesity as the greatest obstacle to get pregnant: | Knowledge about that obesity effects fertility | Womanliness  A healthy and functioning body |  |
| . . . since I have not succeeded to get pregnant and I think it depends a lot on my weight. (Participant 7) | Being over-weight hinders pregnancy | Womanliness  A healthy and functioning body |  |
| Several of them also had knowledge about the negative effects of obesity on pregnancy and mentioned the increased risk of miscarriage: | Obesity is a risk to future pregnancies | Womanliness  A healthy and functioning body |  |
| No, but really, you never know why you have a miscarriage. That is . . ., it’s like you could never really know, but I’m also aware that you’re more likely to have a miscarriage and so on, if you’re  overweight. (Participant 12) | Obesity is a risk to my future pregnancies | Womanliness  A healthy and functioning body |  |
| And then me and my partner want to have children. Then first you must live together for a year, I think it is, it used to be two, I’ve  heard. So, we can apply for it now, but it’s no use trying to apply since I’ve got to get below BMI 30 to have an insemination or IVF.  (Participant 4) | There are so many demands when doing IVF and losing weight is a big one | Womanliness  A healthy and functioning body |  |
| All participants described a wish of having  children in a more, or less, close future, and that having a family was very important to them. None of them had heard anything  negative about pregnancies after bariatric surgery: | Having a family is important  Doing the surgery will enable pregnancy | Womanliness  A healthy and functioning body |  |
| No. And I know that it really doesn’t stop you to get pregnant. . . .  So that’s why I don’t consider it any problem. Because I know that you still can get pregnant. Yes. . . . Because otherwise . . ., like if I  couldn’t get pregnant . . . then I ’d never have the surgery. Because that’s my biggest dream in life. That’s just having children. So . . .  (Participant 12) | Wanting children that is the motivation for the surgery | Womanliness  A healthy and functioning body |  |
| The participants knew that obesity  causes high-risk pregnancies, and that this meant a risk for mother as well as child. Participants also mentioned that pregnancy  could lead to extra weight-gain which might put them in an even worse situation: | Obesity is a risk factor in pregnancy and it could also cause weight gain that could be worsen | Womanliness  A healthy and functioning body |  |
| And then I went to get new birth control pills and then I was talking a bit with the midwife and then she actually said that it would be  danger . . ., that it could be dangerous to both of us, the fetus and me, if I got pregnant because of the obesity so to say. So, I guess it would  be a lot easier if I lost weight. I really don’t want it to be dangerous neither to me nor the baby if I get pregnant. (Participant 2) | Obesity is a danger to future pregnancies and children | Womanliness  A healthy and functioning body |  |
| None of the participants were worried that bariatric surgery would affect future pregnancies negatively. Some of them were reflecting on the possibility that the fetus would suffer from lack of nutrients,  but the fact that friends and family members who already had gone through the operation had delivered successfully  afterward was encouraging enough: | Having obesity is more of a danger to pregnancy and fetus than having the surgery | Womanliness  A healthy and functioning body |  |
| We were discussing a bit, me and my cousin then and another friend who also had had the surgery, that since you can’t eat that much, I mean I myself, so that the baby can get sufficient nutrition and so on, but since others who had the surgery have managed well, so probably the baby gets sufficient nutrition, it seems so, erh,  that’s probably the only thing I’ve really considered . . . actually.  (Participant 7) | Others who had a surgery had pregnancies going well | Womanliness  A healthy and functioning body |  |
| I’m thinking a bit ahead. My parents really were, like I said, both of them, huge before, but they have lost weight and it took them a life-time so I’m scared that it’s like, going to take a lifetime and then . . . before something happens, and I don’t want that. While I am still young . . ., yes. I still haven’t had children yet and, like, all that  stuff. Because they were like, real big when we were . . ., when they had us too, me and my sisters. And then . . ., erh, they haven’t had  the energy to be as active as maybe other parents have been and so on and I don’t want that, if I’d be a mum one day. (Participant 10) | An active parent is a good parent and that’s what they are striving for | A healthy and functioning body | **Be healthy- live long and having a family** |
| **3Condori et 2020** |  |  |  |
| The participants described how they were now much more satisfied with their own body and appearance. Self-esteem felt  improved, and inhibitions were lowered. | Now more comfortable with their body and satisfied with their appearance and gaining self-esteem | Positive body image | **Positive body experiences- not only the look but also the function**  **No ashamed feelings** |
| Several of the participants said that they no longer had thoughts of what other people might think of them and their body. This had previously restricted them in several everyday areas of their lives. Reflections about how it might have been all in their own head before came up, but nonetheless they now felt liberated from these worries. | The weight caused them to be inhibited by other people’s opinions, but now when they lost weight they feel free and don’t bother | balancing body and mind | **How people treat differently depending on bodily appearance** |
| “Now, I no longer feel like it’s uncomfortable to go exercising among other people, like they’d be thinking “what is she doing” and so on. I can do  that. I am much, much more comfortable in social contexts, like I said. Going to birthday parties and, like hanging out with friends and so on, that feels  great too.” Participant 10 | Weight loss and fitting in give more confidence and worth of being with others | Positive body image | **Positive body experiences- not only the look but also the function**  **No ashamed feelings** |
| The participants described the process of finding themselves again. Some of them referred to the normal-weight person they  had been before, which seemed easier than for those who never had a normal-weight version of themselves before. | It takes time to find an identity when the outside changes, from over-weight to normal -weight | balancing body and mind |  |
| “It’s more that I’ve come back to what I should be like. Then I didn’t get this, like others did: “oh, I don’t think that I look slim” but I instead start to recognize myself again, like, “hey, this is me”. So,  it’s a bit different to me.” Participant 4 | Previously being thin makes it easier to accept weight loss--recognizing | balancing body and mind | **In mind the person they were as normal weighted** |
| “I guess that deep inside I’m always gonna be the fat one.” Participant 1 | Having an identity as obese | balancing body and mind | **Accepting the new body and that identity** |
| “I have to tone the belly there” and then “I’ve gotta buy some boobs.” Greedy, one could say that I am, or you just want to get better and better. I think now, it’s not about., if you think about when you  were younger, it was all about being thin and slender, sort of. But now I want, now it’s the muscles that I’m looking for and so on..” Participant 1 | Striving for a healthy appearance | A healthy and functioning body |  |
| Life had changed; as the body was lighter, things in everyday life got easier. This facilitated exercising but also accepting invitations for social events and new activities involving physical activity—making the participants more outgoing. | Losing weight give confidence and social and physical activity becomes easier | A healthy and functioning body | **Effects on security and self-esteem** |
| “I feel much better, I’m more comfortable. Like, I can be with other people. That’s fine. It doesn’t feel  awkward anymore.” Participant 5 | The weight loss makes you fit in, not being awkward | Positive body image |  |
| Shortly after surgery, several of them had met a (new) partner, and the ones that had already been in a stable relationship before surgery had got married or were planning for marriage. | Surgery gave confidence and made relationships easier | Womanliness  A healthy and functioning body | **Effects on security and self-esteem**  **Being attractive** |
| “Definitely. I’ve found my other half now. I have. So that’s a lot. He’s comfortable with me, and I’m comfortable with him. He’s not judging. You can notice that he likes me and the way I look.”  Participant 8 | Weight loss and new confidence make it easier to have a relationship | Womanliness  A healthy and functioning body |  |
| Most of the participants described a more active sex life, which was also more satisfying than before surgery. Internal factors, such as being more comfortable in a sexual situation and enhanced self-esteem, allowed them to demand more of their  partners. | Self-esteem and confidence allowed them to enjoy sex and also demand more from their partner | Womanliness  A healthy and functioning body |  |
| Being comfortable with the body and being naked, led to more intimacy. Sex was also a lot more enjoyable when one was  able to let go of the thoughts of how one’s own body might appear. | Proud to show off the body and enjoy being with partner | Womanliness  Positive body image |  |
| “And there was definitely no talking about positions before..one didn’t dare to, of course, but now I’m not uncomfortable at all, like feeling that I have to sit and hold my belly, or that when you lean forward you have to hold everything in place “- No, you can’t look there”” Participant 8 | No longer ashamed of the way the body looks, and that gives a feeling of comfort | Womanliness  Positive body image |  |
| The enhanced self-esteem made it easier to make demands. The participants talked about feeling relaxed about guiding the  partner to better sex, and the stimulation needed to reach climax. | Increased self-esteem enabled better sex and by allowing to make demands on partners | Womanliness  Positive body image | **Effects on security and self-esteem**  **Being attractive** |
| “. and then not being afraid of saying what you want and so on. So just,.really, to be comfortable with yourself leads to a thousand other things around sex that makes it a much, much better  experience and makes it more pleasant, and makes it, like, easier to have orgasms.” Participant 10 | Being comfortable with yourself and enjoying sex | Womanliness  A healthy and functioning body |  |
| Several participants described increased desire and a more active sex life. They pointed out factors such as increased energy levels and endurance, which made having sex more interesting.  Another participant pointed out less need of lubricants as the cycle had become more regular and ovulatory. Yet another said that  the weight loss had increased “the feeling of having intercourse.” | The weight loss improved physical functions and that improved the experiences of sex | Womanliness  A healthy and functioning body |  |
| “I had some difficulties getting wet before . since my ovulations started again, we haven’t needed much lubricant at all.” Participant 4 | The sex hormones are functioning again after the weight loss, no need for a substitute | Womanliness  A healthy and functioning body |  |
| “I., well since., because I’ve got this depression I’m not in the mood for intimacy., that is neither intercourse nor closeness at all, really. It feels like  I’m rejecting him, which I don’t want him to., I mean, to feel. So, in that way it’s worsening the entire situation, so to say.” Participant 3 | Depression is affecting everything negatively, including intimacy | Womanliness  A healthy and functioning body | **Emotions affecting intimacy** |
| One of the participants had already become a parent, and a second was pregnant. The other participants said that they  wanted to have children in the future, but not all of them felt ready to get pregnant yet. Having regular cycles was considered  very positive, as a marker of female fertility. | The weight loss has made them womanliness, having cycles and being fertile | Womanliness  A healthy and functioning body |  |
| Most of the participants now had a regular cycle and expressed their joy at feeling like a normal woman again. They talked about  feeling relieved as the body was working as it was supposed to again. For the participants who wanted to conceive now,  ovulation was very important. | Weight loss restored hormone cycles and makes them feel like women again | Womanliness  A healthy and functioning body |  |
| “And people are complaining about their.., I love my period.” Participant 4 | The restored period makes them feel womanly again | Womanliness  A healthy and functioning |  |
| Some of the participants had gone through surgery to enhance their fertility and were now trying to conceive. Another 2 had  already got pregnant when they had met a partner. All participants were planning for children in the future. | Did the surgery to lose weight and be able to become a mother | Womanliness  A healthy and functioning body | **Being a woman—a mother** |
| “I hope., so before., I haven’t got pregnant before, but I was rather thinking that I can’t get pregnant, but I’m still hoping that I actually could. I really would like to have a family at some point.” Participant 3 | Wanting a family and hoping that the weight loss will help | A healthy and functioning body |  |
| When talking about not being ready to get pregnant, other factors than weight loss were described as important, such as  getting to know the partner better or having stable economic  circumstances. | Many aspects in preparation for having a family | A healthy and functioning body | **Be able to work. Financial issues before having a family** |
| “No, not really, since he was not done with his studies and didn’t have a permanent job, err, so we never got that far. We didn’t. Like I told you, these  are priorities that you want to be done with before having a family.” Participant 1 | You need to be prepared to have a family | A healthy and functioning body |  |
| Several of the participants planned to postpone pregnancy until 2 years after surgery, on the advice of healthcare  staff. | Follow recommendations to ensure a healthy pregnancy | A healthy and functioning body |  |
| Although most of them now had a regular cycle, several talked about feeling stressed about fertility and said they still did not feel  certain they would conceive when they felt ready. Some had friends who had experience of infertility, while others referred to their own previous difficulties conceiving. | Worries about that obesity have damaged fertility | A healthy and functioning body |  |
| “I: And now you’re thinking more about it (having children) then?  R: Yes, actually. Then this with., yes, but since we tried before to achieve a pregnancy, and I didn’t conceive and so on. Probably I’ve got difficulties  with that, I mean.” Participant 6 | Tried for a long time to become pregnant, perhaps losing weight doesn’t matter | A healthy and functioning body | **Wanting a family** |
| **4 Faccio et al 2016** |  |  |  |
| In people who have already faced bariatric surgery, feelings of failure seemed to arise from the memory of the past:  ‘I lived with difficulty’, ‘I remember moments of collapse’, ‘I threw my life to the wind’, ‘I tried and it did not change anything’. The experiences related to the preoperative period  are, therefore, very similar in the two groups; however, one year after, the person describes herself differently. A new position related to the action emerged to the perception  of ability and to the will of dealing with new situations. Participants now stated: ‘I assert myself’ or ‘I explore myself,’ ‘I can finally get out’, ‘I can go shopping,’ ‘I can be  myself’. | A fear of the past and a fear of failures returning  Weight loss made a new person and the old one is not wanted back | balancing body and mind | **Fear of failure** |
| Regarding the relational area in the reoperative group, the ‘others’ were experienced as investigators, or persecutors: ‘Others have hurt me’, ‘I feel ashamed’, ‘People look at me’, ‘I do not feel accepted by some people’. The ‘other’ person was perceived as someone who does not recognise one’s own subjectivity. In the postoperative group, the ‘other’ stopped being perceived as a judge and the person finally  feels as if she belongs amongst others, ‘to be accepted by others’, ‘I go out without being noticed’, ‘I’m normal’. | Society is harsh if you are overweight  By losing weight you fit in and become accepted by society | balancing body and mind | **How people treat differently depending on bodily appearance** |
| Another semantic area that emerged only among participants, who had already been operated on, revealed frustration and difficulties in facing the consequences of surgery, was that of experiences related to the body changes. In this  area, participants gave phrases like: ‘(. . .) it was hard, seeing so many changes and so quickly, is somewhat cruel (. . .)’, ‘(. . .) I did not expect that the change was so sudden (. . .) it was all too fast (. . .)’, ‘(. . .) I thought it would be easier (. . .).’ Statements that revealed inadequate preoperative preparation regarding the difficulty to adapt their selfimage to their new body: | The changes after surgery occur quickly and are hard to accept  Preparation is needed for what happens after surgery | balancing body and mind |  |
| It ‘s true that you lose weight in the body, you cannot loose weight in the same way in your head or, anyway, certainly not at the same speed. (MB, postoperative group) | It is hard to keep up mentally with the physical changes | balancing body and mind |  |
| I am an ex-obese (. . .) When I look in the mirror I see myself and I feel good-looking, . . . , when I try a dress, I can feel good. . . But when I think to myself, I guess I’m still chubby . . . maybe it happens  to me to see some girls around and to think. . ., such a beautiful body, that envy! | Accepting the new normal weight body and feeling proud but at the same time in mind being chubby | balancing body and mind | **Accepting changed body** |
| The mind has not yet adapted to the image of the new body and the new way of eating. Based on what we have presented above, it is not surprising that from the analysis  of the dominance between I-positions, it emerged that in both groups the ‘I am obese’ was the dominant position, although physically normal in weight, the position ‘I am  obese’ doesn’t become subject to another voice: it remains dominant, even among those who have already been operated on. | Although the weight has changed, still the identity is a person with obesity | balancing body and mind | **Accepting the new body and that identity** |
| In the postoperative group, answers appeared linked to the semantics of wellbeing:  ‘I’m fine,’ ‘I feel good’ but the dominant voice was still ‘I am obese’. We also found the positions: ‘my family’ ‘my partner’ and ‘my work’ were significant. The latter  position (the work) was completely absent in the preoperative group, which may indicate that the person was focussed on them before the operation and had no space for anyone else, while after the surgery the relational component  becomes even more important. | Losing weight makes it possible to focus on others and relations | Positive body image | **Positive body experiences- not only the look but also the function**  **Being able to love one self and thereby love others** |
| Women in the preoperative group anticipated that their life could ‘magically’ change  after the bariatric surgery. They found it difficult to distinguish between real and concrete life experiences that a thin body would allow them to live, from the more profound changes that do not depend on weight, such as those relating to the friendships and relationships with the opposite sex. Some participants of the postoperative group, on the other hand, reported continuing to limit their lives. They  imagined that so many experiences would change automatically with the weight loss, but it did not happen, because those experiences were related to their personal selfesteem: | Losing weight does not magically make life around easier, it is something you have to work on  Strengthen self-esteem | Positive body image | **Positive body experiences- not only the look but also the function**  **Being able to love one self and thereby love others** |
| Before (the surgery) I thought everything went wrong because of these extra pounds, I thought that everything depended on my obesity:  if I stiffened in front of a guy, it was because I was ashamed of my body and because I was worried about what he could see in my body; (. . .) I just saw around me successful people, while I always remained there, large and lonely; if I could not wear nice clothes, if I could not wear makeup: it would be like seeing a  whale walking with handbag and heels (. . .) But if I lost weight?  Then yes, I would begin to live! Everything would be better! (. . .). (S. group post-operative) | It is hard to keep up mentally with the physical changes | balancing body and mind | **Accepting the new body and that identity** |
| In some cases, the person has become aware that their body shape was covering other personal uncertainties and the weight loss only made other problems evident. In other  cases, very favourable personal experiences testified the beginning of a different life phase: | Accepting the new normal weight body and feeling proud | balancing body and mind | **Accepting the new body and that identity** |
| Under the flab I found a new me, with a different ability in relationships.  I’ve become a less accommodating, less funny girl. I no  longer need to make myself agreeable with filters that do not belong to me, in order to be appreciated. I became more reflective and less instinctual. | Although the weight has changed, still the identity is a person with obesity | balancing body and mind | **Accepting the new body and that identity** |
| **5 Guven et al 2021** |  |  |  |
| Women described relationship problems caused by their weight. They mentioned feeling of distance, deterioration physical intimacy, and restriction their social lives. | Overweight pushed other people away | Positive body image | **Strengthen self-esteem and not being ashamed** |
| Some of the women stated that relationships with their spouses worsened due to being overweight,  and some described untold emotional distance between them. A woman stated that she decided to have surgery only because her husband cheated on her moreover; another woman reported that she was planning to divorce after the surgery. | Relationships with spouses were affected by the over-weight and surgery was a way to become accepted | Positive body image | **Strengthen self-esteem and not being ashamed** |
| My priority was my health, my marriage was so important to me, but I have tried to break down of my marriage many times, because my husband was bothering me then. I didn’t want him, I was repeatedly saying that “I will  divorce”, “After the surgery, I will divorce that man!” (P14, Age 42) | Weight loss strengthens confidence to leave an unhappy marriage | Positive body image | **Strengthen self-esteem and not being ashamed** |
| Women described physical distance between her and her husband due to snoring and sweating at night. In some cases, it was women who wanted to put some physical distance such as having separated their beds, to avoid sitting next to him or trying to not being seen by their husband. However, in some cases, husband verbally demanded staying far or not being together with her | Feeling disgusting and husband made that very clear | Womanliness  A healthy and functioning body | **Effects on security and self-esteem**  **Being attractive** |
| My physical condition was influencing our relationship… Normally we do not touch each other. He and I are feeling uncomfortable… He was keeping himself at bay, feeling estranged from me… (P1, Age 35) | Husband did not find wife attractive and stayed away from her | Womanliness  A healthy and functioning body | **Effects on security and self-esteem**  **Being attractive** |
| For example, that is, my husband always wanted me to lose some  weight, well, he would say, “You are overweight, you are not matching  me when we go somewhere together”… After all, I was sleeping apart from him, I didn’t want him to see my body, since I would snore, even all the building would hear me, not only my husband,  therefore, I would get embarrassed… (P14, Age 42) | Husband was ashamed of wife and her weight  Being ashamed of the body and its appearance | Womanliness  A healthy and functioning body | **Effects on security and self-esteem**  **Being attractive** |
| Limitations in social life may result from women’s concerns about their appearance. Some women expressed their reluctance to go out with their husbands and friends, because they could not find an outfit that suits well with them and they did not like what  they could find to dress. Although some women did not report any lived  experiences with their husband, another concern that prevents women from  going out is the fear of bad smell to their husband and people around them | Feeling disgusting and feeling that you do not deserve to be around other people. Being ashamed of their revelation | Womanliness  A healthy and functioning body | **Effects on security and self-esteem**  **Being attractive** |
| Nobody would like to sit near to you. I sweat a lot. Whenever someone sits next to me, I ask myself, “Do I smell awful? Is it obvious to the person near me?  It was more social problem, to be honest I did not have an apparent problem with my husband, I guess he would want to go out, and have a thin wife to go out with him, because we not used to go out together. (P16, Age 44) | Husband ashamed of having a wife with obesity, not fitting the norm | Womanliness  A healthy and functioning body | **Effects on security and self-esteem**  **Being attractive** |
| Women stated that their excess weight caused sexual problems by affecting  the appearance of their bodies, physical activities, physical health, sexual  functions, and their perspective on sexuality | Not feeling attractive and sexy because of over-weight and that affected self-esteem | Womanliness  A healthy and functioning body | **Effects on security and self-esteem**  **Being attractive** |
| A few women expressed a decrease in the quality of their sexual lives and romantic relationships with their husbands. This negative effect on their sexual life resulted from different reasons such as lack of self-esteem, feeling unattractive, and body image impairment. Some women  described decreased self-confidence and feeling uncomfortable with the  idea of bad smell due to sweating that prevent them from having intimiate  relationship with their husbands | Ashamed of over-weight body and feeling unattractive causing withdrawal to an intimate relationship  The shame of bodily appearance hindered the enjoyment of sex and life | Womanliness  A healthy and functioning body | **Effects on security and self-esteem**  **Being attractive** |
| As I mentioned, I felt humiliated. My husband’s treatment of me, oh no! He was not insulting me, there was nothing verbal, but he was falling asleep right after intercourse, turning his back to me, then I was feeling abused. I don’t like it, I am a tactful person. Instead of turning your back to your wife, hugging, caressing, or speaking to her would  be more motivating. I would like to feel loved, as well. (P13, Age 45) | Husband made wife feel unworthy of love and affection due to over-weight | Womanliness  A healthy and functioning body | **Effects on security and self-esteem**  **Being attractive** |
| For instance, I was abstaining from showing my body to my husband  while the lights were on. Fat was spilling out from everywhere on  my body. (P8, Age 34) | Ashamed and disgusted by the obese body, not showing body |  |  |
| The difficulties experienced by women during intercourse due to excess weight and related problems involved inability to keep up with their husband, not being able to move, sweating, and pain. In some women, these difficulties caused struggle to satisfy herself or their husbands during intercourse or even sometimes made women not willing to have sex with their husbands. | Obesity hindered women from enjoying sex since they felt bad about their appearance and inability to move the way that was needed to enjoy | Womanliness  A healthy and functioning body | **Effects on security and self-esteem**  **Being attractive** |
| Well, I mean, you cannot raise your foot or leg, you cannot turn on your side, you cannot do anything!… The man thinks that he is sleeping with a robot… (P9, Age 43) | Obesity hindered women from being physically active in sex life | Womanliness  A healthy and functioning body | **Effects on security and self-esteem**  **Being attractive** |
| Honestly, we had never had a normal sex life; actually I think that I  deserved this cheating… I was unable to breathe, I mean, I did not want to be with him, I was like pushing the man away. (P6, Age 30) | Obesity caused the husband to cheat on the woman, and she deserved it  She pushed the man away | Womanliness  A healthy and functioning body | **Effects on security and self-esteem**  **Being attractive** |
| This is the problems that lead women having dysfunction in sexual life and prevent them from experiencing sexual satisfaction.  Women described decreased in sexual satisfaction, lack of lubrication,  and inability to reach orgasm or perceived lack of partner’s desire and  satisfaction during intercourse. | Obesity stopped women from functioning sexually and made them shy away from sex with their partners | Womanliness  A healthy and functioning body | **Effects on security and self-esteem**  **Being attractive** |
|  |  |  |  |
| How can I say, I was unable to orgasm. I mean, I was unwilling… When I was willing, he was not responding, and vice versa, this was the frigidity between us. (P1, Age 35) | Obesity made the women ashamed and hindered sexual activity | Womanliness  A healthy and functioning body | **Effects on security and self-esteem**  **Being attractive** |
| A few women stated that they see sexual intercourses as a duty and often sacrifice their satisfaction to please their husbands. Even one woman described sexual intercourse as a ‘death’ in which she does not have any pleasure at all (P15, Age 52). | Sexual intercourse was an obligation not a pleasure | Womanliness  A healthy and functioning body | **Effects on security and self-esteem**  **Being attractive** |
| I mean, I, only see it (sex) as a duty. That is, with regards to married people, we see the sex as a duty, seems like we do not have any other responsibilities. I mean, tidying up the house, cooking the meals have no importance, they are not obligatory, but this is… So, conscientiously I feel like that, every Friday, as a duty, I mean it is not something that is done because I wanted to. (P13, Age 45) | Sexual intercourse was an obligation not a pleasure | Womanliness  A healthy and functioning body | **Effects on security and self-esteem**  **Being attractive** |
| Being able to do activities that can be routinely performed by others, and  enjoying social life with their husbands were women’s expectations from surgery. | The women want to be able to take part in and enjoy life again | Womanliness  A healthy and functioning body | **Effects on security and self-esteem**  **Being attractive** |
| Women stated that they dreamed of going out with their husbands after surgery. In addition to limitation of social activity with their husbands, women also described negative effect of weight on their social life with their family and friends. | The women want to be part of social life and not be ashamed of themselves | Womanliness  A healthy and functioning body | **Effects on security and self-esteem**  **Being attractive** |
| So to take a ride around, to go to a place with my husband, to go to a store, and buy casual clothes, no such big size, there is no ‘not for you’, so you can walk around comfortable with him. (P11, Age 28) | Being normal and fitting in with society and feeling accepted | Womanliness  A healthy and functioning body | **Effects on security and self-esteem**  **Being attractive** |
| Women stated that they expected to be able to affect their husbands sexually  and to improve their sexual life with weight loss after surgery. | Being able to enjoy sex again and have a normal relationship with partners | Womanliness  A healthy and functioning body | **Effects on security and self-esteem**  **Being attractive** |
| Women expressed that they were expecting feel more satisfied in their sex lives, make their husbands jelous and feel their husbands’ desire much more than before. Most women stated that the  desire to make their husbands turn on with their appearence and to have  more intimate relationship | Losing weight and thereby getting partner’s attention again | Womanliness  A healthy and functioning body | **Effects on security and self-esteem**  **Being attractive** |
| Well, for me, sexuality is a very important thing. Because it is the need of my body and soul, I mean. I like having sex… Of course, I had always motivated myself with that dream, “Hey, I will lose weight, and I will drive you crazy!” etc… and I was thinking that his respect to me, which, thank God, already  exists, would increase. I mean, of course like every man, he wants to have a pretty wife nearby. (P7, Age 33) | Good sex life is the foundation of mental and physical health  Attention and appreciation are needed to reach this balance | Womanliness  A healthy and functioning body | **Effects on security and self-esteem**  **Being attractive** |
| Some patients reported that the surgery led to improvements in their marital relationships. Most of women mentioned that their husbands showed an increased interest in them, including an increase in sexual/ romantic behaviors. | Marriage became better when wife lost weight and husband accepted her | Womanliness  A healthy and functioning body | **Effects on security and self-esteem**  **Being attractive** |
| After surgery the weight loss and body compositon changes that women experienced made them and their husbands more intimate to each other. Particularly the surgery solved the problems of snorring and sweating that caused to women stay away from their husbands. Also the surgery helped women be more comfortable, share the same bed with their husbands, and enjoy physical intimacy with them. Some women expressed that their fear relieved and they did not show any resistance  to their husbands’ intimacy attempts. | Losing weight makes women feel good they want to show off their bodies to partners | Womanliness  A healthy and functioning body | **Effects on security and self-esteem**  **Being attractive** |
| When your husband comes home, you always hug and kiss, say welcome and my husband loves it, but I used to always run away. Because I was so sweaty, I could not wear anything, but, now we are like new lovers at the door again.  I mean, we are having our second spring… He always hugged me and wanted to sleep in touch with me, I always ran away. Because when he puts his hands to my body, he touches either my belly or my floppy breast. I am getting more comfortable with him now. (P16, Age 44) | The new body renewed love life  After losing weight there is a feeling of deserving love and appreciation from the partner | Womanliness  A healthy and functioning body | **Effects on security and self-esteem**  **Being attractive** |
| This is the feelings of women about overall relationship with their husbands. Even few women mentioned that the surgery saved their marriage by having better sexual life and relationship with partners. One woman stated that ‘We did not have a life before this surgery. We were like sister and brother. Now, we are a real couple again…’ (P4, Age 44) | The new body made women feel confident and want to take part in a romantic relationship with partners again | Womanliness  A healthy and functioning body | **Effects on security and self-esteem**  **Being attractive** |
| We would not chat before, he would not even look at me and my eye, there were quarrels and disputes, but now we look at each other, exchanging glances, chuckling, and having chats afterwards, cackling, I mean, while I am in the kitchen, he is coming and doing certain moves I cannot explain…. He is cheerful,  and I am cheerful too, many things have changed. (P14, Age 42) | Husband is no longer ashamed of wife and interacts | Womanliness  A healthy and functioning body | **Effects on security and self-esteem**  **Being attractive** |
| Women expressed that they felt more comfortable to go out with their husbands and felt that their husbands also more comfortable to go out with them. After surgery, the couples tend to spend more time together and go out in public. | Not ashamed of themselves and take part in social activities together with their partner | Womanliness  A healthy and functioning body | **Effects on security and self-esteem**  **Being attractive** |
| The way he looked at me changed… Before, we would never go out to drink a coffee, for example, we would even not share the same room at home… My man is now sitting with me cheek by cheek, holding my hand… (chuckling) (P6, Age 30) | After losing weight and having a new body partners accept them | Womanliness  A healthy and functioning body | **Effects on security and self-esteem**  **Being attractive** |
| Most of the women stated that an increase took place in the frequency of  sexual intercourse, while five patients mentioned that they experienced no  change. Women generally felt positive changes in their sexual life; however,  a few mentioned an increase in the amount of foreplay and sexual desire. | Sex life improved when the body changed | Womanliness  A healthy and functioning body | **Effects on security and self-esteem**  **Being attractive** |
| Many women described positive  increase in their sexual lives after the surgery. They pointed out that their sexual experiences were different, could not be compared with before the surgery. | Sex life improved when the body changed | Womanliness  A healthy and functioning body | **Effects on security and self-esteem**  **Being attractive** |
| Sexual life before the surgery was like a death, afterwards it revived.  (P15, Age 52) | Sex is enjoyed since there is no longer shame in themselves | Womanliness  A healthy and functioning body | **Effects on security and self-esteem**  **Being attractive** |
| I call him, for example… I say, “Let’s do something, come.” I am smiling, he is smiling, too (chuckling). Let me say, I turned out to be bawdy… For instance, when he wants sex, I do not refuse now.  We have intercourse whenever he wants now. (P5, Age 39) | Sex is enjoyed since there is no longer shame in themselves | Womanliness  A healthy and functioning body | **Effects on security and self-esteem**  **Being attractive** |
| Honestly, it is perfect now. (Laughing) I mean, everything is right on track, I can move comfortably. I can move any way I want, I can behave as I like, how can I say, it (sex) is easy and better now.  (P2, Age 42) | Losing weight gave physical function and that made sex enjoyable | Womanliness  A healthy and functioning body | **Effects on security and self-esteem**  **Being attractive** |
| Honestly, it is quite well. I would have another baby if I didn’t have  a tubal ligation… Yes, it is very well now, I used to say to myself  that I knew how to experience sexual pleasure… I never experienced  pleasure. For me, there was no life before. (P9, Age 43) | Losing weight and having a new body makes life enjoyable and gives sexual pleasure | Womanliness  A healthy and functioning body | **Effects on security and self-esteem**  **Being attractive** |
| In addition to experiencing an increase in sexual desire after the surgery, some women stated that their husbands’ sexual desire also increased, and having more satisfaction due to having more options in movements and motion during intercourse | Normal weight makes sex more enjoyable.  Partners are showing a sexual desire | Womanliness  A healthy and functioning body | **Effects on security and self-esteem**  **Being attractive** |
| Well, before, for example, he was not sidling up to me, but now, whenever he sees me… (chuckling) Before, he did not have this much desire. I mean, there was no problem even if I was absent for a month. But now, I am getting caught twice a month. (chuckling) (P5, Age 39) | Relationship with partner is improved after losing weight.  There is a desire and confidence with sex | Womanliness  A healthy and functioning body | **Effects on security and self-esteem**  **Being attractive** |
| Yes, in the words of my husband, he says, “Now we are wrestling, but before, it was not like this”. (P12, Age 29) | Husband enjoys intimacy now after weight lost | Womanliness  A healthy and functioning body | **Effects on security and self-esteem**  **Being attractive** |
| Although most women reported increased sexual desire and satisfaction, only one woman stated that their sexual life worse than before. | Sexuality is improved after surgery | Womanliness  A healthy and functioning body | **Effects on security and self-esteem**  **Being attractive** |
| We did not have a problem before, but right now we have. I have more pain  for instance… I don’t want it now. There is dryness and reluctance to engage  in sexual intimacy. Even, I cannot be satisfied. (P4, Age 44) | Surgery had a side -effect, sex has become painful | Womanliness  A healthy and functioning body | **Being attractive**  **But negative side effect of surgery-- hormones** |
| Women stated that weight loss and having a slim physical appearance gave them more freedom what to wear and increased their self-esteem after surgery. Some women described the desire to look in the mirror and likes what they see in. | Self-esteem is strengthen due to the new body and they want to show it off |  |  |
| My self-confidence has changed. I mean, I can go out now. I can speak with my friends, I’ve begun to attend social activities, and previously I was making excuses to my firends. (P8, Age 34) | After losing weight there is self-confidence and the women want to socialize and show themselves | Womanliness  A healthy and functioning body | **Effects on security and self-esteem**  **Being attractive** |
| I love myself in terms of appearance. I can wear whatever I want.  For instance, (before), I was not able to even tie my shoes normally.  (P1, Age 35) | Now the body can be used like a normal body and the appearance is satisfying | Positive body image |  |
| Many women stated that changes in women feelings, appearance, and relationship with ther husbands and overall positive changes in sexual life led women to feel being loved by their husbands again. | After losing weight and having a different appearance partners show love and affection again | Womanliness  A healthy and functioning body | **Effects on security and self-esteem**  **Being attractive** |
| Now, I say, I feel stirrings, as if we are having a new love or experiencing  it again, there is something, I mean he feels about me… It must be a mutual desire, since you feel it from the other side, so you reflect… Well, if I wanted to do it (sex) every day, he would agree.  I mean, we are as if just married. (P14, Age 42) | Something has changed since losing weight there is an awareness of sexuality and needs | Womanliness  A healthy and functioning body | **Effects on security and self-esteem**  **Being attractive** |
| I mean, there is a big love in my husband. It is something different  now, when I wear tights, for example, he recently said to me, “You look so sexy! I will never let you go out in those pants!” It was the first time I heard something like this from him… (P3, Age 35) | After losing weight and having a new body partners accept them | Womanliness  A healthy and functioning body | **Effects on security and self-esteem**  **Being attractive** |
| Women described their concerns and anxiety related being overweight and its effect on their relationship decreased after surgery. Women’s mood and demeanor became more pleasant and positive toward their husbands and toward others | The women were happy with themselves and thus they also became more comfortable with their partners and others around them | Womanliness  A healthy and functioning body | **Effects on security and self-esteem**  **Being attractive** |
| Before the surgery, I was a fighter. We had a lot of fights, and after these fights there was a distance between us. I used to feel that as if a stranger had touched me not my husband. I’ve had problems like that. Right now, but I’ve overcame all those problems. (P11, Age 28). | The new body brought comfort and calmed down negative feelings | Womanliness  A healthy and functioning body | **Effects on security and self-esteem**  **Being attractive** |
| **6 Jensen et al 2013** |  |  |  |
| Participants described their lives before BS as a difficult period where they had daily thoughts about weight loss, feeling displeasure and dissatisfaction with their body and appearance. They sed avoidance behaviours to protect themselves. This meant avoiding mirrors, not creating awareness around their personalities  and a lack of desire to meet new people. Participants had low self-worth and self-esteem. | Feeling disgusted with themselves because of overweight  Avoiding situations where they meet people, a protecting behaviour | Positive body image | **Strengthen self-esteem and self worth** |
| P5: It was because I thought it was overwhelming having to lose weight the natural way….. My sister said to me, ‘if you can lose 23 kg by yourself, for sure, you can lose 80 kg. So I said: ‘yes, I can, but it’s going to  take the rest of my life.’ | I am aware of my limits and what I can accomplish |  |  |
| P2: In the past, it was not fun at all for me to be in the city because I just sat thinking that everyone would  think I was fat……I wanted to be there, just not like me. If I just looked different. | Always thinking that appearance is an obese body to other people | Positive body image | **How people treat differently depending on bodily appearance** |
| P4: Was not together with my friends so much; they began to take a little distance from me. They wanted to  do different thing i.e. in the city and being with others. So, I went home and was alone, I ate for comfort….It  is my best friend…It is the food. | Friends distanced, food was comfort and companion | Positive body image | **Strengthen self-esteem** |
| P3: My friends could not give me a hug; I could not stand it…. Before I didn’t feel my limits, I just knew  that people should stay away from me, they should not know anything and I was just keeping everything to  myself. | Hard to stand physical contact. withdrawal because of an obese body | Womanliness  A healthy and functioning body | **Effects on security and self-esteem**  **Being attractive** |
| P4: The hardest thing was to get one’s brain in the right mindset, to get it converted…I knew what was  needed but I just wasn’t geared for it. I would not succeed. It would just go bad again, why really straighten up, whenever things were going bad anyway. | Found excuses for overweight | balancing body and mind | **Accepting the new body and that identity** |
| P1: It was kind of admission of failure that I had to have surgery to lose weight. I just thought so, but on the other hand I also took responsibility for my life and my future. | Being overweight had gone too far and surgery was a responsible solution | A healthy and functioning body |  |
| P5: You’re actually not hungry when you eat. Your brain keeps telling you that you are hungry. The stomach on the contrary is about to burst…and it’s hard to get rid of because your brain was operated on. This need, it’s not just removed in surgery…..There’s such a psychological need, all the time. | The hard part is changing the psychological need to eat. | A healthy and functioning body  Awareness of own feelings and needs |  |
| P4: I just felt bad, I began to sweat and it was dumping, I experienced….It was really horrible. I would actually  prefer to eat like a diabetic so I can be fairly sure that nothing is happening | The physical side effects can be very frightening | Awareness of own feelings and needs |  |
| P3: You just have to find out how much you actually can eat and what you can tolerate….It has been some  challenge navigating, such a labyrinth….One has felt some pain at times because you had to figure out what  you could do. But it has not been hard in that sense, just a challenge. I’m about to have managed it. | There is a lot to manage because of the surgical side-effects and effects |  |  |
| P1: I’ve been good to feel what my stomach tells. | Managing to understand the body | Awareness of own feelings and needs |  |
| P3: It is limited. It is ok….It’s just to find out that it’s okay. I do not get more than that….I have no cravings  in the same way, not at all. And if I get it, then there is the small bowl. That’s what I need. | Doing well dealing with the changes | Awareness of own feelings and needs |  |
| P5: It is getting all the things back that you’ve said no to over the years, it is quite amazing. | Enjoying life again | Awareness of own feelings and needs |  |
| P2: I’m not so afraid that people will say something about me, because I know that I’m about to be  transformed now. I know that I’m doing something for it and then it doesn’t really hit me anymore. | Since a good choice has been done, what people say doesn’t matter | Positive body image | **Strengthen self-esteem** |
| P1: I hope that I manage to stay on the right path and I believe in that. When you have become so pleased  with yourself and you know how it would be if you fell back, then…. | Pleased with yourself now and not wanting to return to past self | Positive body image | **Strengthen self-esteem** |
| P3: Now I have to take care again, so it’s a rollercoaster. Now I have to be stable, so it’s still sometimes hard…yes, it may go up and down a bit. | It is difficult adapting to the new life | balancing body and mind | **Accepting the new body and that identity** |
| P1: You feel better; you get approval then…..approval from other men. | Feeling better- getting approval from others—other men | Womanliness  A healthy and functioning body | **Effects on security and self-esteem**  **Being attractive** |
| P4: Having children, is now a part of my future. | Fulfilling the dream of children | Womanliness  A healthy and functioning body | **Effects on security and self-esteem**  **Being attractive** |
| P3: I’m actually not the same person at all, as I was before surgery. Now, it’s me that means something, this means that I will continue being the person I am now. | Changing as a person due to the surgery | balancing body and mind | **Accepting the new body and that identity** |
| P3:…But now there is suddenly a lot of skin that I have to look at. I do not think is so funny. There is still  something left, which means that you’re not quite there, where you have to be. But it is much better. I’m not finished yet. | All the skin is a reminder of past self | balancing body and mind | **Accepting the new body and that identity** |
| P4: It’s also something psychologically that has changed; you think more positively and believe that it is possible to dare something now. | Feeling better because the surgery gave a new body | balancing body and mind | **Accepting the new body and that identity** |
| P1: I want a normal BMI. | Want to be normal | Positive body image |  |
| One participant now allowed others physically to get close to her. | Allowing physical contact | Womanliness  A healthy and functioning body | **Effects on security and self-esteem**  **Being attractive** |
| Invincibility is metaphorically described as ‘being super woman’. | Nothing is impossible | Womanliness  A healthy and functioning body | **Effects on security and self-esteem**  **Being attractive** |
| The participants’ experience of body control may have led to a predictability that was experienced as a new-found energy and freedom, which seemed self-reinforcing through confirmation from others | Having control and being strengthened by confirmation from people around | A healthy and functioning body |  |
| Through the bodily, social and mental  well-being, all participants considered the opportunity of realizing their dreams of having children and raising a family | Being in balance and capable of having a family | Womanliness  A healthy and functioning body | **Effects on security and self-esteem**  **Being attractive** |
| In the past, they had been concerned about impaired fertility | Being afraid not being able to have children | A healthy and functioning body |  |
| Participants’ narratives depict improved opportunities in their life, where confirmation from others gave them a sense of equal status. | Acceptance from others is needed to feel valued | Womanliness  A healthy and functioning body | **Effects on security and self-esteem**  **Being attractive** |
| **7 Magdaleno et al 2010** |  |  |  |
| “I thought that I would solve my problems, that I would be happy, get a boyfriend…” P5 | A new body would change life, including a partner | Womanliness  A healthy and functioning body | **Effects on security and self-esteem**  **Being attractive** |
| The first source of relief, after postsurgical recovery, comes from a strong sensation of acceptance and social reinsertion. They feel that they are part of a world which  they were not a part of. They experience a feeling of genuine happiness. | Experience being part of and included in the group | Positive body image |  |
| “…I go by bus just to go through the turnstile; it seems that you ate a chocolate bar from so much happiness”. P6 | Want to reinforce happiness with the new body | A healthy and functioning body |  |
| The sensation in finding themselves again is lived with great pleasure and relief. | Finding themselves gives positive feelings | A healthy and functioning body |  |
| “…everyone says: wow, how you have changed! Ah, now you are back to how you were before!” P3 | Positive comments from others since losing weight | Positive body image |  |
| This process of the recovery of identity is lived as a born again, that the patients relate to a new life that begins after  being operated. It is a phase that is experienced with a lot of satisfaction. | Having a new start in life since losing weight gives positive experiences | A healthy and functioning body |  |
| “After I was operated, I was born again. I was born for a happy life. Before that, I was sad…” P6 | The surgery brought forth happiness in life | A healthy and functioning body |  |
| In the postoperative period, the women have to face new life experiences, such as jealousy, mistrust, fear, and envy  that, until recently, had not existed. | New experiences that people now react to their feminine appearance | Womanliness  A healthy and functioning body | **Being attractive** |
| “Now my husband has started to talk like this: go out for what, to show off? So, another phase has already started and I still have not learned how to deal with  it…” P3 | Negative reaction from partners because of feminine appearance | Womanliness  A healthy and functioning body |  |
| When these reactions of others start to become evident, reactions of those very people whose “acceptance” they believed to guarantee by their getting thin, they can feel profoundly disillusioned, sometimes confused. | You are treated based on the way that you look- not always positive | Positive body image | **How people treat differently depending on bodily appearance** |
| “Before they did not like me because I was fat. Today I am thin …they will think that I am stealing their scene! I did not operate for this …” P4 | People are very unfair in the way they treat you |  |  |
| **8 Magdaleno et al 2011** |  |  |  |
| Weight loss after surgery leads to marked improvement in body image and attractiveness,// The loss of weight is experienced as a valuable opportunity to recover a place in society. | You are accepted in society if you have the proper weight | Positive body image | **How people treat differently depending on bodily appearance** |
| Nowadays I am a more eager person, I feel like doing things and even go out from time to time…But the problem is dating…I just can't…..I'm ashamed…. P5 | Self-esteem not totally recovered—able to meet others but hard to date | Womanliness  A healthy and functioning body | **Effects on security and self-esteem**  **Being attractive** |
| After all the effort to find their place in the world again and to be admired, they are faced with the reappearance of  their feminine bodies: a new situation with which they are unable to cope. | Having a feminine appearance but hard to manage how people respond to it | Womanliness  A healthy and functioning body | **Effects on security and self-esteem**  **Being attractive** |
| It was as if I was inside a cocoon, there was no outside world and whatever happened inside that cocoon was good for e…….getting fat, losing weight, getting fat, losing weight, it was fine. P4 | Body weight as a protection against the world out there | Positive body image | **Comfort hiding behind fat before** |
| On leaving the “cocoon,” represented by the excess of body fat, there is a feeling of lack of protection and for this reason, at the same time that they achieve this condition of being admired, phobic symptoms appear. | Hard to deal with the attention given after losing weight | Womanliness  A healthy and functioning body | **Effects on security and self-esteem**  **Being attractive** |
| You feel a little more desired... but I haven't learned to cope with this situation yet. P2 | Hard to know how to handle the attention | Womanliness  A healthy and functioning body | **Effects on security and self-esteem**  **Being attractive** |
| Sometimes, it happens that the patient's obesity may serve certain functions which satisfy the needs of the family system, and the patient's weight loss may be perceived by her most immediate environment as an undesirable and threatening phenomenon. In the same way, for some women, partners' jealousy is a new factor, which they are not used to dealing with, and an imbalance is created in the  relationship with their partners, thus jeopardizing the advantages gained by the improvement in the quality of life and by the couple's experiences of resocialization which result from the weight loss and its consequences. | The weight loss leads to a new identity that even changes the dynamic in the family and relationships. Partners can feel that their positions are threatened by the new identity. | Womanliness  A healthy and functioning body | **Effects on security and self-esteem**  **Being attractive** |
| Now my husband has begun to talk like this: Why are you going out? To show off? P3 | Husband has become insecure from the new body appearance | Womanliness  A healthy and functioning body | **Effects on security and self-esteem**  **Being attractive** |
| After some time, some patients are still discontent with their bodies. The same issue of shame that had previously  been attributed to obesity is now attributed to flaccidity, skin folds, and scars. | The extra skin makes them feel ashamed and is a reminder of past self | Positive body image | **But the loose skin makes them ashamed** |
| Now I'm no longer fat but I have flab, loose skin and everybody looks at me the same way. P6 | People look at them in the same way as when they were obese due to loose skin | Positive body image | **But the loose skin makes them ashamed** |
| These marks of obesity are elements that strongly contribute to the frustration of their expectations of once again having a beautiful, healthy, and functional body. | Past is hindering positive experiences of the new body | Positive body image | **But the loose skin makes them ashamed** |
| When I'm dressed, I'm no longer ashamed, you know... Now, without my clothes on, that's another story. Because I'm all flaccid. I'm ashamed. P5 | Ashamed of showing a naked body because of all the lose skin | Positive body image | **But the loose skin makes them ashamed** |
| Due to this perception, the risk of isolation is great, demanding special attention from the psychological team. | You need help to be able to adapt to all changes | Balancing body and mind |  |
| I'm withdrawing....Before it was because I was obese, now am I withdrawing because I'm thin? P4 | Isolation because of feelings of not fitting in | Balancing body and mind |  |
| **9 Paul et al 2022** |  |  |  |
| Women experienced their obesity as the main reason for not being able to have children. They described how they were concerned about the effect of weight on fertility and the capability of carrying a foetus safely to term. They also expressed concerns that obesity could affect their pregnancy outcomes | Obesity is hindering pregnancy and having a baby | Womanliness  A healthy and functioning body |  |
| It’s not a good combination to be extremely overweight and want a baby (W7). | Obesity is a hinder to having a baby | Womanliness  A healthy and functioning body |  |
| My weight was the reason I could not move forward with my future plans on having a family (W1). | Being overweight is hindering fulfilling the dream of a having a family | Womanliness  A healthy and functioning body |  |
| Women felt that the consequences of their excess weight became tangible when they experienced irregular periods and a lack of ovulation. There were concerns that they may have damaged their bodies beyond repair | Obesity is damaging womanliness and fertility | Womanliness  A healthy and functioning body |  |
| I wasn’t sure that I could produce that hormone again; I was afraid that my body was so destroyed from being overweight (W3). | Obesity is damaging womanliness and fertility | Womanliness  A healthy and functioning body |  |
| I weighed a lot, my ovulation did not exist, my  menstruation was very irregular and very heavy (W14). | Obesity was affecting hormones and cycles | Womanliness  A healthy and functioning body |  |
| Many of the women struggled to become pregnant and felt disappointed about not being able to see a positive pregnancy test. They described a feeling of resignation to never being able to have a child of  their own: | Obesity is damaging womanliness and fertility | Womanliness  A healthy and functioning body |  |
| We tried and tried and tried but we never got a positive result (W10). | Obesity hindered pregnancy | A healthy and functioning body |  |
| I tried over and over to lose the weight, but I always gained it back again (W19). | Several tries to lose weight but no control overweight-JoJo | Awareness of feelings and needs |  |
| Bariatric surgery became the definite solution for the participants, and many were introduced to the surgery by infertility specialists, gynaecologists, dieticians,  and general practitioners: | Surgery was the recommended option | A healthy and functioning body |  |
| So, I went to a fertility specialist and gynaecologist with a private practice, and she told me to seek gastric bypass surgery because she had seen such good results in her patients (W9). | Surgery was the recommended option improving fertility | A healthy and functioning body |  |
| I met this incredible general practitioner who listened to me, and my sorrows and he explained that the gastric bypass surgery could be a great solution for me to lose weight, improve my fertility and health and be able to have children (W13). | Surgery was the recommended option for fulfilling dreams of having a baby | A healthy and functioning body |  |
| A few participants expressed feelings of guilt and shame over their situation. They perceived themselves as selfish for causing the weight gain and for seeking the surgery to have children: | Feel guilt since they caused this obesity and now use social funds to take the easy way out-surgery | A healthy and functioning body |  |
| I felt like a failure in that area of my life (building a  family) …. how can I find it more important to eat  candy and chocolate than to be able to have a  baby? (W13) | Choosing sweets before having a family—bad decision | Awareness of feelings and needs |  |
| There are so much more important things going on in the world, children that are starving, fighting  cancer…why should I get this surgery to have a  child (W3). | Guilty conscience about the surgery |  |  |
| Almost all participants lifted their concerns about obesity as a hindrance to leading an active and engaging life with their children: | Obesity is a hindrance to be a good parent | A healthy and functioning body |  |
| I want to be able to give my child the life I always  wanted; having overweight parents is an invisible  handicap (W1). | Want surgery to be able to be good parent | A healthy and functioning body |  |
| If I don’t have the energy to deal with myself, how am I going to be able to deal with a child (W7). | Obesity is a hindrance to be a good parent | A healthy and functioning body |  |
| Most of the interviewed women had, after surgery, become pregnant spontaneously and carried the child successfully to birth. Others expressed relief to be able to be accepted for in vitro fertilisation. They expressed  joy, happiness, and gratefulness to have been able to reach their goal: | The surgery enables becoming a parent | A healthy and functioning body |  |
| I am so grateful and glad for everyday that I have with my son (W13). | The surgery enables becoming a parent | A healthy and functioning body |  |
| I felt that now I am in the club, when my BMI started to drop downwards, now I knew that we could get the IVF help and put ourselves on the waiting list. The fact that I got pregnant is completely dependent on doing the  surgery (W9). | The surgery enables becoming a parent and joining the social group—parents | A healthy and functioning body |  |
| I am very happy that I did the surgery and know that now I can become pregnant naturally (W7). | The surgery enables becoming a parent | A healthy and functioning body |  |
| Almost all participants talked about their perceived experience of changes in hormone levels. They explained the difficulties they had prior to surgery with irregular menstruation, lack of ovulation and extreme bleeding when they did have their periods.  The majority described a change that coincided with the weight loss as they experienced normalisation of their menstrual cycles and a reduction in hirsutism.  Many described an immediate ‘restart’ of their menstrual cycles with punctual periods and ovulation: | The weight loss led to having normal hormones and regaining womanliness  Normal periods were a sign of a normal hormone cycle | Womanliness  A healthy and functioning body |  |
| It’s like my body got a restart hormonally (W9). | Hormones were normalized | A healthy and functioning body |  |
| With my PCOS I had my period maybe two or three  times a year, now I have it regularly once a  month (W13). | Hormones were normalized- regular periods | A healthy and functioning body |  |
| Many women also described a lack of femininity when they were obese, with male-pattern fat dispersion, facial hair, and acne. The weight loss was described as a return to feeling and looking like a woman again: | The weight loss led to having normal hormones and regaining womanliness | Womanliness  A healthy and functioning body |  |
| Before I felt that I had a lack of female hormones, I felt that nothing worked like it should and now when everything works, it feels like a whole new world, I feel like a woman now (W9). | The weight loss led to having normal hormones and regaining womanliness | Womanliness  A healthy and functioning body |  |
| Participants conveyed several positive elements of the postoperative period. They experienced changes in their everyday habits, and they could now ambulate without the hindrance of their excess weight. They  also felt a relief that they no longer had to reflect on the size of a chair or if a clothing store would have their size. Several participants did express cynicism and shock over what they felt was an extreme superficiality in society, as well as patronising attitudes among healthcare professionals: | Able to function properly since losing weight and easier to move  Bothered that people around treat people differently due to weight | A healthy and functioning body  Positive body image |  |
| I had to adapt to my new shell even though I was the same person on the inside, the people around me, their focus changed towards me, their way of talking to me, socialising with me, it was a bit tough to get used to (W5). | It’s hard to accept how superficial society really is | Positive body image | **How people treat differently depending on bodily appearance** |
| It’s really sad because I can also think the same way sometimes, and its society’s socialisation that has made us this way (W4). | It’s hard to accept how superficial society really is | Positive body image | **How people treat differently depending on bodily appearance** |
| Everything is your fault when you are overweight (W6). | Obese is your fault | Positive body image |  |
| When they see an obese person, they automatically think that this person has problems (W13). | Obesity is a sign of bad character | Positive body image | **How people treat differently depending on bodily appearance** |
| If you stub your toe then it’s because you are  overweight, whatever problem I had, whether physical or psychological, it was always my weight that was the cause (W3). | Obese is your fault and the cause of all your problems | Positive body image | **How people treat differently depending on bodily appearance** |
| Before I would always avoid plastic lawn chairs, since I knew they couldn’t hold my weight. Now I can sit anywhere, and I don’t even reflect on it anymore (W14). | Losing weight enables physical function and free moving | A healthy and functioning body |  |
| I can do more now, I am more flexible and more  physically active, I can move and run and do things I could not do before (W8). | Losing weight enables physical function and free moving | A healthy and functioning body |  |
| **10 Young et al 2013** |  |  |  |
| their changing weight shapes a shift in their sense of themselves in the world. | Losing weight gives a changed perspective on themselves | Positive body image |  |
| . . . but tonight I just feel a new sense of confidence about myself and I walked around  with my head held high. And I’m still a big girl. I just felt different and I felt like I belonged so not only is it physical things that have changed for me but emotional things too. (Divataunia, 3 months post-surgery) | Losing weight makes changes in body and mind | Balancing body and mind |  |
| I was uncomfortable being fat but that doesn’t mean that once you’re not fat you’re  comfortable being skinny, it’s a totally different thing . . . . I guess I didn’t anticipate that. I always thought that as soon as I’m skinny I’ll feel exactly how I always wanted to feel and I’ll feel beautiful and confident and I won’t have to worry about people looking at me or me looking at myself in the mirror and feeling shitty or whatever.  Some of that’s true, I feel good when I look in the mirror. (Thebandinme, 3 months post-surgery) | Obesity gives unpleasant feeling about body appearance-- womanliness | Womanliness  A healthy and functioning body | **Effects on security and self-esteem**  **Being attractive** |
| As their bodies change, so too does their sense of self, as illustrated in the following section. | Bodily changes also affect the sense of self- as a woman | Womanliness  A healthy and functioning body |  |
| But you look at yourself after losing 20 pounds and you’re like god I’m still fat, I still look at myself and see myself fat. And I can see the difference, I can see 20 pounds off of me but I still see a fat person. (14 days post-surgery) | Obesity affects your mind of experience body image | Balancing body and mind |  |
| I just had this sort of wall up and it was really protective, that layer of fat protective and now its melting away its making me feel really scared, like vulnerable and sensitive and so I’m trying to deal with that. (Thebandinme, 5 months post-surgery) | Obesity was a protection | Balancing body and mind |  |
| Despite the desire to lose the fat and the fat sense of self that accompanied it, its loss does not necessarily immediately, or inevitably, produce a fresh or less complicated sense of who they are, nor what they may desire to become. | Bodily changes also affect the sense of self- as a woman  What dreams are there in future | Womanliness  A healthy and functioning body |  |
| Like when I see fat girls I’m like what’s up ‘cause you’re my people. Like there’s this really fat girl in my maths class and I just want to hang out with her and talk like fat girl stuff! But then I feel like a fucking traitor because I was able to have surgery and lose a bunch of weight and now I’m not like obese anymore. I just feel like I betrayed  them kind of. (Thebandinme, 9 months post-surgery) | Guilty conscience about the surgery  Bad feelings because there are other women still obese | Balancing body and mind |  |
| Part of me is sad, especially as I had younger teenagers coming up to me telling me that  I was a role model, which was amazing, you know part of me felt a responsibility to that,  to be somebody who was strong. Because not everybody’s a size 2 . . . . but I’m embracing  a healthier lifestyle and that’s what I need. (Divataunia, 13 days pre-surgery) | Responsibility to take care of the new body  Being a role model | A healthy and functioning body |  |
| Hey YouTube, it’s been so long since I’ve talked to you and I’m really sorry. I’ve been getting mail from everybody checking in with me which is really nice to know you guys are still interested in what’s going on with me. (Thebandinme, 9 months post-surgery) | Other people interested in how it is going | A healthy and functioning body |  |
| I just want to thank everybody, the best support and information I’ve gotten has been here on YouTube and I feel really thankful that this is a resource I’m able to use. Not only that, but just meet some really great people, you all are fantastic really and I mean that. (Divataunia, one week pre-surgery) | Other people interested in how it is going--supportive | A healthy and functioning body |  |
| It would seem for the women, losing weight translates to a loss of networks that  afford significant resources, support and sense of community to women such as the  fat acceptance movement and the big beautiful women movement//in a paradoxical manner, the focus on image and fat acceptance reduces women’s being to that of ‘‘fat woman’’. Whether she is a proud fat woman or not, this is a limited way to understand human subjectivity | Obesity seems to delete body image to something fat not seeing womanliness | Womanliness  A healthy and functioning body |  |
| And how do I, how do I identify myself now? I don’t think I can be classified as a BBW anymore but I’m also not thin. I wish I didn’t have to have a label. But when you do personals online you have to say what your body type is. Uhh I hate it, I hate  that you have to give a description. (5 months post-surgery) | Hard to find your identity | Balancing body and mind |  |
| . . . all these people see me and I’m sure they just look at me like anybody else, you know like that’s just whatever like another slightly overweight girl and that’s really weird to me to just be another one of those girls. I don’t feel like another one of those girls, I still feel like a fat girl. (9 months post-surgery) | Losing weight makes changes in body and mind -still obese | Balancing body and mind |  |
| . . . little changes every day are happening, they add up, they’re overwhelming, they’re  wonderful, sorry I’m going to start to cry again, but it’s tricky. It’s tricky to figure out who you are, not just physically but emotionally and personality wise. Your whole life changes. (Divataunia, 8 months post-surgery) | Hard to find your identity  Changes both physical and emotional | Balancing body and mind |  |
| What does it mean to you that’s like eating food, losing weight, being thin or being fat.  How do you relate those things to who you are as a person, who you think that you are, and what you think you’re worth, and where you’re going. It’s weird, like before I was like oh I’m fat, lazy, that’s pretty much all I’ll ever be and so hmm I’m no prize, you know I don’t deserve a lot. And now it’s like wow I don’t have to be like that.  I can go to school, and get a really good job, and have a stable relationship with someone I’m really crazy about and really attracted to, and have a cute little life and money, and self-confidence, and look cute and be attractive. Like holy shit that’s a lot of stuff to all of a sudden have to think about. Where before it was so easy to be like,  meh I’ll never get there. So I think that’s why I react so emotionally to these little things because it’s like before I didn’t have to deal with them, I could just be like I’m fat, I need to diet and let that rule my life and my thoughts, like my thought process never got passed that. It was like oh if I could only lose weight. Well now I have and  now what? . . . . I like taking time like this for myself to think about what’s going on, and where I’m at and who I am. (Thebandinme, 9 months post-surgery) | Hard to find your identity  Losing weight makes you able to be physical active and live a good life- have a relationship | Balancing body and mind  Womanliness  A healthy and functioning body |  |
| An outcome of this ‘identity crisis’ is the creation of a new self. Divataunia and  Thebandinme regularly refer to and reflect on their ‘new’ and ‘old’ selves in a variety of ways. Divataunia speaks of ‘who we become after the surgery’ (14 months post-surgery) and how she is ‘mentally having a really hard time balancing my new life and my new struggles but with my old bad habits and demons’ (22 months post-surgery). | Hard to find your identity  Balancing your new life with past habits | Balancing body and mind |  |
| This notion also resonated with hebandinme, ‘Us bandsters have a wonderful new life to enjoy’ (5 months post-surgery), mentioning ‘I’m a totally different person now than I was a year ago’ (6 months post-surgery). | Losing weight makes you a new person | Balancing body and mind |  |
| So this is sort of a goodbye to my overweight self. And a year from now I can look back and see what a transformation it’s been . . . . I just want to try and document everything I can for my view back on this journey. (13 days pre-surgery) | Losing weight makes you a new person | Balancing body and mind |  |
| . . . my ego is swelling a little bit but also at the same time I don’t know who I am, I don’t know if I feel good about myself but I guess that is the journey and I know a lot of people consider lapband surgery as a journey and an emotional and personality type journey where it really changes you and it’s hard to anticipate how it’s gonna  change you and who you’re gonna end up being. (Thebandinme, 2 weeks post-surgery) | Hard to find your identity | Balancing body and mind |  |
| I have control over what goes in my mouth and what happens with my body now and that’s the best thing that’s happened from this surgery besides the weight loss. (Divataunia, 5 months post-surgery) | The surgery enables taking control | A healthy and functioning body  Awareness of feelings and needs |  |
| That’s the hugest part of lapband that was attractive to me. I get to control how much  I eat, I get to control my appetite, I get to control my weight, I get to control my body, in a way that I’ve never been able to. (Thebandinme, 15 months post-surgery) | The surgery enables taking control- taking control over the body | A healthy and functioning body  Awareness of feelings and needs |  |
| ‘It’s about me and making myself better’  (Thebandinme, 3 days pre-surgery); ‘The more I think about it the prouder I am  of my decision to be proactive in my health and to really take charge of it and change my life for the better’ (Divataunia, 10 months post-surgery). ‘Being better’  explicitly means being able to do a multitude of things they did not feel able to do  previously. | The surgery enables taking control- taking control over the body  Taking responsibility for health and a better life | A healthy and functioning body  Awareness of feelings and needs |  |
| I don’t feel like my body is holding me back mentally or physically anymore. And I still have about 40–50 pounds to go but just having 100 pounds off really makes such a huge difference in my life. In every way, my health, my stamina, my clothing, my self-esteem. (Divataunia, 5 months post-surgery) | Losing weight makes it easier to be active- both physical but also mentally | A healthy and functioning body |  |
| Yes I think I have already succeeded, I think the question is, can I maintain it? And that is where the struggle comes in . . . . There’s not anything that makes me sick. And that is good, but it’s also bad because it could lead me back to old behaviours of my past. (Divataunia, 8 months post-surgery) | Balancing your new life with past habits | Balancing body and mind  Awareness of feelings and needs |  |
| It’s not easy, it’s not pretty, there’s lots of struggles, people still with the issues that are up  here [points to head] even after they’ve lost weight. And then there’s, like how do I feel  about my body now that I’m skinny, am I ok with it now?And relationships, and anxiety,  and emotional eating. (Thebandinme, 5 months post-surgery) | Hard to find your identity- bodily changes but what is in mind  Pleased enough to think about relationships | Womanliness  A healthy and functioning body |  |
| Questions, including how do I maintain my  new body, what does it mean for me and the way I relate to other people, must be  addressed. Corporeal changes are not enough to ensure a subjectivity that works  for them, psychological shifts are required too. | New experiences having a new body, relate to others and how people react to their feminine appearance | Womanliness  A healthy and functioning body |  |
| I’m missing the person that I was before surgery. I know I’m still that person in a lot of ways but losing all that weight throws you for a loop you know. (Thebandinme, 9 months post-surgery) | Hard to find your identity- | Balancing body and mind |  |
| I want to date and be this new person. I think that’s the struggle in maintaining a  relationship, you feel like you got a second chance on life. You just reinvented yourself and you reinvented what you want, what you want out of life, what you expect from other people, what you expect from yourself. I think all of that stuff is different for me than a year ago. (Thebandinme, 1 year post-surgery) | Wants to date and get into relationship  New experiences having a new body, relate to others and how people react to their feminine appearance | Womanliness  A healthy and functioning body |  |
| So I was watching my surgery videos and just how goofy, and sort of funny I was being. And I was like, I kind of miss fat Ashley . . . . But watching me before surgery I seemed a lot more jolly. I know that was kind of facade because really I was really  fucking depressed and just like I was excited to be having surgery but in a lot of ways too when you’re fat you don’t have to worry about all the people looking at you and holding you to certain standards, or like getting attention from certain people you might not want . . . . Before I could just be loud and boisterous when I want to be . . . . I got to like be assertive when I wanted to be assertive, I didn’t have to care about being some pretty um super feminine girl, I just worried about being funny and cool and fun. (9 months post-surgery) | Obesity was a protection  Changed body and people around put on demands—being a woman | Womanliness  A healthy and functioning body |  |
| When I was 300 pounds my skin fit me, and it wasn’t like I fooling anybody, I was fat,  that’s just the way it was . . . . But now that I’ve lost 110 pounds I’m starting to see the  effects of that in my body and I’m not liking it at all. And I’m having a lot of insecurity issues which are driving me crazy because I’m turning into someone I don’t like.  I’ve always been very confident and I’ve always been completely in control of my feelings about myself and I’m not now. I’m insecure about a lot of things, most importantly the way I look and the way people respond to the way I look.  (Divataunia, 6 months post-surgery)  Partner: I think that your upcoming changes will only have a positive effect on your  view of yourself, your confidence and everything | All lose skin is disturbing  The new body’s appearances give insecurity and people’s reactions increase that | Positive body image | **But the loose skin makes them ashamed**  **How people treat differently depending on bodily appearance** |
| Thebandinme: You think that, but a lot of people get really freaked out by it. Like people that were fat that had lapband surgery and like noticed that while they’re losing weight and after they’ve lost weight people are like way nicer to them and  like treated them totally differently, and give them more opportunities and stuff like that and they’re just really disturbed by the sort of prejudices that fat people are faced with every day. And it’s true, there’s like so many pre-conceived notions and just the way that people approach fat people, it’s weird. Sometimes it can be really traumatising  to get skinny . . . . They’ll always think that they need to be thinner, they’re still fat, blah, it’s Body Dimorphic Disorder. (Thebandinme, 2 months post-surgery) | It’s hard to accept how superficial society really is | Positive body image | **How people treat differently depending on bodily appearance** |
|  |  |  |  |
|  |  |  |  |
