# Supplementary material for: Impact of bariatric surgery on premenopausal women’s womanliness: A qualitative systematic review and meta-synthesis
Source: PLoS One. 2024 Aug 29;19(8):e0308059. doi: 10.1371/journal.pone.0308059 (PMC11361607; doi:10.1371/journal.pone.0308059)
Supplement: S3 Dataset — (DOCX) [file pone.0308059.s007.docx]

Group the different fusions—present their content—using the texts from each article—write out the content and thus define the fusion's content and classification

**Womanliness Appearance, sexuality, fertility**

Independently of having previous experience of not being obese, the participants expressed how they wanted to return to normality, describing obesity as an obstacle to moving forward with their lives and having a family: (2)

I can go to school, and get a really good job, and have a stable relationship with someone I’m really crazy about and really attracted to, and have a cute little life and money, and self-confidence, and look cute and be attractive. Like holy shit that’s a lot of stuff to all of a sudden have to think about. Where before it was so easy to be like, meh I’ll never get there. So, I think that’s why I react so emotionally to these little things because it’s like before I didn’t have to deal with them, I could just be like I’m fat, I need to diet and let that rule my life and my thoughts, like my thought process never got passed that. It was like oh if I could only lose weight. Well now I have and now what? . . . . I like taking time like this for myself to think about what’s going on, and where I’m at and who I am. (Thebandinme, 9 months post-surgery) (10)

Participant 159: “I do notice that my appearance has become more important to me. I regularly go to the beautician, nail studio, hairdresser, and pedicurist. . . . Now that my feminine shape is reappearing, clothes have become more important.” (1)

Participant 43: “There is nothing wrong with my libido, but it is sometimes difficult to surrender to it because I don’t find my body beautiful. . . . My husband has remarked that he was bothered by my body.” (1)

In contrast, almost a third of women described increased appearance evaluation and investment post-surgery, such as enjoying clothes shopping and feeling more satisfied with their appearance. Almost a third described social freedom and confidence and felt more accepted in social gatherings. (1)

Many women described barriers to sexual pleasure—for example because they worried their partner would dislike their body. (1)

Participant 152: “Before surgery, I very much kept to myself. I think I tried to build a cocoon around myself so that others could not touch me. The more weight I lose, the more I lose my insecurity, and I talk and laugh more. My cocoon has really disappeared.” (1)

Well, yes, if you consider the love part,” I think it could affect me in a positive way, hopefully then, erh . . . since I don’t have a lot of people around me, but the ones I have are very close, erh, and it might also affect friendships positively. Because it feels like I have lost many since I myself have withdrawn. (Participant 9) (2)

I want to get back on my feet first and I want to be comfortable with my body before I . . ., I look at it this way, if I’m not content then I can’t be a role model to my child. . . . Because they see more than you think. I’d rather be done there. I am dreaming of having children, but it’s just not now. (Participant 8) (2)

Like, I’ve always had this ideal body, that I don’t want to be super skinny, not at all, but a bit chubby, like, still having the curves. I don’t like to be the way I am now, for example, that I’m like, overweight. . . . I’ve got an ideal body. It’s just hiding, somewhere in here, right now. (Participant 3) (2)

The majority described the stigma of being obese, and that they already “knew” what others were thinking about them. This was holding them back from going out and building new relationships, which led to isolation. Losing weight was considered as a means to be more outgoing, being able to be more open to new relationships: (2)

Then, when I said that I had applied for this kind of operation, he let this one slip: “but then you’re gonna . . ., then you might dump me when you become . . .” and I just; “when I become what?,” I said. “Don’t finish that sentence now cause then you’ll get a hell, so to speak. Do you think I’m with you because it’s like, some sort of a consolation prize.” (Participant 1) (2)

Although nobody reported that they had received any explicit negative comments on their bodies in sexual content, sexual desire was inhibited by their own thoughts of how their bodies would look in a sexual situation: (2)

And about sex life and so on . . ., it’s ugly, you must have a nice body. (Participant 11) (2)

Losing weight was described as a means to get more confident in a sexual situation, and they were hoping that they would be more relaxed in their sex life: (2)

Better. I mean I think it’s gonna . . ., I mean I think . . ., ‘cause I think . . ., I think that then you might dare to take more initiatives, too. If you’re comfortable with your body, then I guess it’s more that you take what you want. So, I suppose it’s gonna . . . Because it’s like that, that if you’re feeling self-confident then everybody else is noticing as well. (Participant 12) (2)

There was a knowledge that obesity was damaging fertility and the ability to have children. There was hope that the surgery would improve this. (2)

Achieving pregnancy was a positive side-effect of the surgery (2)

Weight loss affects fertility, and hopefully, the surgery enhances natural cycles (2)

Hormones cause weight gain, and weight loss will improve fertility (2)

Most of the participants pointed out the irregular menstruations caused by obesity as the greatest obstacle to getting pregnant: (2)

. . . since I have not succeeded in getting pregnant and I think it depends a lot on my weight. (Participant 7) (2)

Several of them also had knowledge about the negative effects of obesity on pregnancy and mentioned the increased risk of miscarriage: (2)

No, but really, you never know why you have a miscarriage. That is . . ., it’s like you could never really know, but I’m also aware that you’re more likely to have a miscarriage and so on if you’re overweight. (Participant 12) (2)

And then I and my partner want to have children. Then first you must live together for a year, I think it is, it used to be two, I’ve heard. So, we can apply for it now, but it’s no use trying to apply since I’ve got to get below BMI 30 to have an insemination or IVF. (Participant 4) (2)

All participants described a wish to have children in a more, or less, close future, and that having a family was very important to them. None of them had heard anything negative about pregnancies after bariatric surgery: (2)

No. And I know that it really doesn’t stop you from getting pregnant. . . .So that’s why I don’t consider it a problem. Because I know that you still can get pregnant. Yes. . . . Because otherwise . . ., like if I couldn’t get pregnant . . . then I’d never have the surgery. Because that’s my biggest dream in life. That’s just having children. So . . .

(Participant 12) (2)

The participants knew that obesity causes high-risk pregnancies and that this meant a risk for the mother as well as the child. Participants also mentioned that pregnancy could lead to extra weight gain, which might put them in an even worse situation: (2)

None of the participants was worried that bariatric surgery would affect future pregnancies negatively. Some of them were reflecting on the possibility that the foetus would suffer from lack of nutrients, but the fact that friends and family members who already had gone through the operation had delivered successfully afterwards was encouraging enough: (2)

Shortly after surgery, several of them had met a (new) partner, and the ones that had already been in a stable relationship before surgery had gotten married or were planning to marry. (3)

“Definitely. I’ve found my other half now. I have. So that’s a lot. He’s comfortable with me, and I’m comfortable with him. He’s not judging. You can notice that he likes me and the way I look.” Participant 8 (3)

Most of the participants described a more active sex life, which was also more satisfying than before surgery. Internal factors, such as being more comfortable in a sexual situation and enhanced self-esteem, allowed them to demand more of their partners. (3)

Being comfortable with the body and being naked, led to more intimacy. Sex was also a lot more enjoyable when one was able to let go of the thoughts of how one’s own body might appear. (3)

“And there was definitely no talking about positions before. One didn’t dare to, of course, but now I’m not uncomfortable at all, like feeling that I have to sit and hold my belly, or that when you lean forward, you have to hold everything in place “- No, you can’t look there”” Participant 8 (3)

The enhanced self-esteem made it easier to make demands. The participants talked about feeling relaxed about guiding the partner to better sex, and the stimulation needed to reach climax. (3)

“. and then not being afraid of saying what you want and so on. So just,. Really, to be comfortable with yourself leads to a thousand other things around sex that makes it a much, much better experience and makes it more pleasant, and makes it, like, easier to have orgasms.” Participant 10 (3)

The participants described how they were now much more satisfied with their own bodies and appearance. Self-esteem felt improved, and inhibitions were lowered. (3)

Several participants described increased desire and a more active sex life. They pointed out factors such as increased energy levels and endurance, which made having sex more interesting. (3)

Another participant pointed out less need for lubricants as the cycle had become more regular and ovulatory. Yet another said that the weight loss had increased “the feeling of having intercourse.” (3)

“I had some difficulties getting wet before, since my ovulations started again, we haven’t needed much lubricant at all.” Participant 4 (3)

One of the participants had already become a parent, and a second was pregnant. The other participants said that they wanted to have children in the future, but not all of them felt ready to get pregnant yet. Having regular cycles was considered very positive, as a marker of female fertility. (3)

Most of the participants now had a regular cycle and expressed their joy at feeling like a normal woman again. They talked about feeling relieved as the body was working as it was supposed to again. For the participants who wanted to conceive now, ovulation was very important. (3)

“And people are complaining about their..., I love my period.” Participant 4 (3)

Some of the participants had gone through surgery to enhance their fertility and were now trying to conceive. Another 2 had already got pregnant when they had met a partner. All participants were planning for children in the future. (3)

“I hope., so before., I haven’t got pregnant before, but I was rather thinking that I can’t get pregnant, but I’m still hoping that I actually could. I really would like to have a family at some point.” Participant 3 (3)

We also found the positions: ‘my family’ ‘my partner’ and ‘my work’ were significant, while after the surgery, the relational component becomes even more important. (4)

Women described relationship problems caused by their weight. They mentioned the feeling of distance, deterioration of physical intimacy, and restriction of their social lives. (5)

Some of the women stated that relationships with their spouses worsened due to being overweight, and some described the untold emotional distance between them. A woman stated that she decided to have surgery only because her husband cheated on her moreover; another woman reported that she was planning to divorce after the surgery (5)

My priority was my health, my marriage was so important to me, but I have tried to break down my marriage many times because my husband was bothering me then. I didn’t want him, I was repeatedly saying that “I will divorce”, “After the surgery, I will divorce that man!” (P14, Age 42 (5)

The women described the physical distance between her and her husband due to snoring and sweating at night. In some cases, it was women who wanted to put some physical distance, such as having separated their beds, to avoid sitting next to him or trying not to be seen by their husbands however, in some cases, the husband verbally demanded staying far or not being together with her (5)

My physical condition was influencing our relationship… Normally we do not touch each other. He and I are feeling uncomfortable… He was keeping himself at bay, feeling estranged from me… (P1, Age 35) (5)

For example, that is, my husband always wanted me to lose some weight, well, he would say, “You are overweight, you are not matching me when we go somewhere together”… After all, I was sleeping apart from him, I didn’t want him to see my body since I would snore, even all the building would hear me, not only my husband, therefore, I would get embarrassed… (P14, Age 42) (5)

Limitations in social life may result from women’s concerns about their appearance. Some women expressed their reluctance to go out with their husbands and friends because they could not find an outfit that suits well with them, and they did not like what they could find to dress. Although some women did not report any lived experiences with their husband, another concern that prevents women from going out is the fear of bad smells to their husbands and people around them (5)

Nobody would like to sit near you. I sweat a lot. Whenever someone sits next to me, I ask myself, “Do I smell awful? Is it obvious to the person near me? It was a more social problem, to be honest, I did not have an apparent problem with my husband, I guess he would want to go out and have a thin wife to go out with him because we were not used to going out together. (P16, Age 44) (5)

Women stated that their excess weight caused sexual problems by affecting the appearance of their bodies, physical activities, physical health, sexual functions, and their perspective on sexuality (5)

A few women expressed a decrease in the quality of their sexual lives and romantic relationships with their husbands. This negative effect on their sexual life resulted from different reasons such as lack of self-esteem, feeling unattractive, and body image impairment. Some women described decreased self-confidence and feeling uncomfortable with the idea of bad smell due to sweating which prevents them from having an intimate relationship with their husbands (5)

As I mentioned, I felt humiliated. My husband’s treatment of me, oh no! He was not insulting me, there was nothing verbal, but he was falling asleep right after intercourse, turning his back to me, and then I was feeling abused. I don’t like it, I am a tactful person. Instead of turning your back to your wife, hugging, caressing, or speaking to her would be more motivating. I would like to feel loved, as well. (P13, Age 45) (5)

For instance, I was abstaining from showing my body to my husband while the lights were on. Fat was spilling out from everywhere on my body. (P8, Age 34) (5)

The difficulties experienced by women during intercourse due to excess weight and related problems involved the inability to keep up with their husbands, not being able to move, sweating, and pain. In some women, these difficulties caused struggle to satisfy themselves or their husbands during intercourse or even sometimes made women not willing to have sex with their husbands. (5)

Well, I mean, you cannot raise your foot or leg, you cannot turn on your side, you cannot do anything!… The man thinks that he is sleeping with a robot… (P9, Age 43) (5)

Honestly, we had never had a normal sex life; actually, I think that I deserved this cheating… I was unable to breathe, I mean, I did not want to be with him, I was like pushing the man away. (P6, Age 30) (5)

These are the problems that lead women to have dysfunction in their sexual life and prevent them from experiencing sexual satisfaction. (5)

Women described decreased in sexual satisfaction, lack of lubrication, and inability to reach orgasm or perceived lack of partner’s desire and satisfaction during intercourse. (5)

How can I say, I was unable to orgasm. I mean, I was unwilling… When I was willing, he was not responding, and vice versa, this was the frigidity between us. (P1, Age 35) (5)

A few women stated that they see sexual intercourse as a duty and often sacrifice their satisfaction to please their husbands. Even one woman described sexual intercourse as a ‘death’ in which she does not have any pleasure at all (P15, Age 52). (5)

I mean, I, only see it (sex) as a duty. That is, with regards to married people, we see sex as a duty, seems like we do not have any other responsibilities. I mean, tidying up the house, and cooking the meals have no importance, they are not obligatory, but this is… So, conscientiously I feel like that, every Friday, as a duty, I mean, it is not something that is done because I want to. (P13, Age 45) (5)

Being able to do activities that others can routinely perform, and enjoying a social life with their husbands were women’s expectations from surgery. (5)

Women stated that they dreamed of going out with their husbands after surgery. In addition to the limitation of social activity with their husbands, women also described the negative effect of weight on their social life with their family and friends. (5)

So to take a ride around, to go to a place with my husband, to go to a store, and buy casual clothes, no such big size, there is no ‘not for you’, so you can walk around comfortably with him. (P11, Age 28) (5)

Women stated that they expected to be able to affect their husbands sexually and to improve their sexual life with weight loss after surgery. (5)

Women expressed that they were expecting to feel more satisfied in their sex lives, make their husbands jealous and feel their husbands’ desire much more than before. Most women stated that they desire to make their husbands turn on by their appearance and to have more intimate relationships (5)

Well, for me, sexuality is a very important thing. Because it is the need of my body and soul, I mean. I like having sex… Of course, I had always motivated myself with that dream, “Hey, I will lose weight, and I will drive you crazy!” etc… and I was thinking that his respect for me, which, thank God, already exists, would increase. I mean, of course, like every man, he wants to have a pretty wife nearby. (P7, Age 33) (5)

Some patients reported that the surgery led to improvements in their marital relationships. Most women mentioned that their husbands showed an increased interest in them, including an increase in sexual/ romantic behaviours. (5)

After surgery, the weight loss and body composition change those women experienced made them and their husbands more intimate with each other. Particularly the surgery solved the problems of snoring and sweating that caused to women stay away from their husbands. Also, the surgery helped women be more comfortable, share the same bed with their husbands, and enjoy physical intimacy with them. Some women expressed that their fear was relieved, and they did not show any resistance to their husbands’ intimacy attempts. (5)

When your husband comes home, you always hug and kiss, say welcome and my husband loves it, but I used to always run away. Because I was so sweaty, I could not wear anything, but, now we are like new lovers at the door again. (5)

I mean, we are having our second spring… He always hugged me and wanted to sleep in touch with me, I always ran away. Because when he puts his hands to my body, he touches either my belly or my floppy breast. I am getting more comfortable with him now. (P16, Age 44) (5)

These are the feelings of women about their overall relationship with their husbands. Even a few women mentioned that the surgery saved their marriages by having better sexual life and relationship with partners. One woman stated ‘We did not have a life before this surgery. We were like sisters and brothers. Now, we are a real couple again…’ (P4, Age 44) (5)

We would not chat before, he would not even look at me and my eye, there were quarrels and disputes, but now we look at each other, exchanging glances, chuckling, and having chats afterwards, cackling, I mean, while I am in the kitchen, he is coming and doing certain moves I cannot explain…. He is cheerful,

and I am cheerful, too, many things have changed. (P14, Age 42) (5)

Women expressed that they felt more comfortable going out with their husbands and felt that their husbands were also more comfortable going out with them. After surgery, couples tend to spend more time together and go out in public. (5)

The way he looked at me changed… Before we would never go out to drink a coffee, for example, we would even not share the same room at home… My man is now sitting with me cheek by cheek, holding my hand… (chuckling) (P6, Age 30) (5)

Most of the women stated that an increase took place in the frequency of sexual intercourse, while five patients mentioned that they experienced no change. Women generally felt positive changes in their sexual life; however,

a few mentioned an increase in the amount of foreplay and sexual desire. (5)

Many women described a positive increase in their sexual lives after the surgery. They pointed out that their sexual experiences were different and could not be compared with before the surgery. (5)

Sexual life before the surgery was like death, afterwards, it revived. (P15, Age 52) (5)

I call him, for example… I say, “Let’s do something, come.” I am smiling, he is smiling, too (chuckling). Let me say, I turned out to be bawdy… For instance, when he wants sex, I do not refuse now. We have intercourse whenever he wants now. (P5, Age 39) (5)

Honestly, it is perfect now. (Laughing) I mean, everything is right on track, I can move comfortably. I can move any way I want; I can behave as I like, how can I say, it (sex) is easy and better now. (P2, Age 42) (5)

Honestly, it is quite well. I would have another baby if I didn’t have a tubal ligation… Yes, it is very well now, I used to say to myself that I knew how to experience sexual pleasure… I never experienced pleasure. For me, there was no life before. (P9, Age 43) (5)

In addition to experiencing an increase in sexual desire after the surgery, some women stated that their husbands’ sexual desire also increased, and having more satisfaction due to having more options in movements and motion during intercourse (5)

Well, before, for example, he was not sidling up to me, but now, whenever he sees me… (chuckling) Before, he did not have this much desire. I mean, there was no problem even if I was absent for a month. But now, I am getting caught twice a month. (chuckling) (P5, Age 39) (5)

Yes, in the words of my husband, he says, “Now we are wrestling, but before, it was not like this”. (P12, Age 29) (5)

Although most women reported increased sexual desire and satisfaction, only one woman stated that their sexual life was worse than before. (5)

We did not have a problem before, but right now we have. I have more pain for instance… I don’t want it now. There is dryness and reluctance to engage in sexual intimacy. Even, I cannot be satisfied. (P4, Age 44) (5)

Women stated that weight loss and having a slim physical appearance gave them more freedom in what to wear and increased their self-esteem after surgery. Some women described the desire to look in the mirror and like what they see. (5)

My self-confidence has changed. I mean, I can go out now. I can speak with my friends, I’ve begun to attend social activities, and previously I was making excuses to my friends. (P8, Age 34) (5)

Many women stated that changes in women’s feelings, appearance, and relationship with their husbands and overall positive changes in sexual life led women to feel loved by their husbands again. (5)

Now, I say, I feel stirrings, as if we are having a new love or experiencing it again, there is something, I mean he feels about me… It must be a mutual desire since you feel it from the other side, so you reflect… Well, if I wanted to do it (sex) every day, he would agree. (5)

I mean, we are as if just married. (P14, Age 42) (5)

I mean, there is a big love for my husband. It is something different now, when I wear tights, for example, he recently said to me, “You look so sexy! I will never let you go out in those pants!” It was the first time I heard something like this from him… (P3, Age 35) (5)

Women described their concerns and anxiety related to being overweight and its effect on their relationship decreased after surgery. Women’s moods and demeanour became more pleasant and positive toward their husbands and toward others (5)

Before the surgery, I was a fighter. We had a lot of fights, and after these fights, there was a distance between us. I used to feel as if a stranger had touched me, not my husband. I’ve had problems like that. Right now, I’ve overcome all those problems. (P11, Age 28). (5)

I love myself in terms of appearance. I can wear whatever I want. For instance, (before), I was not able to even tie my shoes normally. (P1, Age 35) (5)

P3: My friends could not give me a hug; I could not stand it…. Before, I didn’t feel my limits, I just knew that people should stay away from me, they should not know anything, and I was just keeping everything to myself. (6)

P1: You feel better; you get approval then…..approval from other men. (6)

P4: Having children, is now a part of my future. (6)

One participant now allowed others physically to get close to her. (6)

Invincibility is metaphorically described as ‘being superwoman’. (6)

Through bodily, social and mental well-being, all participants considered the opportunity of realizing their dreams of having children and raising a family (6)

Participants’ narratives depict improved opportunities in their life, where confirmation from others gave them a sense of equal status. (6)

“I thought that I would solve my problems, that I would be happy, get a boyfriend…” P5 (7)

In the postoperative period, the women have to face new life experiences, such as jealousy, mistrust, fear, and envy that, until recently, had not existed. (7)

“Now my husband has started to talk like this: go out for what, to show off? So, another phase has already started, and I still have not learned how to deal with it…” P3 (7)

After all the effort to find their place in the world again and to be admired, they have faced the reappearance of their feminine bodies: a new situation with which they are unable to cope. (8)

Weight loss after surgery leads to a marked improvement in body image and attractiveness… (8)

You feel a little more desired... but I haven't learned to cope with this situation yet. P2 (8)

Sometimes, it happens that the patient's obesity may serve certain functions which satisfy the needs of the family system, and the patient's weight loss may be perceived by her most immediate environment as an undesirable and threatening phenomenon. In the same way, for some women, partners' jealousy is a new factor, which they are not used to dealing with, and an imbalance is created in the relationship with their partners, thus jeopardizing the advantages gained by the improvement in the quality of life and by the couple's experiences of resocialization which result from the weight loss and its consequences. (8)

Now my husband has begun to talk like this: Why are you going out? To show off? P3 (8)

When I'm dressed, I'm no longer ashamed, you know... Now, without my clothes on, that's another story. Because I'm all flaccid. I'm ashamed. P5 (8)

Women experienced obesity as the main reason for not being able to have children. They described how they were concerned about the effect of weight on fertility and the capability of carrying a foetus safely to term. They also expressed concerns that obesity could affect their pregnancy outcomes (9)

It’s not a good combination to be extremely overweight and want a baby (W7). (9)

My weight was the reason I could not move forward with my future plans on having a family (W1). (9)

I felt like a failure in that area of my life (building a family) …. how can I find it more important to eat candy and chocolate than to be able to have a baby? (W13) (9)

Women felt that the consequences of their excess weight became tangible when they experienced irregular periods and a lack of ovulation. There were concerns that they may have damaged their bodies beyond repair (9)

I wasn’t sure that I could produce that hormone again; I was afraid that my body was so destroyed from being overweight (W3). (9)

I weighed a lot, my ovulation did not exist, my menstruation was very irregular and very heavy (W14). (9)

Many of the women struggled to become pregnant and felt disappointed about not being able to see a positive pregnancy test. They described a feeling of resignation to never being able to have a child of their own: (9)

Almost all participants talked about their perceived experience of changes in hormone levels. They explained the difficulties they had prior to surgery with irregular menstruation, lack of ovulation and extreme bleeding when they did have their periods. (9)

The majority described a change that coincided with the weight loss as they experienced normalisation of their menstrual cycles and a reduction in hirsutism. (9)

Many described an immediate ‘restart’ of their menstrual cycles with punctual periods and ovulation: (9)

Many women also described a lack of femininity when they were obese, with male-pattern fat dispersion, facial hair, and acne. The weight loss was described as a return to feeling and looking like a woman again: (9)

Before I felt that I had a lack of female hormones, I felt that nothing worked as it should, and now, when everything works, it feels like a whole new world, I feel like a woman now (W9). (9)

I was uncomfortable being fat but that doesn’t mean that once you’re not fat, you’re

comfortable being skinny, it’s a totally different thing . . . . I guess I didn’t anticipate that. I always thought that as soon as I’m skinny I’ll feel exactly how I always wanted to feel, and I’ll feel beautiful and confident, and I won’t have to worry about people looking at me or me looking at myself in the mirror and feeling shitty or whatever. (10)

Despite the desire to lose the fat and the fat sense of self that accompanied it, its loss does not necessarily immediately, or inevitably, produce a fresh or less complicated sense of who they are, nor what they may desire to become. (10)

It would seem for the women, losing weight translates to a loss of networks that afford significant resources, support and a sense of community to women, such as the fat acceptance movement and the big beautiful women movement//in a paradoxical manner, the focus on image and fat acceptance reduces women’s being to that of ‘‘fat woman’’. Whether she is a proud fat woman or not, this is a limited way to understand human subjectivity (10)

What does it mean to you that’s like eating food, losing weight, being thin or being fat. How do you relate those things to who you are as a person, who you think that you are, and what you think you’re worth, and where you’re going. It’s weird, like before I was like oh I’m fat, lazy, that’s pretty much all I’ll ever be and so hmm I’m no prize, you know I don’t deserve a lot. And now it’s like wow I don’t have to be like that. I can go to school, and get a really good job, and have a stable relationship with someone I’m really crazy about and really attracted to, and have a cute little life and money, and self-confidence, and look cute and be attractive. Like holy shit that’s a lot of stuff to all of a sudden have to think about. Where before it was so easy to be like, meh I’ll never get there. So I think that’s why I react so emotionally to these little things because it’s like before I didn’t have to deal with them, I could just be like I’m fat, I need to diet and let that rule my life and my thoughts, like my thought process never got passed that. It was like oh if I could only lose weight. Well now I have and now what? . . . . I like taking time like this for myself to think about what’s going on, and where I’m at and who I am. (Thebandinme, 9 months post-surgery) (10)

It’s not easy, it’s not pretty, there’s lots of struggles, people still with the issues that are up here [points to head] even after they’ve lost weight. And then there’s, like how do I feel about my body now that I’m skinny, am I ok with it now?And relationships, and anxiety, and emotional eating. (Thebandinme, 5 months post-surgery) (10)

Questions, including “how do I maintain my new body, what does it mean for me and the way I relate to other people”, must be addressed. Corporeal changes are not enough to ensure a subjectivity that works for them, psychological shifts are required too. (10)

I want to date and be this new person. I think that’s the struggle in maintaining a relationship, you feel like you got a second chance on life. You just reinvented yourself and you reinvented what you want, what you want out of life, what you expect from other people, what you expect from yourself. I think all of that stuff is different for me than a year ago. (Thebandinme, 1 year post-surgery) (10)

So I was watching my surgery videos and just how goofy, and sort of funny I was being. And I was like, I kind of miss fat Ashley . . . . But watching me before surgery I seemed a lot more jolly. I know that was kind of a facade because really I was really fucking depressed and just like I was excited to be having surgery but in a lot of ways too when you’re fat you don’t have to worry about all the people looking at you and holding you to certain standards, or like getting attention from certain people you might not want . . . . Before I could just be loud and boisterous when I want to be . . . . I got to like be assertive when I wanted to be assertive, I didn’t have to care about being some pretty um super feminine girl, I just worried about being funny and cool and fun. (9 months post-surgery) (10)

Although most of them now had a regular cycle, several talked about feeling stressed about fertility and said they still did not feel certain they would conceive when they felt ready. Some had friends who had experience of infertility, while others referred to their own previous difficulties conceiving. (3)

“I: And now you’re thinking more about it (having children) then? (3)

R: Yes, actually. Then this with., yes, but since we tried before to achieve a pregnancy, and I didn’t conceive and so on. Probably I’ve got difficulties with that, I mean.” Participant 6 (3)

This is the feelings of women about overall relationship with their husbands. Even few women mentioned that the surgery saved their marriage by having better sexual life and relationship with partners. One woman stated that ‘We did not have a life before this surgery. We were like sister and brother. Now, we are a real couple again…’ (P4, Age 44) (5)

We would not chat before, he would not even look at me and my eye, there were quarrels and disputes, but now we look at each other, exchanging glances, chuckling, and having chats afterwards, cackling, I mean, while I am in the kitchen, he is coming and doing certain moves I cannot explain…. He is cheerful, and I am cheerful too, many things have changed. (P14, Age 42) (5)

In the past, they had been concerned about impaired fertility (6)

I felt that now I am in the club, when my BMI started to drop downwards, now I knew that we could get the IVF help and put ourselves on the waiting list. The fact that I got pregnant is completely dependent on doing the surgery (W9). (9)

I am very happy that I did the surgery and know that now I can become pregnant naturally (W7). (9)

Most of the interviewed women had, after surgery, become pregnant spontaneously and carried the child successfully to birth. Others expressed relief to be able to be accepted for in vitro fertilisation. They expressed joy, happiness, and gratefulness to have been able to reach their goal: (9)

I met this incredible general practitioner who listened to me, and my sorrows and he explained that the gastric bypass surgery could be a great solution for me to lose weight, improve my fertility and health and be able to have children (W13). (9)

A few participants expressed feelings of guilt and shame over their situation. They perceived themselves as selfish for causing the weight gain and for seeking the surgery to have children: (9)

After some time, some patients are still discontent with their bodies. The same issue of shame that had previously been attributed to obesity is now attributed to flaccidity, skin folds, and scars. (8)

Women in the preoperative group anticipated that their life could ‘magically’ change after the bariatric surgery. They found it difficult to distinguish between real and concrete life experiences that a thin body would allow them to live, from the more profound changes that do not depend on weight, such as those relating to the friendships and relationships with the opposite sex. Some participants of the postoperative group, on the other hand, reported continuing to limit their lives. They imagined that so many experiences would change automatically with the weight loss, but it did not happen, because those experiences were related to their personal selfesteem: (4)

**A healthy and functioning body body, functioning, consequences**

The loss of weight is experienced as a valuable opportunity to recover a place in society. (8)

The latter position (the work) was completely absent in the preoperative group, which may indicate that the person was focused on them before the operation and had no space for anyone else (4)

We were discussing a bit, me and my cousin then and another friend who also had had the surgery, that since you can’t eat that much, I mean I myself, so that the baby can get sufficient nutrition and so on, but since others who had the surgery have managed well, so probably the baby gets sufficient nutrition, it seems so, erh, that’s probably the only thing I’ve really considered . . . actually. (Participant 7) (2)

And then I went to get new birth control pills and then I was talking a bit with the midwife and then she actually said that it would be a danger . . ., that it could be dangerous to both of us, the foetus and me if I got pregnant because of the obesity so to say. So, I guess it would be a lot easier if I lost weight. I really don’t want it to be dangerous either to the baby or me if I get pregnant. (Participant 2) (2)

Most women described the body as valuable in relation to important others, such as seeing one’s children grow up. It is noteworthy that this theme was apparent in each of the three writing exercises, not just the writing exercise that focused on communication with others. (1)

Most women described the body as valuable in relation to important others, such as seeing one’s children grow up. It is noteworthy that this theme was apparent in each of the three writing exercises, not just the writing exercise that focused on communication with others. (1)

Participant 56: “Nowadays, I can easily swim 30 lengths without being tired or out of breath. This gives me an incredible kick.” (1)

Participant 148: “I’ll get a migraine then, for example [when being too busy]. That’s no fun, but it is a function that works for me. I find it difficult to respect my limits myself and this migraine forces me to listen to my body.” (1)

Participant 101: “These functions are important to me because I would lead a very isolated life without these functions, would be dependent on others, and my life would become boring and bleak.” (1)

Participant 45: “Before the operation, I could not experience much fun. I was always tired and not in the mood to go somewhere. Now I have lost 27 kilos. I enjoy going out with my husband and daughter, especially because I am less tired.” (1)

Participant 193: “I have terrible hair loss; my hair has become so thin. My nails grow badly and are fragile. This is despite careful intake of calcium and multivitamins.” (1)

Participant 49: “To see my daughter grow up and to enjoy being with her, to be able to hug her and to hear her say how much she loves me before she goes to sleep at night.” (1)

Participant 148: “And if I get too little sleep, I can function relatively well the next day.” (1)

Participant 49: “A few years ago, an orthopaedist gave me little hope that I could walk after my hip surgery. But I do walk, farther and farther.” (1)

Participant 43: “There is nothing wrong with my libido, but it is sometimes difficult to surrender to it because I don’t find my body beautiful. . . . My husband has remarked that he was bothered by my body.” (1)

Some related this to their recovery post-surgery; others described their body becoming stronger despite not having been well taken care of for many years. A similar number of women described their bodies as a “work in progress.” (1)

They appreciated the perceived improvements they had experienced in their body functionality while acknowledging that there were still changes to be made. (1)

In contrast, almost a third of women described increased appearance evaluation and investment post-surgery, such as enjoying clothes shopping and feeling more satisfied with their appearance. Almost a third described social freedom and confidence and felt more accepted in social gatherings. (1)

Most women compared their current body to their pre-surgery body, even though the writing instructions did not mention making such comparisons. (1)

Most comparisons were phrased positively (e.g., increased stamina) but some were phrased negatively (e.g., worsened taste perception). (1)

Overall, most women were positive about the effects of surgery or described that the positive outcomes weighed more heavily. Almost all women described enjoyment of bodily functions, such as experiencing pleasure and pride from physical activities. (1)

Further, most women described a positive body–self-connection, including the various ways their body communicates to them (e.g., developing headaches when stressed), how they respond to these signals (e.g., resting), and how their body enables them to express themselves and be independent. (1)

Almost half of the women expressed appreciation for the resilience and reliability of their bodies. (1)

Participant 50: “Because I can taste and smell, I can enjoy food but also the smell of freshly baked bread.” (1)

Participant 122: “I am on my way to becoming friends with my body again.” (1)

I want to get back on my feet first and I want to be comfortable with my body before I . . ., I look at it this way, if I’m not content, then I can’t be a role model to my child. . . . Because they see more than you think. I’d rather be done there. I am dreaming of having children, but it’s just not now. (Participant 8) (2)

The participants described a feeling of how life was set on pause since they had become obese. They talked about being inhibited both psychologically and physically, and that this would turn back to normal when they lost weight, described as “the real me” is in there, somewhere: (2)

Like, I’ve always had this ideal body, that I don’t want to be super skinny, not at all, but a bit chubby, like, still having the curves. I don’t like to be the way I am now, for example, that I’m like, overweight. . . . I’ve got an ideal body. It’s just hiding, somewhere in here, right now. (Participant 3) (2)

Better. I mean I think it’s gonna . . ., I mean I think . . ., ‘cause I think . . ., I think that then you might dare to take more initiatives, too. If you’re comfortable with your body, then I guess it’s more that you take what you want. So, I suppose it’s gonna . . . Because it’s like that, that if you’re feeling self-confident then everybody else is noticing as well. (Participant 12) (2)

There was hope that the surgery would improve this (2)

Most of the participants pointed out the irregular menstruations caused by obesity as the greatest obstacle to get pregnant: (2)

. . . since I have not succeeded to get pregnant and I think it depends a lot on my weight. (Participant 7) (2)

Several of them also had knowledge about the negative effects of obesity on pregnancy and mentioned the increased risk of miscarriage: (2)

No, but really, you never know why you have a miscarriage. That is . . ., it’s like you could never really know, but I’m also aware that you’re more likely to have a miscarriage and so on, if you’re overweight. (Participant 12) (2)

The participants knew that obesity causes high-risk pregnancies, and that this meant a risk for mother as well as child. Participants also mentioned that pregnancy could lead to extra weight-gain which might put them in an even worse situation: (2)

And then I went to get new birth control pills and then I was talking a bit with the midwife and then she actually said that it would be danger . . ., that it could be dangerous to both of us, the fetus and me, if I got pregnant because of the obesity so to say. So, I guess it would be a lot easier if I lost weight. I really don’t want it to be dangerous neither to me nor the baby if I get pregnant. (Participant 2) (2)

None of the participants were worried that bariatric surgery would affect future pregnancies negatively. Some of them were reflecting on the possibility that the fetus would suffer from lack of nutrients, but the fact that friends and family members who already had gone through the operation had delivered successfully afterward was encouraging enough: (2)

We were discussing a bit, me and my cousin then and another friend who also had had the surgery, that since you can’t eat that much, I mean I myself, so that the baby can get sufficient nutrition and so on, but since others who had the surgery have managed well, so probably the baby gets sufficient nutrition, it seems so, erh, that’s probably the only thing I’ve really considered . . . actually. (Participant 7) (2)

I’m thinking a bit ahead. My parents really were, like I said, both of them, huge before, but they have lost weight and it took them a life-time so I’m scared that it’s like, going to take a lifetime and then . . . before something happens, and I don’t want that. While I am still young . . ., yes. I still haven’t had children yet and, like, all that stuff. Because they were like, real big when we were . . ., when they had us too, me and my sisters. And then . . ., erh, they haven’t had the energy to be as active as maybe other parents have been and so on and I don’t want that, if I’d be a mum one day. (Participant 10) (2)

“I have to tone the belly there” and then “I’ve gotta buy some boobs.” Greedy, one could say that I am, or you just want to get better and better. I think now, it’s not about., if you think about when you were younger, it was all about being thin and slender, sort of. But now I want, now it’s the muscles that I’m looking for and so on..” Participant 1 (3)

Life had changed; as the body was lighter, things in everyday life got easier. This facilitated exercising but also accepting invitations for social events and new activities involving physical activity—making the participants more outgoing. (3)

Shortly after surgery, several of them had met a (new) partner, and the ones that had already been in a stable relationship before surgery had got married or were planning for marriage. (3)

Another participant pointed out less need of lubricants as the cycle had become more regular and ovulatory. Yet another said that the weight loss had increased “the feeling of having intercourse.” (3)

“I had some difficulties getting wet before . since my ovulations started again, we haven’t needed much lubricant at all.” Participant 4 (3)

“I., well since., because I’ve got this depression I’m not in the mood for intimacy., that is neither intercourse nor closeness at all, really. It feels like I’m rejecting him, which I don’t want him to., I mean, to feel. So, in that way it’s worsening the entire situation, so to say.” Participant 3 (3)

“And people are complaining about their.., I love my period.” Participant 4 (3)

When talking about not being ready to get pregnant, other factors than weight loss were described as important, such as getting to know the partner better or having stable economic circumstances. (3)

“No, not really, since he was not done with his studies and didn’t have a permanent job, err, so we never got that far. We didn’t. Like I told you, these are priorities that you want to be done with before having a family.” Participant 1 (3)

Several of the participants planned to postpone pregnancy until 2 years after surgery, on the advice of healthcare staff. (3)

“Now, I no longer feel like it’s uncomfortable to go exercising among other people, like they’d be thinking “what is she doing” and so on. I can do that. I am much, much more comfortable in social contexts, like I said. Going to birthday parties and, like hanging out with friends and so on, that feels great too.” Participant 10 (3)

My physical condition was influencing our relationship… Normally we do not touch each other. He and I are feeling uncomfortable… He was keeping himself at bay, feeling estranged from me… (P1, Age 35) (5)

Limitations in social life may result from women’s concerns about their appearance. Some women expressed their reluctance to go out with their husbands and friends, because they could not find an outfit that suits well with them and they did not like what they could find to dress. Although some women did not report any lived experiences with their husband, another concern that prevents women from going out is the fear of bad smell to their husband and people around them (5)

Nobody would like to sit near to you. I sweat a lot. Whenever someone sits next to me, I ask myself, “Do I smell awful? Is it obvious to the person near me? (5)

The difficulties experienced by women during intercourse due to excess weight and related problems involved inability to keep up with their husband, not being able to move, sweating, and pain. In some women, these difficulties caused struggle to satisfy herself or their husbands during intercourse or even sometimes made women not willing to have sex with their husbands. (5)

Well, I mean, you cannot raise your foot or leg, you cannot turn on your side, you cannot do anything!… The man thinks that he is sleeping with a robot… (P9, Age 43) (5)

I mean, I, only see it (sex) as a duty. That is, with regards to married people, we see the sex as a duty, seems like we do not have any other responsibilities. I mean, tidying up the house, cooking the meals have no importance, they are not obligatory, but this is… So, conscientiously I feel like that, every Friday, as a duty, I mean it is not something that is done because I wanted to. (P13, Age 45) (5)

Being able to do activities that can be routinely performed by others, and enjoying social life with their husbands were women’s expectations from surgery. (5)

So to take a ride around, to go to a place with my husband, to go to a store, and buy casual clothes, no such big size, there is no ‘not for you’, so you can walk around comfortable with him. (P11, Age 28) (5)

Women expressed that they felt more comfortable to go out with their husbands and felt that their husbands also more comfortable to go out with them. After surgery, the couples tend to spend more time together and go out in public. (5)

We did not have a problem before, but right now we have. I have more pain for instance… I don’t want it now. There is dryness and reluctance to engage in sexual intimacy. Even, I cannot be satisfied. (P4, Age 44) (5)

Women stated that weight loss and having a slim physical appearance gave them more freedom what to wear and increased their self-esteem after surgery. Some women described the desire to look in the mirror and likes what they see in. (5)

My self-confidence has changed. I mean, I can go out now. I can speak with my friends, I’ve begun to attend social activities, and previously I was making excuses to my friends. (P8, Age 34) (5)

Women described relationship problems caused by their weight. They mentioned feeling of distance, deterioration physical intimacy, and restriction their social lives. (5)

P3: My friends could not give me a hug; I could not stand it…. Before I didn’t feel my limits, I just knew that people should stay away from me, they should not know anything and I was just keeping everything to myself. (6)

P1: It was kind of admission of failure that I had to have surgery to lose weight. I just thought so, but on the other hand I also took responsibility for my life and my future. (6)

P5: You’re actually not hungry when you eat. Your brain keeps telling you that you are hungry. The stomach on the contrary is about to burst…and it’s hard to get rid of because your brain was operated on. This need, it’s not just removed in surgery…..There’s such a psychological need, all the time. (6)

One participant now allowed others physically to get close to her. (6)

The participants’ experience of body control may have led to a predictability that was experienced as a new-found energy and freedom, which seemed self-reinforcing through confirmation from others (6)

Through the bodily, social and mental well-being, all participants considered the opportunity of realizing their dreams of having children and raising a family (6)

Participants’ narratives depict improved opportunities in their life, where confirmation from others gave them a sense of equal status. (6)

P2: I’m not so afraid that people will say something about me, because I know that I’m about to be transformed now. I know that I’m doing something for it and then it doesn’t really hit me anymore. (6)

P1: I hope that I manage to stay on the right path and I believe in that. When you have become so pleased with yourself and you know how it would be if you fell back, then…. (6)

P1: I want a normal BMI. (6)

P4: I just felt bad, I began to sweat and it was dumping, I experienced….It was really horrible. I would actually prefer to eat like a diabetic so I can be fairly sure that nothing is happening (6)

P3: You just have to find out how much you actually can eat and what you can tolerate….It has been some challenge navigating, such a labyrinth….One has felt some pain at times because you had to figure out what you could do. But it has not been hard in that sense, just a challenge. I’m about to have managed it. (6)

P1: I’ve been good to feel what my stomach tells. (6)

P3: It is limited. It is ok….It’s just to find out that it’s okay. I do not get more than that….I have no cravings in the same way, not at all. And if I get it, then there is the small bowl. That’s what I need. (6)

P5: It is getting all the things back that you’ve said no to over the years, it is quite amazing. (6)

P2: In the past, it was not fun at all for me to be in the city because I just sat thinking that everyone would think I was fat……I wanted to be there, just not like me. If I just looked different. (6)

P4: Was not together with my friends so much; they began to take a little distance from me. They wanted todo different thing i.e. in the city and being with others. So, I went home and was alone, I ate for comfort….It is my best friend…It is the food. (6)

“…I go by bus just to go through the turnstile; it seems that you ate a chocolate bar from so much happiness”. P6 (7)

The sensation in finding themselves again is lived with great pleasure and relief. (7)

This process of the recovery of identity is lived as a born again, that the patients relate to a new life that begins after being operated. It is a phase that is experienced with a lot of satisfaction. (7)

“After I was operated, I was born again. I was born for a happy life. Before that, I was sad…” P6 (7)

The first source of relief, after postsurgical recovery, comes from a strong sensation of acceptance and social reinsertion. They feel that they are part of a world which they were not a part of. They experience a feeling of genuine happiness. (7)

“…everyone says: wow, how you have changed! Ah, now you are back to how you were before!” P3 (7)

The first source of relief, after postsurgical recovery, comes from a strong sensation of acceptance and social reinsertion. They feel that they are part of a world which they were not a part of. They experience a feeling of genuine happiness. (7)

On leaving the “cocoon,” represented by the excess of body fat, there is a feeling of lack of protection and for this reason, at the same time that they achieve this condition of being admired, phobic symptoms appear. (8)

These marks of obesity are elements that strongly contribute to the frustration of their expectations of once again having a beautiful, healthy, and functional body. (8)

It was as if I was inside a cocoon, there was no outside world and whatever happened inside that cocoon was good for e…….getting fat, losing weight, getting fat, losing weight, it was fine. P4 (8)

It’s not a good combination to be extremely overweight and want a baby (W7). (9)

My weight was the reason I could not move forward with my future plans on having a family (W1). (9)

Many of the women struggled to become pregnant and felt disappointed about not being able to see a positive pregnancy test. They described a feeling of resignation to never being able to have a child of their own: (9)

We tried and tried and tried but we never got a positive result (W10). (9)

Bariatric surgery became the definite solution for the participants, and many were introduced to the surgery by infertility specialists, gynaecologists, dieticians, and general practitioners: (9)

So, I went to a fertility specialist and gynaecologist with a private practice, and she told me to seek gastric bypass surgery because she had seen such good results in her patients (W9). (9)

Almost all participants lifted their concerns about obesity as a hindrance to leading an active and engaging life with their children: (9)

I want to be able to give my child the life I always wanted; having overweight parents is an invisible handicap (W1). (9)

If I don’t have the energy to deal with myself, how am I going to be able to deal with a child (W7). (9)

I am so grateful and glad for everyday that I have with my son (W13). (9)

The majority described a change that coincided with the weight loss as they experienced normalisation of their menstrual cycles and a reduction in hirsutism. (9)

Many described an immediate ‘restart’ of their menstrual cycles with punctual periods and ovulation: (9)

It’s like my body got a restart hormonally (W9). (9)

With my PCOS I had my period maybe two or three times a year, now I have it regularly once a month (W13). (9)

Participants conveyed several positive elements of the postoperative period. They experienced changes in their everyday habits, and they could now ambulate without the hindrance of their excess weight. They also felt a relief that they no longer had to reflect on the size of a chair or if a clothing store would have their size. Several participants did express cynicism and shock over what they felt was an extreme superficiality in society, as well as patronising attitudes among healthcare professionals: (9)

Before I would always avoid plastic lawn chairs, since I knew they couldn’t hold my weight. Now I can sit anywhere, and I don’t even reflect on it anymore (W14). (9)

I can do more now, I am more flexible and more physically active, I can move and run and do things I could not do before (W8). (9)

Participants conveyed several positive elements of the postoperative period. They experienced changes in their everyday habits, and they could now ambulate without the hindrance of their excess weight. They also felt a relief that they no longer had to reflect on the size of a chair or if a clothing store would have their size. Several participants did express cynicism and shock over what they felt was an extreme superficiality in society, as well as patronising attitudes among healthcare professionals: (9)

Everything is your fault when you are overweight (W6). (9)

When they see an obese person, they automatically think that this person has problems (W13). (9)

If you stub your toe then it’s because you are overweight, whatever problem I had, whether physical or psychological, it was always my weight that was the cause (W3). (9)

Participants conveyed several positive elements of the postoperative period. They experienced changes in their everyday habits, and they could now ambulate without the hindrance of their excess weight. (9)

I tried over and over to lose the weight, but I always gained it back again (W19). (9)

Some of that’s true, I feel good when I look in the mirror. (Thebandinme, 3 months post-surgery) (10)

As their bodies change, so too does their sense of self, as illustrated in the following section. (10)

Part of me is sad, especially as I had younger teenagers coming up to me telling me that I was a role model, which was amazing, you know part of me felt a responsibility to that, to be somebody who was strong. Because not everybody’s a size 2 . . . . but I’m embracing a healthier lifestyle and that’s what I need. (Divataunia, 13 days pre-surgery) (10)

Hey YouTube, it’s been so long since I’ve talked to you and I’m really sorry. I’ve been getting mail from everybody checking in with me which is really nice to know you guys are still interested in what’s going on with me. (Thebandinme, 9 months post-surgery) (10)

I just want to thank everybody, the best support and information I’ve gotten has been here on YouTube and I feel really thankful that this is a resource I’m able to use. Not only that, but just meet some really great people, you all are fantastic really and I mean that. (Divataunia, one week pre-surgery) (10)

What does it mean to you that’s like eating food, losing weight, being thin or being fat. (10)

How do you relate those things to who you are as a person, who you think that you are, and what you think you’re worth, and where you’re going. It’s weird, like before I was like oh I’m fat, lazy, that’s pretty much all I’ll ever be and so hmm I’m no prize, you know I don’t deserve a lot. And now it’s like wow I don’t have to be like that. (10)

I can go to school, and get a really good job, and have a stable relationship with someone I’m really crazy about and really attracted to, and have a cute little life and money, and self-confidence, and look cute and be attractive. Like holy shit that’s a lot of stuff to all of a sudden have to think about. Where before it was so easy to be like,meh I’ll never get there. So I think that’s why I react so emotionally to these little things because it’s like before I didn’t have to deal with them, I could just be like I’m fat, I need to diet and let that rule my life and my thoughts, like my thought process never got passed that. It was like oh if I could only lose weight. Well now I have and now what? . . . . I like taking time like this for myself to think about what’s going on, and where I’m at and who I am. (Thebandinme, 9 months post-surgery) (10)

I have control over what goes in my mouth and what happens with my body now and that’s the best thing that’s happened from this surgery besides the weight loss. (Divataunia, 5 months post-surgery) (10)

That’s the hugest part of lapband that was attractive to me. I get to control how much I eat, I get to control my appetite, I get to control my weight, I get to control my body, in a way that I’ve never been able to. (Thebandinme, 15 months post-surgery) (10)

‘It’s about me and making myself better’ (Thebandinme, 3 days pre-surgery); ‘The more I think about it the prouder I am of my decision to be proactive in my health and to really take charge of it and change my life for the better’ (Divataunia, 10 months post-surgery). ‘Being better’ explicitly means being able to do a multitude of things they did not feel able to do previously. (10)

I don’t feel like my body is holding me back mentally or physically anymore. And I still have about 40–50 pounds to go but just having 100 pounds off really makes such a huge difference in my life. In every way, my health, my stamina, my clothing, my self-esteem. (Divataunia, 5 months post-surgery) (10)

I have control over what goes in my mouth and what happens with my body now and that’s the best thing that’s happened from this surgery besides the weight loss. (Divataunia, 5 months post-surgery) (10)

That’s the hugest part of lapband that was attractive to me. I get to control how much I eat, I get to control my appetite, I get to control my weight, I get to control my body, in a way that I’ve never been able to. (Thebandinme, 15 months post-surgery) (10)

‘It’s about me and making myself better’ (Thebandinme, 3 days pre-surgery); ‘The more I think about it the prouder I am of my decision to be proactive in my health and to really take charge of it and change my life for the better’ (Divataunia, 10 months post-surgery). ‘Being better’ explicitly means being able to do a multitude of things they did not feel able to do previously. (10)

Yes I think I have already succeeded, I think the question is, can I maintain it? And that is where the struggle comes in . . . . There’s not anything that makes me sick. And that is good, but it’s also bad because it could lead me back to old behaviours of my past. (Divataunia, 8 months post-surgery) (10)

**Balancing body and mind tror att denna får byta namn när vi fått ihop texten lite mer (Body and mind integration?)**

Nowadays I am a more eager person, I feel like doing things and even go out from time to time…But the problem is dating…I just can't…..I'm ashamed…. P5 (8)

When I was 300 pounds, my skin fit me, and it wasn’t like I fooling anybody, I was fat,

that’s just the way it was . . . . But now that I’ve lost 110 pounds I’m starting to see the effects of that in my body and I’m not liking it at all. And I’m having a lot of insecurity issues which are driving me crazy because I’m turning into someone I don’t like. I’ve always been very confident and I’ve always been completely in control of my feelings about myself and I’m not now. I’m insecure about a lot of things, most importantly the way I look and the way people respond to the way I look. (Divataunia, 6 months post-surgery)

Partner: I think that your upcoming changes will only have a positive effect on your view of yourself, your confidence and everything (10)

Despite the desire to lose the fat and the fat sense of self that accompanied it, its loss does not necessarily immediately, or inevitably, produce a fresh or less complicated sense of who they are, nor what they may desire to become. (10)

I was uncomfortable being fat but that doesn’t mean that once you’re not fat you’re comfortable being skinny, it’s a totally different thing . . . . I guess I didn’t anticipate that. I always thought that as soon as I’m skinny I’ll feel exactly how I always wanted to feel and I’ll feel beautiful and confident and I won’t have to worry about people looking at me or me looking at myself in the mirror and feeling shitty or whatever. (10)

It’s not easy, it’s not pretty, there’s lots of struggles, people still with the issues that are up here [points to head] even after they’ve lost weight. And then there’s, like how do I feel about my body now that I’m skinny, am I ok with it now?And relationships, and anxiety, and emotional eating. (Thebandinme, 5 months post-surgery) (10)

Questions, including how do I maintain my new body, what does it mean for me and the way I relate to other people, must be addressed. Corporeal changes are not enough to ensure a subjectivity that works for them, psychological shifts are required too. (10)

Participant 108: “I still feel big and fat and I feel shocked when I look in the mirror or a shop window and see a slim body. This still has to sink in.” (1)

Participant 45: “The one thing I still struggle with is whether people are still looking at me because I am still fat or something. . . . I still can’t eat in public, because it feels like they are all staring at me and are thinking what she is eating, that’s so wrong.” (1)

Participant 108: “People approach me much more these days. But it gives me mixed feelings: Was I less worthy when I had a fat body?” (1)

Over a third of participants expressed appearance concerns, such as worries about excess skin and hair loss. A few women described that they had not adjusted to their slimmer body and expected to see a heavier person in the mirror. Others explained that they found it difficult to care for their appearance after years of self-loathing. (1)

Avoiding unwanted attention from others (1)

Valuing oneself based on society’s opinions on what is worthy in a person (1)

Participant 94: “We cooperate better and better, my body and I. . . . The surgery and the recovery made me aware that I should spend more time on and for myself. Listening to my needs and taking quality time for them. Now I just have to put this into practice. . . . ” (1)

Participant 49: “Feeling emotions is important but sometimes very difficult. Emotions and feelings are things that I like to put away.” (1)

Nearly a third of women described difficulties coping with emotions and trauma. Some described past traumas; others described that after surgery their emotional life had become tumultuous, and they had difficulties coping with this. Many women described barriers to sexual pleasure—for example, because they worried their partner would dislike their body. (1)

Over a third of participants expressed appearance concerns, such as worries about excess skin and hair loss. A few women described that they had not adjusted to their slimmer body and expected to see a heavier person in the mirror. Others explained that they found it difficult to care for their appearance after years of self-loathing. (1)

Several of the participants described a feeling of being alienated from themselves. Looking in a mirror or at a photo, they did not recognize the “other” person that they had become with the overweight: (2)

Yeah, kind of. I’m feeling like a stranger, almost, like, in my own body. Because I still see myself as the size I used to be, like, when I went to high-school, in the beginning of high-school before things started to slip. I’ve even kept clothes, as if, from back then, because that’s like, the size I’m supposed to be, and that really wasn’t slim but more the way I was comfortable. (Participant 4) (2)

I mean, I can go out and see people, but I kind of get stomach aches and I feel real sick, but it is possible, I mean, I do survive. ‘Cause before I met her (the CBT therapist) I couldn’t, but then I had both lost some weight and regained some of my self-esteem. I guess I think it’s difficult to tell if it depends on the weight or the self-esteem, but I think both go hand in hand in my case. (Participant 12) (2)

Several of the participants said that they no longer had thoughts of what other people might think of them and their body. This had previously restricted them in several everyday areas of their lives. Reflections about how it might have been all in their own head before came up, but nonetheless they now felt liberated from these worries. (3)

The participants described the process of finding themselves again. Some of them referred to the normal-weight person they had been before, which seemed easier than for those who never had a normal-weight version of themselves before. (3)

“It’s more that I’ve come back to what I should be like. Then I didn’t get this, like others did: “oh, I don’t think that I look slim” but I instead start to recognize myself again, like, “hey, this is me”. So, it’s a bit different to me.” Participant 4 (3)

“I guess that deep inside I’m always gonna be the fat one.” Participant 1 (3)

“I feel much better, I’m more comfortable. Like, I can be with other people. That’s fine. It doesn’t feel awkward anymore.” Participant 5 (3)

In people who have already faced bariatric surgery, feelings of failure seemed to arise from the memory of the past: (4)

‘I lived with difficulty’, ‘I remember moments of collapse’, ‘I threw my life to the wind’, ‘I tried and it did not change anything’. The experiences related to the preoperative period are, therefore, very similar in the two groups; however, one year after, the person describes herself differently. A new position related to the action emerged to the perception of ability and to the will of dealing with new situations. Participants now stated: ‘I assert myself’ or ‘I explore myself,’ ‘I can finally get out’, ‘I can go shopping,’ ‘I can be myself’. (4)

Regarding the relational area in the reoperative group, the ‘others’ were experienced as investigators, or persecutors: ‘Others have hurt me’, ‘I feel ashamed’, ‘People look at me’, ‘I do not feel accepted by some people’. The ‘other’ person was perceived as someone who does not recognise one’s own subjectivity. In the postoperative group, the ‘other’ stopped being perceived as a judge and the person finally feels as if she belongs amongst others, ‘to be accepted by others’, ‘I go out without being noticed’, ‘I’m normal’. (4)

Another semantic area that emerged only among participants, who had already been operated on, revealed frustration and difficulties in facing the consequences of surgery, was that of experiences related to the body changes. In this area, participants gave phrases like: ‘(. . .) it was hard, seeing so many changes and so quickly, is somewhat cruel (. . .)’, ‘(. . .) I did not expect that the change was so sudden (. . .) it was all too fast (. . .)’, ‘(. . .) I thought it would be easier (. . .).’ Statements that revealed inadequate preoperative preparation regarding the difficulty to adapt their selfimage to their new body: (4)

It ‘s true that you lose weight in the body, you cannot loose weight in the same way in your head or, anyway, certainly not at the same speed. (MB, postoperative group) (4)

I am an ex-obese (. . .) When I look in the mirror I see myself and I feel good-looking, . . . , when I try a dress, I can feel good. . . But when I think to myself, I guess I’m still chubby . . . maybe it happens to me to see some girls around and to think. . ., such a beautiful body, that envy! (4)

The mind has not yet adapted to the image of the new body and the new way of eating. Based on what we have presented above, it is not surprising that from the analysis of the dominance between I-positions, it emerged that in both groups the ‘I am obese’ was the dominant position, although physically normal in weight, the position ‘I am obese’ doesn’t become subject to another voice: it remains dominant, even among those who have already been operated on. (4)

Before (the surgery) I thought everything went wrong because of these extra pounds, I thought that everything depended on my obesity: if I stiffened in front of a guy, it was because I was ashamed of my body and because I was worried about what he could see in my body; (. . .) I just saw around me successful people, while I always remained there, large and lonely; if I could not wear nice clothes, if I could not wear makeup: it would be like seeing a whale walking with handbag and heels (. . .) But if I lost weight? (4)

Then yes, I would begin to live! Everything would be better! (. . .). (S. group post-operative) (4)

In some cases, the person has become aware that their body shape was covering other personal uncertainties and the weight loss only made other problems evident. In other cases, very favourable personal experiences testified the beginning of a different life phase: (4)

Under the flab I found a new me, with a different ability in relationships. (4)

I’ve become a less accommodating, less funny girl. I no longer need to make myself agreeable with filters that do not belong to me, in order to be appreciated. I became more reflective and less instinctual. (4)

In the postoperative group, answers appeared linked to the semantics of wellbeing: ‘I’m fine,’ ‘I feel good’ but the dominant voice was still ‘I am obese’. We also found the positions: ‘my family’ ‘my partner’ and ‘my work’ were significant. The latter position (the work) was completely absent in the preoperative group, which may indicate that the person was focussed on them before the operation and had no space for anyone else, while after the surgery the relational component becomes even more important. (4)

P4: The hardest thing was to get one’s brain in the right mindset, to get it converted…I knew what was needed but I just wasn’t geared for it. I would not succeed. It would just go bad again, why really straighten up, whenever things were going bad anyway. (6)

P3: Now I have to take care again, so it’s a rollercoaster. Now I have to be stable, so it’s still sometimes hard…yes, it may go up and down a bit. (6)

P3: I’m actually not the same person at all, as I was before surgery. Now, it’s me that means something, this means that I will continue being the person I am now. (6)

P3:…But now there is suddenly a lot of skin that I have to look at. I do not think is so funny. There is still something left, which means that you’re not quite there, where you have to be. But it is much better. I’m not finished yet. (6)

P4: It’s also something psychologically that has changed; you think more positively and believe that it is possible to dare something now. (6)

P5: You’re actually not hungry when you eat. Your brain keeps telling you that you are hungry. The stomach on the contrary is about to burst…and it’s hard to get rid of because your brain was operated on. This need, it’s not just removed in surgery…..There’s such a psychological need, all the time. (6)

When these reactions of others start to become evident, reactions of those very people whose “acceptance” they believed to guarantee by their getting thin, they can feel profoundly disillusioned, sometimes confused. (7)

“Before they did not like me because I was fat. Today I am thin …they will think that I am stealing their scene! I did not operate for this …” P4 (7)

Due to this perception, the risk of isolation is great, demanding special attention from the psychological team. (8)

I'm withdrawing....Before it was because I was obese, now am I withdrawing because I'm thin? P4 (8)

Now I'm no longer fat but I have flab, loose skin and everybody looks at me the same way. P6 (8)

Participants conveyed several positive elements of the postoperative period. They experienced changes in their everyday habits, and they could now ambulate without the hindrance of their excess weight. They also felt a relief that they no longer had to reflect on the size of a chair or if a clothing store would have their size. Several participants did express cynicism and shock over what they felt was an extreme superficiality in society, as well as patronising attitudes among healthcare professionals: (9)

I had to adapt to my new shell even though I was the same person on the inside, the people around me, their focus changed towards me, their way of talking to me, socialising with me, it was a bit tough to get used to (W5). (9)

It’s really sad because I can also think the same way sometimes, and its society’s socialisation that has made us this way (W4). (9)

But you look at yourself after losing 20 pounds and you’re like god I’m still fat, I still look at myself and see myself fat. And I can see the difference, I can see 20 pounds off of me but I still see a fat person. (14 days post-surgery) (10)

I just had this sort of wall up and it was really protective, that layer of fat protective and now its melting away its making me feel really scared, like vulnerable and sensitive and so I’m trying to deal with that. (Thebandinme, 5 months post-surgery) (10)

Like when I see fat girls I’m like what’s up ‘cause you’re my people. Like there’s this really fat girl in my maths class and I just want to hang out with her and talk like fat girl stuff! But then I feel like a fucking traitor because I was able to have surgery and lose a bunch of weight and now I’m not like obese anymore. I just feel like I betrayed them kind of. (Thebandinme, 9 months post-surgery) (10)

And how do I, how do I identify myself now? I don’t think I can be classified as a BBW anymore but I’m also not thin. I wish I didn’t have to have a label. But when you do personals online you have to say what your body type is. Uhh I hate it, I hate that you have to give a description. (5 months post-surgery) (10)

. . . all these people see me and I’m sure they just look at me like anybody else, you know like that’s just whatever like another slightly overweight girl and that’s really weird to me to just be another one of those girls. I don’t feel like another one of those girls, I still feel like a fat girl. (9 months post-surgery) (10)

. . . little changes every day are happening, they add up, they’re overwhelming, they’re wonderful, sorry I’m going to start to cry again, but it’s tricky. It’s tricky to figure out who you are, not just physically but emotionally and personality wise. Your whole life changes. (Divataunia, 8 months post-surgery) (10)

What does it mean to you that’s like eating food, losing weight, being thin or being fat. (10)

How do you relate those things to who you are as a person, who you think that you are, and what you think you’re worth, and where you’re going. It’s weird, like before I was like oh I’m fat, lazy, that’s pretty much all I’ll ever be and so hmm I’m no prize, you know I don’t deserve a lot. And now it’s like wow I don’t have to be like that. (10)

An outcome of this ‘identity crisis’ is the creation of a new self. Divataunia and Thebandinme regularly refer to and reflect on their ‘new’ and ‘old’ selves in a variety of ways. Divataunia speaks of ‘who we become after the surgery’ (14 months post-surgery) and how she is ‘mentally having a really hard time balancing my new life and my new struggles but with my old bad habits and demons’ (22 months post-surgery). (10)

This notion also resonated with thebandinme, ‘Us bandsters have a wonderful new life to enjoy’ (5 months post-surgery), mentioning ‘I’m a totally different person now than I was a year ago’ (6 months post-surgery). (10)

So this is sort of a goodbye to my overweight self. And a year from now I can look back and see what a transformation it’s been . . . . I just want to try and document everything I can for my view back on this journey. (13 days pre-surgery) (10)

. . . my ego is swelling a little bit but also at the same time I don’t know who I am, I don’t know if I feel good about myself but I guess that is the journey and I know a lot of people consider lapband surgery as a journey and an emotional and personality type journey where it really changes you and it’s hard to anticipate how it’s gonna change you and who you’re gonna end up being. (Thebandinme, 2 weeks post-surgery) (10)

Yes I think I have already succeeded, I think the question is, can I maintain it? And that is where the struggle comes in . . . . There’s not anything that makes me sick. And that is good, but it’s also bad because it could lead me back to old behaviours of my past. (Divataunia, 8 months post-surgery) (10)

I’m missing the person that I was before surgery. I know I’m still that person in a lot of ways but losing all that weight throws you for a loop you know. (Thebandinme, 9 months post-surgery) (10)

Their changing weight shapes a shift in their sense of themselves in the world. (10)

When I was 300 pounds my skin fit me, and it wasn’t like I fooling anybody, I was fat, that’s just the way it was . . . . But now that I’ve lost 110 pounds I’m starting to see the effects of that in my body and I’m not liking it at all. And I’m having a lot of insecurity issues which are driving me crazy because I’m turning into someone I don’t like I’ve always been very confident and I’ve always been completely in control of my feelings about myself and I’m not now. I’m insecure about a lot of things, most importantly the way I look and the way people respond to the way I look (Divataunia, 6 months post-surgery) Partner: I think that your upcoming changes will only have a positive effect on yourview of yourself, your confidence and everything (10)

Thebandinme: You think that, but a lot of people get really freaked out by it. Like people that were fat that had lapband surgery and like noticed that while they’re losing weight and after they’ve lost weight people are like way nicer to them and like treated them totally differently, and give them more opportunities and stuff like that and they’re just really disturbed by the sort of prejudices that fat people are faced with every day. And it’s true, there’s like so many pre-conceived notions and just the way that people approach fat people, it’s weird. Sometimes it can be really traumatizing to get skinny . . . . They’ll always think that they need to be thinner, they’re still fat, blah, it’s Body Dimorphic Disorder. (Thebandinme, 2 months post-surgery) (10)

Nearly a third of women described difficulties coping with emotions and trauma. Some described past traumas; others described that after surgery their emotional life had become tumultuous, and they had difficulties coping with this.(1)

“I., well since., because I’ve got this depression I’m not in the mood for intimacy., that is neither intercourse nor closeness at all, really. It feels like I’m rejecting him, which I don’t want him to., I mean, to feel. So, in that way it’s worsening the entire situation, so to say.” Participant 3 (3)

In the postoperative group, answers appeared linked to the semantics of wellbeing: ‘I’m fine,’ ‘I feel good’ but the dominant voice was still ‘I am obese’. (2)

Women in the preoperative group anticipated that their life could ‘magically’ change after the bariatric surgery. They found it difficult to distinguish between real and concrete life experiences that a thin body would allow them to live, from the more profound changes that do not depend on weight, such as those relating to friendships and relationships with the opposite sex. Some participants of the postoperative group, on the other hand, reported continuing to limit their lives. They imagined that so many experiences would change automatically with the weight loss, but it did not happen, because those experiences were related to their personal self-esteem: (4)

Participants described their lives before BS as a difficult period where they had daily thoughts about weight loss, feeling displeasure and dissatisfaction with their body and appearance. They sed avoidance behaviours to protect themselves. This meant avoiding mirrors, not creating awareness around their personalities and a lack of desire to meet new people. Participants had low self-worth and self-esteem. (6)

On leaving the “cocoon,” represented by the excess of body fat, there is a feeling of lack of protection and for this reason, at the same time that they achieve this condition of being admired, phobic symptoms appear. (8)

Some of that’s true, I feel good when I look in the mirror. (Thebandinme, 3 months post-surgery)

As their bodies change, so too does their sense of self, as illustrated in the following section. (10)
