# Supplementary material for: Impact of bariatric surgery on premenopausal women’s womanliness: A qualitative systematic review and meta-synthesis
Source: PLoS One. 2024 Aug 29;19(8):e0308059. doi: 10.1371/journal.pone.0308059 (PMC11361607; doi:10.1371/journal.pone.0308059)
Supplement: S4 Dataset — (DOCX) [file pone.0308059.s008.docx]

**Womanliness**

Women stated a return to femininity after the weight loss. The reduction of extra weight with a return to female pattern fat distribution made them feel womanlier and they enjoyed purchasing clothing that brought attention to their new figures. The women experienced a newfound interest in caring for themselves since their return to womanliness made them more motivated to care for themselves health-wise and concerning their outer appearances. The weight loss made them strengthen their self-appreciation and care for themselves, and many women felt that they could finally express themselves the way they wanted to. With subsequent weight loss, the surgery lifted their self-confidence and motivated the women to express themselves, be assertive and even demand more of others. Before the surgery, the women hid within their excess weight and often portrayed a façade of joy and humor to hide their sadness. The weight loss was metaphorically likened to a cocoon being removed, and instead, they presented their true outer selves. A metamorphosis and new start in their lives occurred.

*In contrast, almost a third of women described increased appearance evaluation and investment post-surgery, such as enjoying clothes shopping and feeling more satisfied with their appearance. Almost a third described social freedom and confidence and felt more accepted in social gatherings. (1)*

*Women stated that weight loss and having a slim physical appearance gave them more freedom in what to wear and increased their self-esteem after surgery. Some women described the desire to look in the mirror and like what they see. (5)*

The increased self-esteem that the women experienced from their weight loss also improved their relationships. The women became more social and enjoyed contact with family, friends and their partners in a completely different way. Prior to weight loss, the women often lacked the energy and endurance to take part in social activities however, the changes after surgery and weight loss motivated the women and brought about positive emotions so that contact with other people was enjoyable. The women also felt an improvement in their relationships with their partners, who were positively influenced by the changes in their partner’s moods and stamina. The women also felt that their partners showed a new appreciation for them and that their increased self-esteem and positive mood motivated their partners to become closer to them.

*I want to date and be this new person. I think that’s the struggle in maintaining a relationship, you feel like you got a second chance on life. You just reinvented yourself and you reinvented what you want, what you want out of life, what you expect from other people, what you expect from yourself. I think all of that stuff is different for me than a year ago. (Thebandinme, 1 year post-surgery) (10)*

*We also found the positions: ‘my family’ ‘my partner’ and ‘my work’ were significant, while after the surgery, the relational component becomes even more important. (4)*

The women were no longer ashamed of their bodies and instead enjoyed sexual activity with their partners. They had previously avoided sexual activity out of shame for the way that they looked and their physical attributes. Their partners were previously shunned and pushed away as the women kept a distance due to embarrassment from their appearance. The weight loss changed their self-esteem, making them want to show themselves to their partners. The women described a feeling of womanliness in wanting to take part in sexual activity and that this strengthened their relationships with their partners.

*Most of the participants described a more active sex life, which was also more satisfying than before surgery. Internal factors, such as being more comfortable in a sexual situation and enhanced self-esteem, allowed them to demand more of their partners. (3)*

*In addition to experiencing an increase in sexual desire after the surgery, some women stated that their husbands’ sexual desire also increased, and having more satisfaction due to having more options in movements and motion during intercourse (5)*

The women felt a return to normality and womanliness when their hormones returned to physiological regulation The weight loss and normalization of sex hormones became obvious to the women as their menstrual cycles became regular and they experienced ovulation symptoms. The women now had new hope that they would be able to achieve their dreams of becoming pregnant and having a family once their fertility was restored. They felt that the surgery was an important step and solution to their previous difficulties in becoming pregnant. They also described the satisfaction of finally feeling like women when they had regular menstruation, felt their ovulations, and had improved sexual function. The surgery provided the steps needed to achieve their goals of becoming mothers and having a family.

*Another participant pointed out less need for lubricants as the cycle had become more regular and ovulatory. Yet another said that the weight loss had increased “the feeling of having intercourse.” (3)*

*Before I felt that I had a lack of female hormones, I felt that nothing worked as it should, and now, when everything works, it feels like a whole new world, I feel like a woman now (W9). (9)*

*Through bodily, social and mental well-being, all participants considered the opportunity of realizing their dreams of having children and raising a family (6)*

There were some negative experiences related to weight loss and relationships. The women experienced that their husbands could express jealousy and fear that other people would find their wives attractive. There became an imbalance in the relationship when the women became empowered with self-esteem and confidence after their weight loss. Certain partners expressed a fear that their wives would leave them after losing weight and gaining self-assurance. Some women had the perception that the weight loss’s positive qualities could lead to more destructive experiences in their relations.

*After all the effort to find their place in the world again and to be admired, they have faced the reappearance of their feminine bodies: a new situation with which they are unable to cope. (8)*

*In the postoperative period, the women have to face new life experiences, such as jealousy, mistrust, fear, and envy that, until recently, had not existed. (7)*

**A healthy and functioning body**

The women experienced a restoration to a healthy body after surgery. The weight loss led to a decreased burden on their bodies, and they were able to exercise and activate themselves effectively. The women experienced an increase in energy and stamina that allowed them to take part in life in a new way. They were able to be active parents and enjoyed their children. Feelings of guilt no longer impeded them for not being able to be involved in their family lives. Those not yet having children now felt prepared to take on the role of a good mother.

*Participant 45: “Before the operation, I could not experience much fun. I was always tired and not in the mood to go somewhere. Now I have lost 27 kilos. I enjoy going out with my husband and daughter, especially because I am less tired.” (1)*

The weight loss and newfound appreciation of their bodies made them more energized to take part in social activities. Being more social and open to others reduced feelings of isolation and abandonment that they had previously experienced prior to surgery. The weight loss made it possible for the women to be themselves and express themselves in a way that they had previously not had the courage to do.

*“Now, I no longer feel like it’s uncomfortable to go exercising among other people, like they’d be thinking “what is she doing” and so on. I can do that. I am much, much more comfortable in social contexts, like I said. Going to birthday parties and, like hanging out with friends and so on, that feels great too.” Participant 10 (3)*

*The first source of relief, after postsurgical recovery, comes from a strong sensation of acceptance and social reinsertion. They feel that they are part of a world which they were not a part of. They experience a feeling of genuine happiness. (7)*

The women realised a need to be more aware of the side effects of surgery and to be able to read the body’s signals concerning meal sizes and proper food composition. The women were also concerned with the risk of gaining weight again after surgery and experienced a fear of losing the joy and relief that they experienced after weight loss. There was a need to maintain control of their situation and not let their past return to them.

*P1: I hope that I manage to stay on the right path and I believe in that. When you have become so pleased with yourself and you know how it would be if you fell back, then…. (6)*

The change in their bodies made it possible to see life and live in a positive way. They appreciated their bodies and became more aware of their body functions, describing a clearer awareness of their body’s physical signals and living a healthier lifestyle since they were more in tune with their physical state. The changes that occurred after surgery and the side effects that they needed to be aware of made them pay more attention to the body’s signals.

*I have control over what goes in my mouth and what happens with my body now and that’s the best thing that’s happened from this surgery besides the weight loss. (Divataunia, 5 months post-surgery) (10)*

**Mind and body connection**

The women felt that they often had identified themselves as obese and had difficulty adapting themselves to a life as a normal-weight person. The psychological effects of living with obesity could remain and continue to affect their mood and opinions of themselves. There was a need for psychological support to deal with the changes, often long after the surgery. They could often feel discomfort in public, fearing that others may scrutinize them even though they had attained a normal weight.

*Over a third of participants expressed appearance concerns, such as worries about excess skin and hair loss. A few women described that they had not adjusted to their slimmer body and expected to see a heavier person in the mirror. Others explained that they found it difficult to care for their appearance after years of self-loathing. (1)*

*P5: You’re actually not hungry when you eat. Your brain keeps telling you that you are hungry. The stomach on the contrary is about to burst…and it’s hard to get rid of because your brain was operated on. This need, it’s not just removed in surgery…..There’s such a psychological need, all the time. (6)*

The women experienced a delay in understanding that they had lost weight and had a new appearance. When they finally understood the change, they took pride in shopping and presenting their bodies with new clothes. They also saw their changed appearance as a new beginning and a chance to make a new start in their lives and health. They used their new identity to create a newer, healthier, and better-functioning version of themselves.

*Several of the participants said that they no longer had thoughts of what other people might think of them and their body. This had previously restricted them in several everyday areas of their lives. Reflections about how it might have been all in their own head before came up, but nonetheless they now felt liberated from these worries. (3)*

*‘I lived with difficulty’, ‘I remember moments of collapse’, ‘I threw my life to the wind’, ‘I tried and it did not change anything’. The experiences related to the preoperative period are, therefore, very similar in the two groups; however, one year after, the person describes herself differently. A new position related to the action emerged to the perception of ability and to the will of dealing with new situations. Participants now stated: ‘I assert myself’ or ‘I explore myself,’ ‘I can finally get out’, ‘I can go shopping,’ ‘I can be myself’. (4)*

*P3: I’m actually not the same person at all, as I was before surgery. Now, it’s me that means something, this means that I will continue being the person I am now. (6)*

The women also experienced certain difficulties in dealing with how people and society treated them after weight loss. People could express jealousy at their ability to lose weight or envy their new figures. They also felt despair in experiencing the changed attitudes of others towards them after they lost weight and seeing clearly how differently people are treated based on their body size and appearance.

*“Before they did not like me because I was fat. Today I am thin …they will think that I am stealing their scene! I did not operate for this …” P4 (7)*

*I had to adapt to my new shell even though I was the same person on the inside, the people around me, their focus changed towards me, their way of talking to me, socialising with me, it was a bit tough to get used to (W5). (9)*

The sudden weight loss and subsequent change in appearance could have profound effects on women. They dealt not only with their own identities but also with belonging to a certain group. The women had identified themselves as obese for a large portion of their lives and accepted certain factors related to living with obesity. They often lived with shame, fear and depression related to the struggles of living with reduced physical capacity, health problems and the stigma from society. The profound and often sudden weight loss created a severe change in their appearance that could challenge their opinion and acceptance of themselves.

*I am an ex-obese (. . .) When I look in the mirror I see myself and I feel good-looking, . . . , when I try a dress, I can feel good. . . But when I think to myself, I guess I’m still chubby . . . maybe it happens to me to see some girls around and to think. . ., such a beautiful body, that envy! (4)*

The women had previously belonged to groups that promoted pride in having a large body and admonishing a need to fit in with a thin body. They felt that they had let that group down and instead become part of the adversary, at the same time that they enjoyed their weight loss and new figures. They needed to find a way to accept their new group and identity without disappointment.

*Like when I see fat girls I’m like what’s up ‘cause you’re my people. Like there’s this really fat girl in my maths class and I just want to hang out with her and talk like fat girl stuff! But then I feel like a fucking traitor because I was able to have surgery and lose a bunch of weight and now I’m not like obese anymore. I just feel like I betrayed them kind of. (Thebandinme, 9 months post-surgery) (10)*
