# Supplementary material for: Impact of bariatric surgery on premenopausal women’s womanliness: A qualitative systematic review and meta-synthesis
Source: PLoS One. 2024 Aug 29;19(8):e0308059. doi: 10.1371/journal.pone.0308059 (PMC11361607; doi:10.1371/journal.pone.0308059)
Supplement: Attachment — Submitted filename: Response to reviewers.docx [file pone.0308059.s009.docx]

Jennifer Tucker, PhD
Associate Editor
PLOS ONE

Dear Dr. Tucker,

Thank you for the valuable feedback and comments. We have made several changes according to reviewers’ suggestions and feel that our manuscript has been greatly improved.

Journal requirements:

1. Thank you for highlighting necessary adjustments concerning style requirements, we have reviewed our manuscript and corrected any deviations from the described instructions.
2. We include the following statement to our Competing Interests section since we have no restrictions on sharing of data and materials: “This does not alter our adherence to PLOS ONE policies on sharing data and materials.”

Reviewers’ comments

1. Thank you for your responses.
2. Thank you for your responses.
3. Thank you for your responses.
4. Thank you for your responses.
5. Reviewer #1

Why did you choose "particularly bypass"? I think that for the scope of your work other types of surgery could be included, as Sleeve for example.

*Thank you for your relevant remark. We chose a primary focus on gastric bypass since this procedure has, until relatively recently, been the most common procedure in bariatric surgery and our previous studies included patients having undergone Roux-en-Y gastric bypass surgery.*

*We did, however, include studies that involved other forms of bariatric surgery such as vertical sleeve gastrectomy and gastric banding in order to broaden our findings. As sleeve gastrectomy is the most prevalent bariatric procedure worldwide, we do agree that it would have been valuable to include more studies with patients who have undergone this procedure.*

*However, the only relevant qualitative studies found in this review predominantly involved gastric bypass surgery with a minority of participants having undergone sleeve or banding. This may have been a consequence of our inclusion criteria involving qualitative research method, exclusively female participants and with focus on postoperative experiences.*

What did you mean by "through limits another 3,421 articles were removed"?

*We applied limits based on our inclusion criteria such as “women” “humans” and publishing dates that reduced the included total of articles by 3,421. The inclusion criteria of exclusively premenopausal women, publication date 2000-2023 and exclusively qualitative or mixed methods studies also greatly reduced the number of relevant studies that could be included in the analysis.*

Although the science behind meta-synthesis says: "Approximately 10 to 20 studies represent an ideal meta-synthesis", I am concerned about your data using only 10 papers from 5,168 and, many of them with very few patients. Can you better explain and justify it beyond GRADE-CERQual?

*We fully understand the reviewer’s concern. However, the search strategy was broad in order to not miss any relevant papers. The few studies involving qualitative research method, exclusively female participants and focus on postoperative experiences led to the reduction from 5,168 studies to 10 relevant articles that correspond to our inclusion criteria.*

The ten included studies utilizing findings accumulated from 156 women. The data included in the meta-synthesis are quotations directly from participants and the researcher’s interpretation. Text excerpts were extracted and reviewed prior to meta-synthesis in order to determine if a necessary richness and depth of data was available in order to carry out a meta-synthesis. We determined that the amount of data from the 10 included studies was sufficiently abundant with data richness to allow an analysis that involved the four criteria of trustworthiness in qualitative research (dependability, credibility, confirmability, and transferability [1]. In Table 2, we display the confidence of our study findings according to GRADE-CERQual for our results. This provides evidence that the result is grounded in the included studies, thereby representing a more significant number of women than the ordinary small sample size in qualitative studies.

Table 1 is very busy and makes your manuscript longer. Is it really necessary?

*We appreciate your comments. Table 1 has been removed from the manuscript and included as a supplement (S4) in order to shorten the manuscript and make it more concise.*

Table 2 should be transformed in text. As a table, it is hard to read.

*Thank you for your comments, and we appreciate the large amount of text involved in the descriptions in Table 2. However, we feel it is necessary to include Table 2 in the manuscript in order to provide easily accessible transparency concerning our findings.*

Reviewer #2

Major issues
Methods
162 I'd like to know a bit more about how the data extraction was made. Did you not use any software? It might be clarifying to see an example of the data extraction form as an illustration.

*Thank you for your question concerning data extraction. We did not use computer software for the data extraction process. We utilized a standardized data extraction form that has been used in previous studies and references to these studies have been provided. We have included an example (Table 1) in order to facilitate understanding of the data extraction process.*

Table 1
I do not understand why the references have different numbering here and in the reference list. This makes it difficult to follow as to which study supports which findings.

*Thank you for noting this error which occurred during formatting. We have now made the necessary adjustments and revised Table 1 as supplementary information S4. Since Table 1 has become supplementary information S4 auxiliary to the manuscript, the changes are not marked with the tracking changes tool.*

Minor issues
Scrutinize language!
Line
61-62 This sentence is difficult to understand. 63 having obesity? 116 utilising? 426 This sentence is difficult to understand.
433 “a” word missing? 561 Reference needs check-up.

*Thank you for your comments, we have scrutinized the language and made several modifications as well as the suggested adjustments and clarifications*. *The reference has been amended.*

On behalf of all authors,

Rebecca Paul, M.D., Ph.D.

Department of Biomedical and Clinical Sciences, Linköping University, Sweden

References

1. Guba EG. Criteria for assessing the trustworthiness of naturalistic inquiries. ECTJ. 1981;29(2):75. doi: 10.1007/BF02766777.
