# Supplementary material for: Impact of bariatric surgery on premenopausal women’s womanliness: A qualitative systematic review and meta-synthesis
Source: PLoS One. 2024 Aug 29;19(8):e0308059. doi: 10.1371/journal.pone.0308059 (PMC11361607; doi:10.1371/journal.pone.0308059)
Supplement: Attachment — Submitted filename: Response to reviewers.docx [file pone.0308059.s010.docx]

Annesha Sil, PhD
Associate Editor, PLOS ONE

Dear Dr. Sil,

Thank you for your careful review and recommendations concerning our manuscript,

 " Impact of bariatric surgery on premenopausal women's womanliness: A qualitative systematic review and meta-synthesis." Your recommendations, previous editors, and reviewers have dramatically improved our paper. Below, we respond to comments.

**Journal Requirements:**

 Please review your reference list to ensure that it is complete and correct. If you have cited papers that have been retracted, please include the rationale for doing so in the manuscript text or remove these references and replace them with relevant current references. Any changes to the reference list should be mentioned in the rebuttal letter that accompanies your revised manuscript. If you need to cite a retracted article, indicate the article's retracted status in the References list and also include a citation and full reference for the retraction notice.

*We have reviewed our references and found none that have been retracted. We did find an erratum in one reference, which we have included in the reference list.*

**Additional Editor Comments:**

After careful reviewing, before we can recommend acceptance, we would kindly request you to address a minor outstanding concern regarding terminology.

Specifically, we note that the term "womanliness" appears throughout the manuscript. However, we note that the term does not appear to be associated with a concrete definition, and moreover, several themes as discussed in the womanliness section (beginning ln 228) do not appear to match the provided definition.

 We would therefore kindly request that you update your submission to either a) replace or qualify the theme 'womanliness' (as stated on ln 228) with a description that more broadly encompasses the themes discussed in this section, or b) create an additional section that encompasses the themes, in addition to the existing header 'womanliness' that is based on the definition provided and labelled accordingly.

*We have now clarified and defined the term womanliness in order to enable a clearer understanding of the fusion resulting from the meta-analysis of the included studies.*

 Reviewers' comments:

**Comments to the Author 1-7**

***Thank you for reviewing our manuscript and providing useful recommendations that have greatly improved it.***

On behalf of all authors,

Rebecca Paul, M.D., Ph.D. Department of Biomedical and Clinical Sciences, Linköping University, Sweden
